# Systemically Circulating Viral and Tumor-Derived MicroRNAs in KSHV-Associated Malignancies

**DOI:** 10.1371/journal.ppat.1003484

**Published:** 2013-07-18

**Authors:** Pauline E. Chugh, Sang-Hoon Sin, Sezgin Ozgur, David H. Henry, Prema Menezes, Jack Griffith, Joseph J. Eron, Blossom Damania, Dirk P. Dittmer

**Affiliations:** 1 Lineberger Comprehensive Cancer Center, Program in Global Oncology, Department of Microbiology and Immunology, University of North Carolina at Chapel Hill, Chapel Hill, North Carolina, United States of America; 2 Department of Oncology, Joan Karnell Cancer Center, University of Pennsylvania, Philadelphia, Pennsylvania, United States of America; 3 Department of Infectious Diseases, University of North Carolina at Chapel Hill, Chapel Hill, North Carolina, United States of America; University of Southern California Keck School of Medicine, United States of America

## Abstract

MicroRNAs (miRNAs) are stable, small non-coding RNAs that modulate many downstream target genes. Recently, circulating miRNAs have been detected in various body fluids and within exosomes, prompting their evaluation as candidate biomarkers of diseases, especially cancer. Kaposi's sarcoma (KS) is the most common AIDS-associated cancer and remains prevalent despite Highly Active Anti-Retroviral Therapy (HAART). KS is caused by KS-associated herpesvirus (KSHV), a gamma herpesvirus also associated with Primary Effusion Lymphoma (PEL). We sought to determine the host and viral circulating miRNAs in plasma, pleural fluid or serum from patients with the KSHV-associated malignancies KS and PEL and from two mouse models of KS. Both KSHV-encoded miRNAs and host miRNAs, including members of the miR-17–92 cluster, were detectable within patient exosomes and circulating miRNA profiles from KSHV mouse models. Further characterization revealed a subset of miRNAs that seemed to be preferentially incorporated into exosomes. Gene ontology analysis of signature exosomal miRNA targets revealed several signaling pathways that are known to be important in KSHV pathogenesis. Functional analysis of endothelial cells exposed to patient-derived exosomes demonstrated enhanced cell migration and IL-6 secretion. This suggests that exosomes derived from KSHV-associated malignancies are functional and contain a distinct subset of miRNAs. These could represent candidate biomarkers of disease and may contribute to the paracrine phenotypes that are a characteristic of KS.

## Author Summary

Circulating microRNAs (miRNAs), such as those found in exosomes, have emerged as diagnostic tools and hold promise as minimally invasive, stable biomarkers. Transfer of tumor-derived exosomal miRNAs to surrounding cells may be an important form of cellular communication. Kaposi's sarcoma-associated herpesvirus (KSHV) is the etiological agent of Kaposi's sarcoma (KS), the most common AIDS-defining cancer worldwide. Here, we survey systemically circulating miRNAs and reveal potential biomarkers for KS and Primary Effusion Lymphoma (PEL). This expands previous tissue culture studies by profiling clinical samples and by using two new mouse models of KSHV tumorigenesis. Profiling of circulating miRNAs revealed that oncogenic and viral miRNAs were present in exosomes from KS patient plasma, pleural effusions and mouse models of KS. Analysis of human oncogenic miRNAs, including the well-known miR-17-92 cluster, revealed that several miRNAs were preferentially incorporated into exosomes in our KS mouse model. Gene ontology analysis of upregulated miRNAs showed that the majority of pathways affected were known targets of KSHV signaling pathways. Transfer of these oncogenic exosomes to immortalized hTERT-HUVEC cells enhanced cell migration and IL-6 secretion. These circulating miRNAs and KS derived exosomes may therefore be part of the paracrine signaling mechanism that mediates KSHV pathogenesis.

## Introduction

MicroRNAs (miRNAs) are small, non-coding RNAs that are capable of fine-tuning gene expression through translational repression and/or mRNA degradation. In the past, miRNAs have emerged as important regulators in nearly every cellular process, but perhaps the largest biological consequence of miRNA dysregulation is in cancer [1], [2], [3], [4], [5], [6]. The relationship between intra-tumor miRNA signatures and cancer progression has been well established, leading to the discovery of specific miRNAs or miRNA clusters that modulate gene expression in cancer [7], [8], [9]. We and others have shown that miRNA signatures can classify tumors into distinct classes and are predictive of disease outcome [3], [4], [6], [10], [11]. In our prior study, we found that the host miRNA profile differed depending on the degree of transformation among cells, even though all samples were infected by the same virus and thus expressed similar levels of viral miRNAs [6]. This suggests that host miRNA profiles impart information about viral infection above that provided by detecting the presence of the infectious agent.

MiRNA regulation is complex in malignancies associated with viral infection such as herpesvirus-associated cancers [2], [6], [12], [13]. Viral infection can trigger changes in the miRNA profile through the expression of viral genes that modulate the host miRNA repertoire. Some viruses such as Kaposi's sarcoma-associated herpesvirus (KSHV) and Epstein-Barr Virus (EBV) in addition encode their own miRNAs, which fine-tune host gene expression to promote latent viral persistence, immune evasion, and tumor progression [8], [9], [14], [15], [16], [17]. These viral miRNAs are often expressed within the tumor and can reveal important information regarding viral latency and disease progression [18]. Furthermore, recent studies have highlighted important functions of the viral miRNAs in regulation of the viral life cycle, immune evasion and angiogenesis through validated mRNA targets [7], [14], [19], [20], [21], [22], [23]. In KSHV-associated cancers, the KSHV miRNAs can account for as much as 20% of all mature miRNA species within a cell and are highly conserved among isolates (Figure S1 and [12], [14], [17]).

KSHV is the etiological agent of Kaposi's sarcoma (KS), the most common AIDS-defining cancer worldwide [24]. KSHV is also associated with the B cell lymphoma Primary Effusion Lymphoma (PEL) and with the plasmablastic variant of Multicentric Castleman's Disease (MCD). Despite the availability of Highly Active Anti-Retroviral Therapy (HAART), KS continues to occur in the US and worldwide. Treatment of KS remains a challenge and stable, minimally invasive biomarkers for diagnosis are lacking [25], [26]. Therefore, the discovery of plasma miRNA biomarkers for KSHV-associated malignancies could improve diagnostics through early detection and could influence treatment through non-invasive monitoring of tumor responses. MiRNA biomarkers can be sampled from blood, saliva, or other bodily fluids, offering a feasible diagnostic test even in resource-poor regions such as the “KS belt” in sub-Saharan Africa [24], [27]. Viral microRNAs are the most attractive candidate biomarker because of their specificity for KSHV. However, a combination of viral microRNAs with cellular microRNA biomarkers is even more useful, as it may help differentiate among stages of KS progression or response to therapy and as it can identify cellular microRNAs that are common among KS and other cancers. We previously determined the cellular and viral miRNA profile in KS tumor biopsies as well as in PEL and found that the expression of viral miRNAs varies with disease state [3], [4], [6]. In addition to the viral miRNAs, key cellular miRNAs are involved in KSHV transformation and KS progression [8], [9], [28], [29].

The detection of circulating miRNAs in plasma, serum and other bodily fluids suggests their utility as minimally invasive biomarkers for cancer diagnostics [11], [30], [31], [32], [33], [34]. These circulating miRNAs are unusually stable (i) due to their packaging in microvesicles or exosomes, (ii) due to their RNA folding and size and/or (iii) due to their presence in Ago-containing ribonucleic acid:protein (RNP) complexes [32], [34], [35], [36]. At this point it is unclear which of these mechanisms is the most efficient. Evidence suggests that all three mechanisms contribute to diagnostic utility by increasing miRNA stability. There are a variety of vesicles that are secreted from cells, each with slightly varying content and surface marker composition. Microvesicles can range in size from 30 nm–1000 nm and each follow different pathways of biogenesis (reviewed in [37], [38], [39]). Recent studies have additionally shown that microvesicles from tumor cells may have altered morphology, size and surface markers, including the expression of tumor antigens compared to microvesicles that are released from non-tumor cells [40], [41], [42], [43]. MiRNAs have been detected in microvesicles, exosomes and/or nanovesicles. This study refers to these vesicles collectively as exosomes based on common surface marker expression and morphological characteristics.

Transfer of exosomes and their contents from tumor cells to surrounding, uninfected cells may be an important form of cellular communication and has been demonstrated in cell culture models, for instance in EBV-associated cancers [44], [45]. Additionally, exosomes may provide a means of paracrine signaling from virally infected cells to adjacent, non-permissive cells [46]. This study attempts to bridge the gap between clinical samples and cell culture models. To do so we compared the detailed, circulating miRNome of KS in clinical human samples and in KS mouse models [47], [48], [49]. This confirms the presence of circulating KS and KSHV-specific miRNAs *in vivo* in the context of KSHV infection. Multiple KSHV miRNAs and members of the miR-17-92 cluster of cellular miRNAs were detected within patient exosomes. These circulating miRNA signatures may serve as a new mechanism of paracrine signaling for mediating KSHV pathogenesis and may represent a reservoir for novel biomarkers.

## Results

### Clinical samples and mouse models of KSHV-associated malignancies

To date, most studies on viral exosomes have used tissue culture models of infection. To expand on these studies, we utilized a series of clinical samples and two novel robust mouse models of KSHV pathogenesis [47], [48], [49]. The sample groups and number of samples included in each group are outlined in Table S1. Briefly, human plasma from healthy, KSHV-negative controls or from AIDS patients with either KS or a non-KS malignancy was used to isolate exosomes. The HIV viral load and CD4+ T cell counts were similar in both KS and non-KS malignancy groups (data not shown). KS tumor biopsies and primary PEL pleural fluid were also included and served as positive controls for the presence of KSHV compared to control human plasma. We also used two mouse models previously characterized in our lab [47], [48], [49]: the 801 latency locus transgenic mouse model which expresses all viral miRNAs in B cells [50]; and a xenograft model using TIVE L1 tumor cells, which maintain KSHV [48]. These cells are xenografted into SCID mice, which results in robust and reproducible tumor formation [48]. H&E staining revealed similar phenotypes of KS and our TIVE xenograft mouse model while both of these differed from the staining observed in PEL (Figure S2).

The KSHV-TIVE model [48] represents another instance of extended yet incomplete KSHV lytic transcription, as recently demonstrated in KSHV-infected lymphatic endothelial cell cultures under puromycin selection [51] and previously in KSHV-infected mouse endothelial cells [52]. Similarly, a KSHV cell line model of transformed rat mesenchymal precursors yields some lytic gene expression but with minimal amounts of virions produced [53]. The KSHV-TIVE endothelial cell model maintains KSHV in the absence of selection and like other long-term KSHV-infected endothelial cell cultures they remain tightly latent. Neither sodium butyrate nor exogenously provided RTA/Orf50 are able to induce infectious virus production (R.Renne, personal communication) or complete, genome-wide lytic transcription in TIVE L1 cells [48]. Subcutaneous implantation into mice can activate many viral genes, although these represent only approximately half of the genes turned on during lytic reactivation in PEL cells and are insufficient to produce infectious virions. A similar, abortive lytic expression profile has been observed in KSHV-infected human TIVE-L1 cells [48] as well as in KSHV-infected mouse and rat endothelial cells [52], [53]. This incomplete transcription program is incompatible with virion production and in the case of KSHV-infected LEC has been termed a novel latency program [51]. For this reason, we refer to the TIVE xenograft mouse model as a latent KSHV model due to the lack of virions produced.

### Exosome purification and analysis of miRNAs

**Figure 1:**
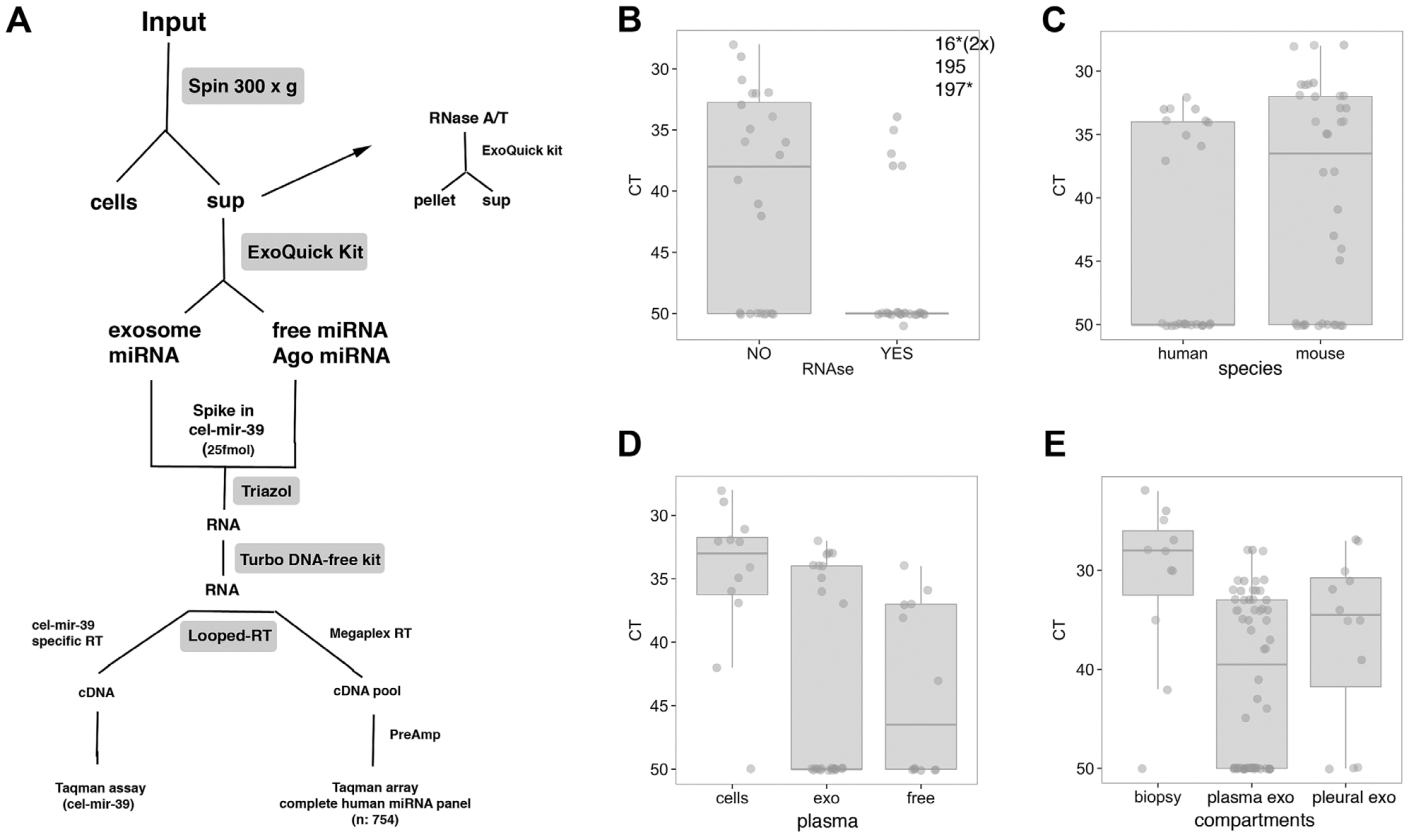
Exosome purification and analysis of miRNAs in sample subsets. Exosomes were purified and miRNAs isolated from exosome samples were analyzed for expression in various subsets. (A) Schematic for profiling of circulating miRNAs from plasma, serum and pleural fluid samples. (B–E) Box plots show the distribution of relative levels (CT) for 12 miRNAs for various conditions (mir-106b, mir-150, mir-16, mir-195, mir-197, mir-205, mir-23a, mir-30c, mir-425-5p, mir-548a, mir-92a, U6 snRNA). We selected those miRNAs, as they were highly expressed and as being representative of the different patterns we see across the experimental controls. Two independent experiments were performed and both replicates are shown. The line represents the median expression of microRNAs for a given sample group while individual microRNAs are denoted by closed circles (n = 24). In some cases, the median of the group is equal to 50 and the line is along the x axis due to >50% of miRNAs with a CT = 50. MiRNA expression following RNase treatment of control human plasma supernatants (B), comparison of human and mouse exosomal miRNA expression in control human plasma and mouse serum (C), differential expression in purified subsets from control human plasma (D) and tissue-specific expression (E) are shown. Asterisks denote previously detected plasma miRs [35].

Exosomes and circulating miRNAs were purified as shown in Figure 1A and detailed in methods. Following purification, total RNA was isolated from each sample group and used for Taqman-based qPCR profiling of the cellular miRNA repertoire (754 human miRNAs) as described [3], [4], [54]. Agilent RNA analysis showed that exosomes expressed small RNAs but lacked both 18S and 28S ribosomal RNA (Figure S3). Figure 1 shows the distribution of miRNAs in different sample subsets (Figure 1B–E). Each boxplot shows the expression levels for the different sample groups. The expression of individual microRNAs are denoted by solid circles. As demonstrated in Figure 1B, the majority of miRNAs present in control human plasma (KSHV−) in the supernatant fraction are susceptible to RNase, representing free, circulating miRNAs. These miRNAs are likely not encapsulated in Ago-RNP complexes nor microvesicles [35]. The exceptions were miRNAs miR-16, miR-195 and miR-197, which could be detected despite RNase treatment. This RNase resistance of these particular miRNAs is consistent with prior observations [35]. Levels of the *C. elegans* cel-mir-39 spike-in were abolished ∼16,000-fold after RNase treatment and were decreased when incubated with pleural fluid prior to RNA isolation (Figure S4). This verifies the activity of our RNase treatment and confirms that pleural fluid, like other body fluids, has some intrinsic RNase activity [33], [34], [35]. Therefore, the majority of RNAs that are stable in plasma and pleural fluid are likely RNase-resistant and protected within exosomes.

In samples enriched for exosomes derived from either control human plasma or mouse serum, we were able to readily detect both human and mouse miRNAs (Figure 1C). Figure 1D denotes the relative expression levels of miRNAs in cells, exosomes and the free, circulating fractions of control human plasma. As expected, exosomal and other circulating miRNAs are detectable but are present at lower levels compared with intracellular miRNAs. MiRNAs are readily detected in all sample types tested including tumor biopsies and exosomes from control plasma or serum and malignant effusions such as pleural fluid (Figure 1E), though the miRNA yield was highest in tumor tissue. Although we used human plasma and serum from mice in the majority of experiments, we also performed miRNA profiling with control mouse plasma. Importantly, we did not observe significant differences in the levels of miRNAs found in plasma versus serum in this study. The comparison of this small subset of miRNAs across the different variables shown in Figure 1B–E did not afford us the statistical power to identify differences among individual miRNAs expressed in these samples. However, these data establish the framework for further analysis and confirms qPCR as a reliable platform for the profiling of miRNAs in a diverse group of clinical samples [54], [55]. Furthermore, we validate the presence of exosomal miRNAs in cell-free patient plasma and mouse serum.

### Validation of exosomes and expression of exosomal markers

**Figure 2:**
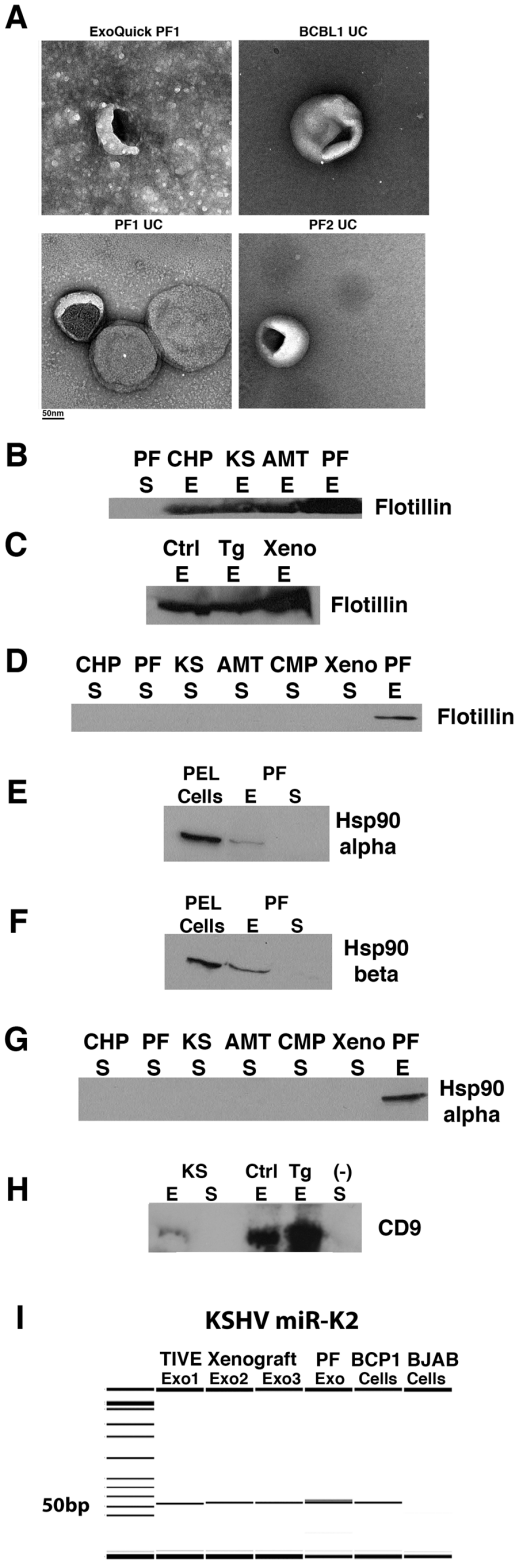
Characterization of patient- and mouse model-derived exosomes. (A) EM images of exosomes prepared from patient and tissue culture samples using Exoquick and ultracentrifugation (UC) methods. PF1, pleural fluid patient 1; PF2, pleural fluid patient 2; BCBL1 – PEL cell line. Scalebar is shown below images. (B–H) Abbreviations are as follows: CHP – Control, KSHV(−) Human Plasma, AMT – patients with non-KS AIDS malignancies, KS – Kaposi's Sarcoma patients, PF – Primary PEL Pleural Fluid, Ctrl – Control Mouse Serum, Tg – KSHV Latency Locus Transgenic Mouse Model, Xeno – TIVE-KSHV Xenograft Mouse Model, (−) KSHV-negative BJAB cell line. The exosomal markers flotillin-2 (B,C), Hsp90 alpha (E,G), Hsp90 beta (F) and CD9 (H) were analyzed by Western blot in human and mouse exosomes (abbreviated E) isolated using the Exoquick method. Exosome-depleted supernatants (abbreviated S) were also analyzed for the presence of Flotillin (B,D) and Hsp90 alpha (E,G). CD9 was detected in mouse exosome samples and exosomes from KS patients (KS), confirming our method of exosome isolation (H). As expected, the exosomal marker was absent in the supernatant fraction and in our negative control BJAB exosome-depleted supernatant fraction. Flotillin was present in exosomes derived from control (Ctrl), transgenic (Tg) and xenograft (Xeno) mouse models but was not present in the supernatant fraction. Hsp90 alpha and beta were expressed in PEL cells (VG1, a KSHV+ PEL cell line) and pleural fluid-derived exosomes (PF) but not in the supernatant. (I) KSHV miR-K2 expression was determined by qPCR and products were run on the Caliper LabChip GX. BCP1-KSHV (+) PEL cell line, Exo – RNA from exosome fraction, Cells – RNA from cell pellet. Exo1,2 and 3 denote three individual TIVE xenograft mice.

Isolation of exosomes using the Exoquick method has previously been validated to yield similar electron microscopy (EM) structures and miRNA array populations as other techniques [56]. Nonetheless, we sought to confirm the presence of exosomes in our patient samples using two independent isolation techniques. Enriched exosomes from the Exoquick protocol revealed similar structures via electron microscopy compared to exosomes enriched by differential ultracentrifugation (Figure 2A). However, while Exoquick samples did contain exosomes (determined by size and morphological characteristics), they yielded images with high background by electron microscopy due to the crowding agent present in the ExoQuick solution. This background was not due to contaminating cellular debris, as high-speed centrifugation and elimination of cellular debris using a sucrose cushion failed to eliminate background in the EM images (Figure S5). By comparison, differential ultracentrifugation yielded exosomes of similar size and morphology with minimal background (Figure 2A). Patient pleural fluid and BCBL1 cell supernatant yielded exosomes that appeared similar by EM. We therefore pursued the Exoquick method for further study, as these samples required much less sample input, a key benefit when working with clinical samples and mouse models.

To further establish the purity of our exosomes, we performed Western blots for previously established exosomal markers including the tetraspanin CD9, Hsp90 alpha/beta and flotillin [57], [58]. We first analyzed the expression of flotillin, which is enriched in exosomes [58], [59], [60]. Flotillin was expressed in all human and mouse exosome samples (Figure 2B,C) but was not present in the supernatant fractions containing freely circulating miRNAs (Figure 2D, S). Hsp90 alpha and beta, which are also highly enriched in exosomes, were detected in PEL cells and pleural fluid-derived exosomes but, as expected, were absent in the supernatant fraction (Figure 2E–G). Finally, we assessed the expression of the tetraspanin CD9, another exosomal marker. The KS exosomal subgroup (KS-E) expressed detectable levels of CD9 whereas the supernatant fraction (S) did not express the exosome marker (Figure 2H). As a negative control for exosomes, we used the exosome-depleted supernatant fraction from BJAB cells (-). The mouse exosome samples isolated from serum of control and transgenic (Tg) mice also showed robust expression of the CD9 exosome marker, indicating that these samples are enriched for exosomes (Figure 2H). The increased expression observed in the mouse samples most likely reflects the ratio of input used to the total fluid volume present in human and mouse. A mouse has a total blood volume of 1.5 mls of which we use 250 ul (∼17%) whereas human blood volume is approximately ∼5 L of which we used 250 ul (0.005%). We also tested the presence of exosomal markers in samples purified by ultracentrifugation and received similar results, validating the use of the Exoquick method for our study (Figure S6). Furthermore, the ExoQuick method yields more exosomes than other methods tested and uses approximately 100-fold less starting material.

### KSHV miRNAs are detectable in the exosome-enriched fraction

Increased expression of KSHV miRNAs correlates with disease state and tumor progression in endothelial cells [3], [6]. EBV viral miRNAs have been detected in exosomes isolated from cultured lymphoma cell lines, NPC patients and xenografted mice [44], [45], but thus far it has not been shown that KSHV miRNAs are also loaded into exosomes. To address this question, we examined patient-derived exosomes for the presence of KSHV-encoded miRNAs. Figure 2I shows the qPCR products separated by size on a Caliper nanofluidics platform. Exosomes derived from serum of three independent KSHV-positive TIVE L1 xenograft tumor mice and PEL fluid contained KSHV miR-K2 (Figure 2I). Total RNA from KSHV-positive latently-infected BCP-1 PEL cells were used as a positive control. KSHV miRs K12-4-5p, K12-4-3p, K12-5, K12-6-5p, K12-10a and K12-11 were also detected in pleural fluid and xenograft tumor mice (data not shown). KSHV miRNAs were undetectable in the KSHV-negative BJAB cell line (Figure 2I). This shows that systemically circulating exosomes contain appreciable levels of mature KSHV miRNAs and therefore exosomes containing KSHV miRNAs can travel from the subcutaneous tumor graft into the bloodstream and are stable enough to circulate systemically. Since we harvested the blood at day 10–15 after tumor cell injection, this result is likely to reflect steady-state levels of exosomal miRNAs. Notably, the L1 TIVE xenograft model does not generate infectious virus [48]. Hence, exosome encapsulated KSHV miRNAs show promise as a highly sensitive marker for latent KSHV tumor cells.

**Figure 3:**
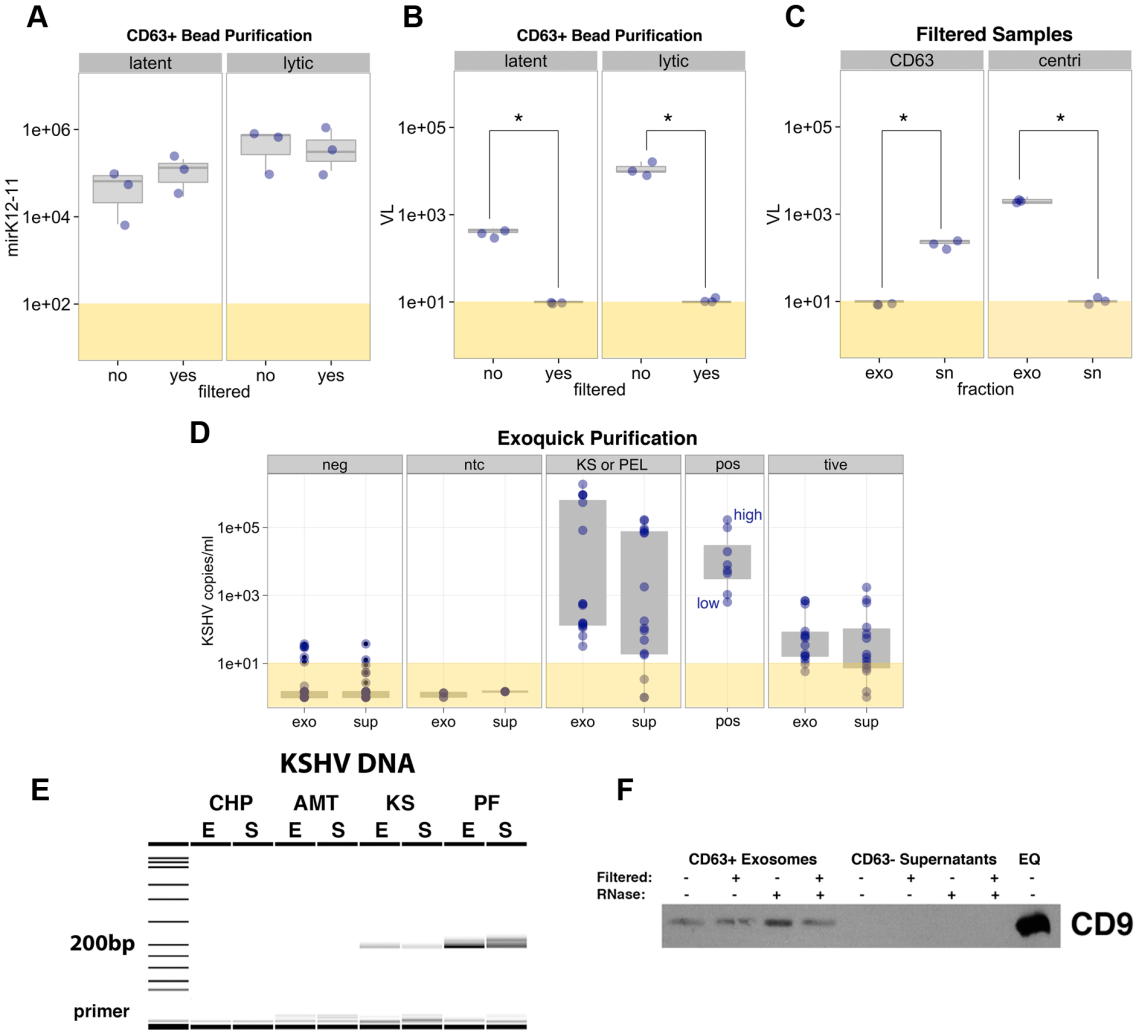
Analysis of KSHV miRNA expression and viral DNA in exosome samples. Box plot representation of miR-K12-11 expression (A) and KSHV load (B) in latent and lytic exosomes purified using the CD63+ Dynabeads method. Sample filtration is noted below each plot. MiRNA expression is shown as fold above background and viral load data is shown as copy number of LANA DNA per reaction. (C) Box plot of viral load for filtered samples purified using either CD63 (left panel) or differential ultracentrifugation (centri, right panel). Viral load is shown for both exosome (exo) and supernatant (sn) fractions. Asterisks denote significance of p≤0.05. (D, E) KSHV viral load from ExoQuick samples was determined by qPCR (D) and products were run on the Caliper LabChip GX (E). (D) Sample groups are as follows: neg (control human and mouse samples negative for KSHV), ntc (no template control), KS or PEL (KS patients and primary PEL fluid), pos (dilutions of oligonucleotide positive controls; high to low concentrations) and tive (xenograft mouse models of KS). (E) Sample abbreviations are as follows: KS = plasma from KS, AMT = AIDS Malignancy, non-KS, PF = pleural fluid, CHP = control human plasma, E = exosome fraction, S = exosome-depleted supernatant fraction. (F) Western blot for the exosomal marker CD9. Samples were enriched for exosomes using CD63+ Dynabeads and were filtered prior to bead purification as noted. Resulting CD63+ and CD63− fractions were treated with RNase as denoted and protein lysates were evaluated. As a positive control, a lysate of pleural fluid-derived exosomes using the ExoQuick (EQ) method were assessed for CD9 expression.

In order to study exosome-associated viral miRNAs in more detail, we used the BCBL1 PEL cell line to assess KSHV miRNA expression. We found that 14 out of 14 KSHV microRNAs tested were expressed at detectable levels in exosomes from latent BCBL1 cells (Figure S7). Methods for purifying virions and exosomes can lead to co-precipitating of both exosomes and virions, therefore making it difficult to physically separate them for analysis. To distinguish the source of these viral miRNAs as exosomal or virion-associated, we purified exosomes using three different techniques. In addition to using the ExoQuick method of purification, we also utilized differential ultracentrifugation and a new bead affinity purification technique that positively selects for CD63+ exosomes, an exosomal marker not present on KSHV virions. Expression of KSHV microRNAs was then assessed following enrichment of exosomes using either method (Figure 3, Figure S7). In addition, we passed the samples through a 0.2 µm filter prior to exosome isolation but after the removal of cellular debris. Although KSHV virions are approximately 180 nm in size, they tend to aggregate, a phenomenon well-recognized in earlier studies studying infectivity of cell-free virus [61], [62], [63]. This aggregation of virions makes it difficult to clear even a 0.2 µm filter. This was experimentally confirmed by filtering concentrated KSHV stocks, which resulted in a decrease in titer of approximately 4 logs (data not shown). Exosomes, however, which range in size from 30–100 nm, can easily pass through a 0.2 µm filter, as is evident from EM imaging of filtered patient-derived exosomes (Figure 2A). Consistent with this, the expression levels of KSHV miRNAs were only slightly affected by filtering (Figure 3A, Figure S7). By contrast, filtering of exosome samples resulted in a dramatic decrease in viral load (Figure 3B). We observed similar expression patterns of viral miRNAs in exosomes isolated and filtered following both ExoQuick and ultracentrifugation methods. This was confirmed by Caliper gel electrophoresis, which showed the presence of KSHV miRNA products in both the exosome and filtered exosome fractions (Figure S7). A lower shifted band corresponding to primer dimers was detected in the no template control reactions. Note also that the Caliper images represent non-quantitative accumulation of product after 55 cycles, whereas quantification was based on the exponential phase of the PCR reaction.

Using the CD63+ exosome isolation method, we consistently observed expression of KSHV miRNAs regardless of filtration (Figure 3A). The levels of viral miRNAs were not significantly different in exosome preparations from latent or lytically induced PEL cells (Figure 3A). If these miRNAs were predominantly present within virions, we would expect a robust increase in viral miRNA levels concomitantly with increased virion production following reactivation as we observed for KSHV load (Figure 3B). Furthermore, RNase treatment of samples slightly decreased viral miRNA levels in the CD63− supernatant but did not affect KSHV miRNA expression in CD63+ fractions, suggesting that these miRNAs are primarily protected within exosomes (data not shown). Analysis of exosomes isolated by CD63 affinity capture confirmed the presence of CD9, another well established exosomal marker (Figure 3F). CD9 levels were unaffected by filtering samples and RNase treatment (Figure 3F). Taken together, this demonstrates that the KSHV miRNAs are predominantly contained within exosomes released from latently-infected tumor cells.

### Analysis of viral load in exosome-enriched samples

Having determined that viral miRNAs were present in exosomes, we next sought to analyze the distribution of KSHV DNA among our samples and biochemical fractions. There are two mechanisms that lead to KSHV viral DNA being detectable in body fluids: (i) virions [64], (ii) tumor cell-released free viral DNA, as has been demonstrated for EBV [65], [66], [67], [68]. To eliminate the contribution of cell-free viral DNA, we treated all samples with DNase prior to DNA isolation. We evaluated BCBL1-derived exosomes purified using different techniques for the presence of KSHV DNA (Figure 3B, C). Exosome-enriched samples were passed through a 0.2 µm filter, which led to a drastic decrease in KSHV load using both purified virus stock and exosomes (Figure 3B, data not shown). Although the viral load increased following reactivation, filtering of exosomes from lytic BCBL1 cells abolished viral load to approximately the limit of detection. DNase treatment of samples, which effectively eliminated freely circulating tumor-associated DNA, further decreased KSHV load in filtered fractions (data not shown).

We also compared the presence of viral DNA in exosome and supernatant fractions of samples enriched by CD63 bead affinity purification or differential centrifugation (Figure 3C). Viral DNA was detected in the exosome-depleted supernatant fraction (CD63−) after bead affinity purification but was undetectable in the CD63+ exosome fraction. Conversely, viral DNA was enriched in the exosome pellet following differential ultracentrifugation, as both virions and exosomes sediment at similar densities during centrifugation. This establishes CD63-based affinity capture as an efficient way to separate exosomes and virions. Since we detected KSHV miRNAs, but not KSHV DNA in the CD63-affinity purified exosomes, this suggests that the primary source of the viral miRNAs we observe is exosomes rather than virions.

We also evaluated viral load in exosomes purified using the ExoQuick method. The advantage of the ExoQuick method compared to CD63 capture is greater efficiency (using only 250 µl as input), which is essential when profiling large numbers of clinical samples. We found KSHV DNA in both exosomal and free supernatant fractions of plasma and pleural fluid (Figure 3D,E). The highest viral load was found in exosomes derived from PEL pleural fluid. No viral DNA was detected in our negative control samples or in exosomes purified from non-KS, HIV+ patients (Figure 3D, 3E lanes CHP and AMT respectively). Both ExoQuick and ultracentrifugation methods yielded KSHV DNA in the KS patient plasma and PEL fluid exosome fraction (Figure 3). Thus, neither differential centrifugation not ExoQuick can with certainty be used to separate exosomes from virion particles. However, CD63+ exosomes contain little KSHV DNA, especially following filtering of exosome samples. This novel method confirms that the majority of the signal detected in the viral load assay was due to free DNA and KSHV virion DNA.

### KSHV protein and virions are not detected in exosome-enriched samples

To further address the possibility that virions may also be present in our exosome fraction and may contribute to our results, we looked for KSHV virions and viral proteins in our exosome-enriched samples. We did not detect any virions by EM following ExoQuick or ultracentrifugation isolation of exosomes (Figure 2A). We analyzed more than 20 grids for the presence of virions in our exosome-enriched samples. Quantitative analysis of exosome-enriched pellets by differential centrifugation revealed 2,319 exosomes and no virions on three sample grids. The supernatant fraction was also imaged by EM for the presence of exosomes and only 13 exosomes were detected, validating ultracentrifugation as an efficient method for exosome isolation. We also compared the number of exosomes from latent and lytic BCBL1 cell supernatants by EM. Both latent and lytic samples had similar numbers of exosomes detected on representative grids, averaging 135 and 126, respectively (while the DNase resistant viral load differed by >10-fold). This suggests that the presence of exosomes in our samples may be static and independent of virus production.

Finally, we probed exosome-enriched samples for KSHV structural proteins. KSHV K8.1 was readily detected in BCBL1 PEL cells following lytic reactivation (Figure S8). However, PF-derived exosomes, which contained the highest viral load of our samples, did not express K8.1. These data confirm that our exosome-enriched samples do not contain appreciable levels of KSHV virions (Figures S7, S8). Although KSHV protein and DNA are not found in exosomes, we find systemically circulating KSHV miRNAs in exosomes derived from patients, tissue culture models and mouse models of KS (Figure 2I, Figures S7, S8). This establishes exosome-associated viral miRNAs as new biomarkers for KSHV-associated cancers. It also suggests that detecting viral miRNAs may offer greater sensitivity of diagnosing viral infection than viral load measurements.

### miRNA profiling reveals distinct oncomiR and exosome signatures

**Figure 4:**
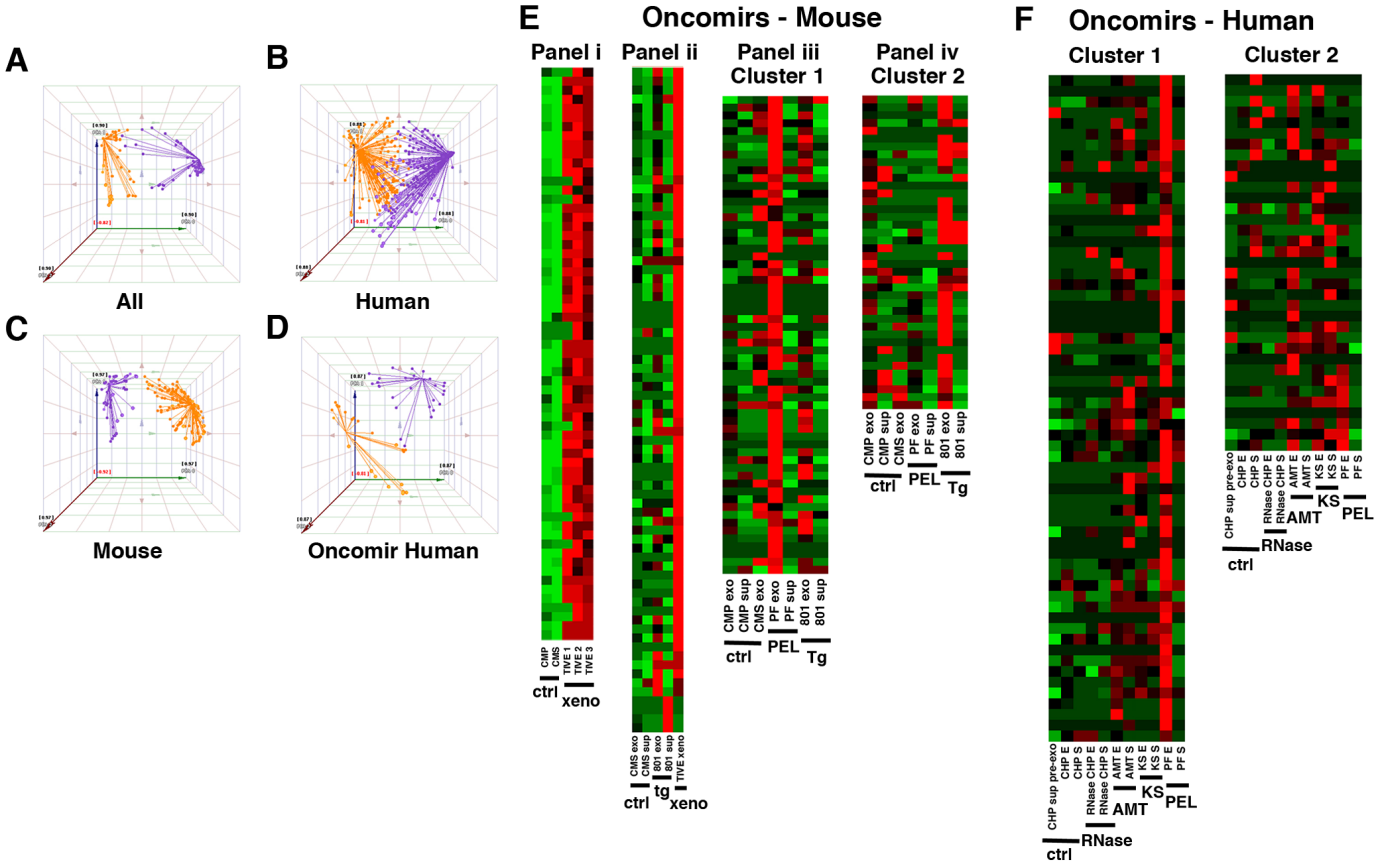
miRNA profiling reveals distinct oncomiR and exosome subsets. Profiling of miRNAs led to the discovery of distinct signatures for oncomiRs and exosome subsets. Cluster separation of miRNA expression by principal component analysis (PCA) of (A) all miRNAs profiled, (B) human samples, (C) mouse samples and (D) oncogenic miRNAs in human samples. MiRNAs were clustered by their levels of expression to reveal two distinct clusters of expression patterns: one with generally high expression in KS and PEL samples (purple, cluster 1) and one with high expression in another subset of samples specific to a control, malignancy or exosome-specific (shown in yellow, cluster 2). Each solid circle represents one microRNA and lines are drawn from each point to the centroid or mean position of points in a given cluster. This centralized point allows for the largest difference between clusters and minimizes the distance of points within a given cluster to the centroid. Heatmaps reflective of unsupervised clustering analysis are shown for oncomirs in (E) mouse and (F) human samples. Enlarged heatmaps with microRNA labels are shown separately as Figures S10, S11. (E) Oncomirs from mouse models are shown as a series of panels (i–iv). Panel i compares oncogenic miRNA expression between control mice and individual TIVE xenograft mice. Profiling data from the transgenic mouse model encoding the KSHV latency locus is shown in Panel ii with control mice and the average of the TIVE xenograft data. Panels iii and iv show two separate clusters of miRNA expression and compare control and transgenic mice to the primary human PEL pleural fluid cases. Abbreviations of mouse samples are as follows: CMP – control mouse plasma, CMS – control mouse serum, TIVE 1–3, individual TIVE xenograft mice, tg – 801 latency locus mouse model, PF – primary human PEL pleural fluid, exo – exosomes, sup – exosome-depleted supernatants. (F) Oncomir expression in human samples is shown as two separate clusters of expression. For the human oncomiR heatmap, sample lanes from left to right are: CHP sup pre-exo - control human plasma supernatant pre-ExoQuick, CHP E – control human plasma exosomes, CHP S – control human plasma exosome-depleted supernatant, RNase CHP E – RNase-treated CHP exo, RNase CHP S – RNase-treated CHP sup, AMT E – AIDS Malignancy, non-KS exo, AMT S – AIDS Malignancy, non-KS sup, KS E – Kaposi's sarcoma exosomes, KS S – Kaposi's sarcoma exosome-depleted supernatant, PF E – pleural fluid exosomes, PF S – pleural fluid exosome-depleted supernatant. Red denotes high expression, green denotes low expression and black is basal or intermediate expression.

To obtain a more complete picture, we profiled the host miRNA repertoire in each of our sample groups using both exosomal and exosome-depleted supernatant preparations. The *C. elegans* cel-mir-39 spike-in was used as an internal normalizing control. Unsupervised clustering analysis revealed two distinct clusters, which are shown as projected onto the first three principal components (Figure 4A). Unsupervised clustering groups samples and the different miRNAs based on similar expression levels. The result is typically shown as a heatmap. Principal component analysis is used to reduce the complexity of the data further without loss of statistical power. It combines the multiple measurements of each sample (or each miRNA) to such that the data can be represented in three dimensions (the principal component axes). Individual analysis of the human and mouse profiling samples (Figures 4B,C) illustrates the more divergent clusters representing miRNAs elevated in tumor versus control samples in the mouse model. We expected to see more defined clusters in our mouse models since the xenografts represent biological replicates with limited variability compared with human clinical samples. The miRNA profile in the human samples alone clearly separated samples into KSHV-associated and control groups.

When we further narrowed the miRNAs to known oncomiRs and tumor suppressor miRNAs, the classification improved (Figure 4D). A list of these ∼150 oncomirs and tumor suppressor miRNAs is shown in Table S2. As a negative control for our analysis we clustered an unrelated sample. We performed unsupervised clustering of miRNAs in HEK293 cells following infection with West Nile Virus (WNV) (Chugh and Dittmer, unpublished data). This comparison yielded very different clusters of miRNAs compared with the KSHV exosome data as noted by further predicted target analysis (Table 1). This establishes a unique oncomir signature of KS- and PEL-associated exosomes.

**Table 1:** GO Pathway analysis of induced oncomiR targets^a^.

	KSHV		WNV		Migration^b^
Pathway	No.^c^	P value^d^	No.	P value	
Pathways in Cancer*	18	8.35 E-05	18	0.09	X
Adipocytokine signaling	8	2.08E-04	NA	NA	
Pancreatic Cancer*	8	3.26E-04	NA	NA	
MAPK signaling*	15	3.39E-04	17	0.04	X
Adherens junction*	7	2.80E-03	NA	NA	X
TGF-beta signaling*	7	5.15E-03	NA	NA	X
Fc gamma R phagocytosis	7	7.89E-03	NA	NA	
Focal adhesion*	9	3.15E-02	NA	NA	X
TLR signaling*	6	3.83E-02	NA	NA	
Wnt signaling*	7	5.97E-02	12	0.02	X
Colorectal cancer	5	6.91E-02	7	0.09	
NSCLC^e^	4	7.73E-02	NA	NA	
AML^f^	4	9.13E-02	NA	NA	

a The oncomiRs that were upregulated in the KSHV-associated sample groups were input into a microRNA target prediction database (MAMI). The predicted targets (determined with highest stringency) were used as input for the GO pathway database DAVID and the KEGG pathway terms are listed above. Asterisks denote pathways that have been previously known to be modulated by KSHV.

b Pathways involved in cell migration.

c The number of predicted microRNA target genes involved in each pathway.

d P value. In addition to the predicted targets of the KSHV oncomirs, also shown are predicted targets of WNV-induced microRNAs, demonstrating the differences in pathways affected.

e NSCLC, non-small cell lung cancer.

f AML, acute myeloid leukemia.

We further examined the expression of individual oncomirs and tumor suppressor miRNAs in the mouse exosome subset by heatmap analysis (Figure 4E). Oncomirs were defined as host miRNAs readily studied for their role in tumorigenesis and related cancer signaling pathways while tumor suppressor miRNAs have been demonstrated to functionally inhibit these processes (Table S2). We identified this subset of oncogenic miRNAs because (a) we previously extensively validated these assays [3], [4], [55] and (b) they represent miRNAs with experimentally verified expression and function. The most distinct expression pattern was the apparent separation between TIVE xenograft and control mouse serum (Figure 4E, Panel i). The majority of oncomiRs in this cluster were increased in exosomes from KS xenograft tumor models and were only minimally or not detectable in the control mice (ctrl versus xeno). We next compared miRNA expression profiles of control and xenograft mice to our latency locus transgenic mouse model (Figure 4E, Panel ii). In this novel model, only the KSHV latent genes and miRNAs are expressed in B cells [50]. However, none of the viral structural genes are present. We found that exosomes derived from the transgenic model differed from that of control mice and shared some oncogenic miRNA expression with the xenograft mice. As this transgenic mouse model phenotype represents B cell hyperplasia, these highly expressed miRNAs may be reflective of change in the miRNome regulated by the KSHV latency locus prior to tumor formation (Figure 4E).

We further compared the exosomal miRNA profile of transgenic mice to that of PEL-associated exosomes from primary pleural fluid (Figure 4E, panels iii, iv). This yielded similarities in induced exosomal oncogenic miRNA expression between the 801 transgenic mouse model and PEL patient fluid (Panel iii, Cluster 1). Interestingly, we also identified a subset of microRNAs that was solely induced in the KSHV latency locus transgenic mouse model (Figure 4E, Panel iv, Cluster 2). Figure S9 further compares the exosomal miRNA profile in an independent set of transgenic and control mice and indicates elevated levels of oncogenic miRNA expression in the transgenic mouse model.

Analysis of miRNA profiles in both Clusters 1 and 2 also revealed a subset of oncogenic miRNAs that were exclusively expressed in exosomes, suggesting that these miRNAs may be preferentially incorporated from the tumor site into exosomes for intercellular communication (Figure 4E, Panels iii,iv, Figure S10). Taken together, we find that the most elevated oncomiR levels in exosomes were observed in the TIVE xenograft tumor group, as these mice were bearing large, well-vascularized tumors, which facilitates expression and release of miRNAs. This demonstrates for the first time that exosomal miRNAs, including KSHV miRNAs, can be detected in mouse models of KS.

Our human clinical samples of AIDS-KS recapitulated the trends in oncogenic miRNA expression observed in our mouse models (Figure 4F, Figure S11). Cluster 1 represents a subset of oncogenic miRNAs that are most highly expressed in exosomes derived from PEL pleural fluid (Figure 4F). Several miRNAs in this cluster were also elevated in KS patient-derived exosomes. This pattern of miRNA expression may reflect a signature of KSHV-associated malignancies. A subset of miRNAs within this cluster could also represent miRNAs overexpressed in KS and other cancers since we observed oncogenic miRNA induction in other AIDS malignancies as well as KS-associated exosomes (Figure 4F). Cluster 2 shows another subset of miRNAs with elevated expression in exosomes from KS or AIDS malignancy patients. This cluster also includes several miRNAs that seem to be preferentially expressed within exosomes compared to the supernatant fraction.

We noticed little difference in the miRNA profile from control plasma exosomes versus RNase-treated control plasma exosomes, indicating that exosomes are indeed resistant to RNase treatment [35](Figure 4F, lanes CHP exo and RNase-CHP exo). We also compared the miRNA profile in pleural fluid-derived exosomes exposed to RNase to determine if they responded similarly to our exosomes from control human plasma. Exosomes from PEL patient pleural fluid exhibited higher levels of miRNA expression. RNase treatment only slightly changed the miRNA profile, similar to that observed in control exosomes (Figure S12). This demonstrates that different patient samples respond similarly to RNase treatment and further validates that the majority of our signal was derived from exosome-contained miRNAs.

Since patient samples may display a high degree of genetic variability and therefore miRNA signatures could differ, we sought to address the issue of individual variance of patient miRNA profiles using three PEL patients. Pleural fluid-derived exosomes were independently isolated and the miRNA expression profile was compared to that of control human exosomes. Many of the “PEL signature” miRNAs were expressed in all three patients, suggesting that these could be used as novel biomarkers of PEL present in pleural fluid (Figure S13). Another subset of miRNAs was expressed in 2 out of 3 patients. Since PEL is a rare malignancy, we obtained only three patients, each of varying disease states. Different factors such as disease state, co-infection with EBV or HIV status could contribute to absence of these biomarkers in one of the patients. However, despite the inherent genetic variability among patients, we could identify multiple miRNAs that were expressed at high levels in all three PEL patients compared to controls (Figure S13).

### Analysis of oncomiRs, the miR-17-92 cluster miRNAs and exosomal miRNA subsets

**Figure 5:**
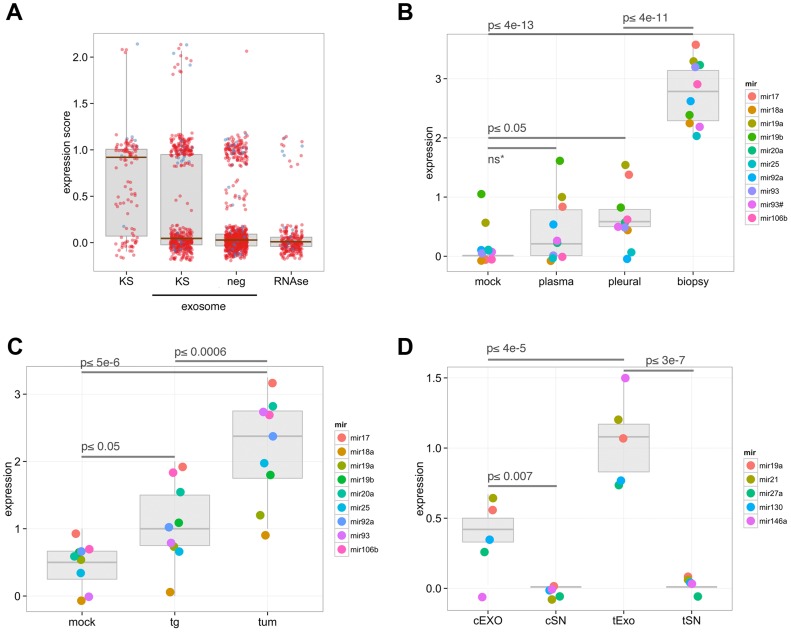
Analysis of oncomiRs and exosomal miRNA subsets. Oncomirs belonging to the miR-17-92 cluster were analyzed for expression. (A) Box plot showing the distribution of expression scores for ∼150 miRNAs associated with cancer for KS biopsies, exosomes in KS patients, exosomes in normal human plasma and RNase-treated, exosome-depleted supernatant from control plasma. Individual circles represent individual miRNAs and their respective expression levels in the samples. Blue circles represent members of the oncogenic miR-17-92 cluster while other oncogenic miRNAs are denoted by red circles. Box plots of relative expression levels of the miR-17-92 and 106b-25 clusters in control and tumor samples are shown in human (B) and mouse (C) exosome samples. (D) A subset of miRNAs showed exclusive expression in mouse exosomes and not in plasma exosomal supernatants (free miRNAs). Expression levels in transgenic and xenograft mouse exosomes are also higher than control exosomes for these miRNAs. Sample abbreviations: exo, KS exosomes; neg, control exosomes; tumor, tumor biopsies; mock, control human or mouse plasma/serum, plasma, KS patient plasma; pleural, pleural fluid; tg, KSHV latency locus transgenic mice; tum, xenograft tumor mice; cEXO, control exosomes; cSN, control supernatant; tExo, tumor exosomes; tSN, tumor supernatant fraction.

We further analyzed the expression of the oncomiR cluster in the exosome sample subsets. For this analysis, we defined a relative expression score based on the CT where a higher expression score corresponds to a lower CT. Specifically, we calculate the expression class by binning CTs such that a CT of 20–25 corresponds to an expression value of 3. MicroRNAs expressed with CTs of 25–30 are assigned an expression value of 2.5. Scores are assigned in 0.5 increments until CT of 45+ equals zero, or not detected. In addition to patient-derived exosomes, we determined the miRNA profile for KS biopsies and PBMCs derived from control human plasma. The full profiling data is shown in Figure S14. Figure 5A demonstrates that, as expected, KS biopsies (KS, left) displayed the highest expression of oncomirs. By comparison, a large number of oncomirs were undetectable (expression score = 0) in biofluids. KS-associated exosomes also contained oncomirs in moderate (expression score = 1) and some at very high levels (expression score = 2). While oncomiRs are readily expressed in both control and malignant samples, we found that the number of highly expressed oncogenic miRNAs was lower in control exosomes (Neg). Note that members of the miR-17–92 cluster are denoted by blue dots and are highly expressed in KS biopsies and KS-associated exosomes compared with controls (expression score>1.5). The levels of oncogenic miRNAs were abolished in exosome-depleted supernatant fractions treated with RNase (Figure 5A). The exceptions were miRNAs including miR-16, miR-195 and miR-197, which were previously shown to be RNase-resistant (Figure 1B, [35]). This demonstrates that most oncogenic miRNAs were present in exosomes. Oncomirs specifically expressed in tumor samples at the highest expression level included miR-106a, miR-17, miR-454, let-7e, miR-451, miR-886-5p, miR-601 and miR-625 (expression score of ≥2, Figure 5A). Note, that a high expression score is the result of both the underlying high level of expression of the specific miRNA species and the sensitivity of the particular qPCR assay.

One of the most well-studied oncogenic miRNA clusters is the miR-17-92 cluster. The 6 mature miRNA species in this cluster tend to be co-regulated [69] and we previously found this miR cluster upregulated in KS [3], [4]. Members of the paralog cluster miR-106b/25 are also well-known for their role in tumorigenesis and share target genes with the miR-17-92 cluster [70], [71]. We therefore investigated whether KS-associated exosomes contained members of these two miRNA clusters. In our clinical and mouse model samples, levels of the miR-17-92 and miR-106b/25 clusters were induced in exosomes derived from KSHV-associated mouse serum, primary human pleural fluid and KS biopsies compared with control exosomes (Figure 5B, C). Since we did not observe a similar enrichment of all tumor-associated miRNAs within the exosomes, these miR-17-92 members are likely to be preferentially incorporated into exosomes. Members of these oncogenic clusters were slightly elevated in exosomes from KS patient plasma, although this was not statistically significant (Figure 5B). However, exosomes derived from pleural fluid expressed much higher levels of the miR-17-92 and miR-106b-25 cluster members, with the exception of miR-25 and miR-92a (Figure 5B). The increased expression of oncogenic miRNAs within PF-derived exosomes may be because of direct contact of the pleural fluid to PEL cells, suggesting that malignant effusions may be a very effective source for obtaining exosomes (Figure 5B). Induction of the miR-17-92 cluster member miRNAs was most pronounced when we compared exosomes derived from the xenograft mouse model to control mouse exosomes (Figure 5C, p≤.000059). Therefore, we find that exosome-associated oncomirs are uniquely upregulated in samples from KS tumor-bearing animals and primary PEL patients. Interestingly, even the B cell hyperplasia latency locus transgenic mouse model showed increased levels of these miRNAs in systemically circulating exosomes (Figure 5C, p≤0.05).

Several miRNAs seemed to be preferentially incorporated into exosomes (Figure 4E,F). Therefore, we analyzed these in detail. As shown in Figure 5D, miRNAs miR-19a, miR-21, miR-27a, miR-130 and miR-146a were enriched within exosomes and virtually undetectable as free, circulating miRNAs in the supernatant. Their relative expression levels were significantly elevated in mouse models of KS (p≤4×10^−5^, Figure 5D). To confirm these results, we performed Caliper gel electrophoresis analysis on the qPCR products, which confirmed that these miRNAs were overexpressed in exosomes from our transgenic and TIVE xenograft mouse models (Figure S15). Taken together, these data reveal that members of the miR-17-92 cluster are exclusively incorporated into exosomes and may exhibit diagnostic potential and contribute to tumor development and pathogenesis of malignancies such as KS.

### Profiling of miRNAs from KS case studies and comparison to TIVE xenograft mouse models

We profiled the circulating miRNAs in a second, independent pair of KS patients (n = 2) and compared them to TIVE xenograft mice along with the appropriate controls (Figure S16). One of the KS patients profiled had an unusually high KSHV load, multiple internal lesions and cytokine dysregulation [72]. Unsupervised clustering analysis confirmed that the TIVE L1 xenograft mice had a distinct circulating miRNA profile from control mice, but also revealed that this mouse model shared similarities to the human miRNome detected in pleural fluid (Figure S16). Like the xenograft mice, the two KS case study patients expressed distinct circulating miRNA signatures when compared with control human plasma (Figure S16). The KS patient with more advanced disease (DG1, cytokine dysregulation and high KSHV load) displayed a miRNA profile more similar to TIVE xenograft exosomes. These independent biological replicates and multiple clinical cases share a common, robust signature (Figure S16, Figure 4E,F).

### GO pathway analysis of oncomir targets

To gauge the importance of the KS exosome signatures, we analyzed the oncogenic miRNAs upregulated in tumor-derived exosomes using Gene Ontology pathway analysis and found that many of the miRNAs targeted pathways previously shown to be central to KSHV pathogenesis (Table 1; asterisks). PI3K/Akt signaling is central to “Pathways in Cancer”, which had the highest correlation to upregulated miRNAs. It is known to be dysregulated following KSHV infection [73], [74], [75]. Many of the other pathways listed in Table 1 contribute to both the KEGG Pathways in Cancer and the Pancreatic Cancer pathway. For instance, MAPK is important in the control of replication and KSHV reactivation from latency while KSHV inhibits TGF-beta signaling through mechanisms including miRNA-targeted silencing [76], [77]. KSHV LANA has been shown to bind GSK3-beta, leading to an upregulation of beta catenin in KS and PEL through the regulation of Wnt signaling [78]. TLR signaling has previously been shown to play a role in both primary infection of monocytes and reactivation from latency [79], [80]. Finally, two of the pathway hits—focal adhesion and adherens junctions—are known to be important in viral entry, cytoskeletal remodeling and cell adhesion during KSHV infection and KS tumorigenesis including in adjacent KSHV-negative spindle cells within the KS lesion [81].

As control, we also analyzed the GO pathways associated with the miRNA signature of an unrelated virus (WNV). This confirmed that the roles of the signaling pathways were unique to our exosome profiling of KSHV-associated malignancies (Table 1). We also performed GO pathway analysis using two additional, independent analysis databases: Panther and Ingenuity Pathway Analysis (Table S4 and S5). These revealed highly significant pathways targeted by oncomirs including angiogenesis, integrin signaling, transformation, migration and invasion (Tables S4, 5). Previous studies of the oncogenic miR-17-92 cluster have also revealed roles in similar pathways such as NFkB signaling, angiogenesis, TLR, MAPK, STAT and TGF-beta signaling [69], [82], [83], [84], [85], [86], [87], [88], [89], [90], [91]. Since many of the GO analysis pathway hits have been previously functionally validated, it is likely that some of the exosomal miRNAs found overexpressed in this study contribute to KSHV signaling. One function that many of these pathways shared is the involvement in cell migration, which is important for tumorigenesis and noted in Table 1. We therefore used cell migration as a bioassay to show that our exosome-enriched samples yielded intact, functional exosomes.

### Treatment of hTERT-HUVECs with patient-derived exosomes enhances cell migration

**Figure 6:**
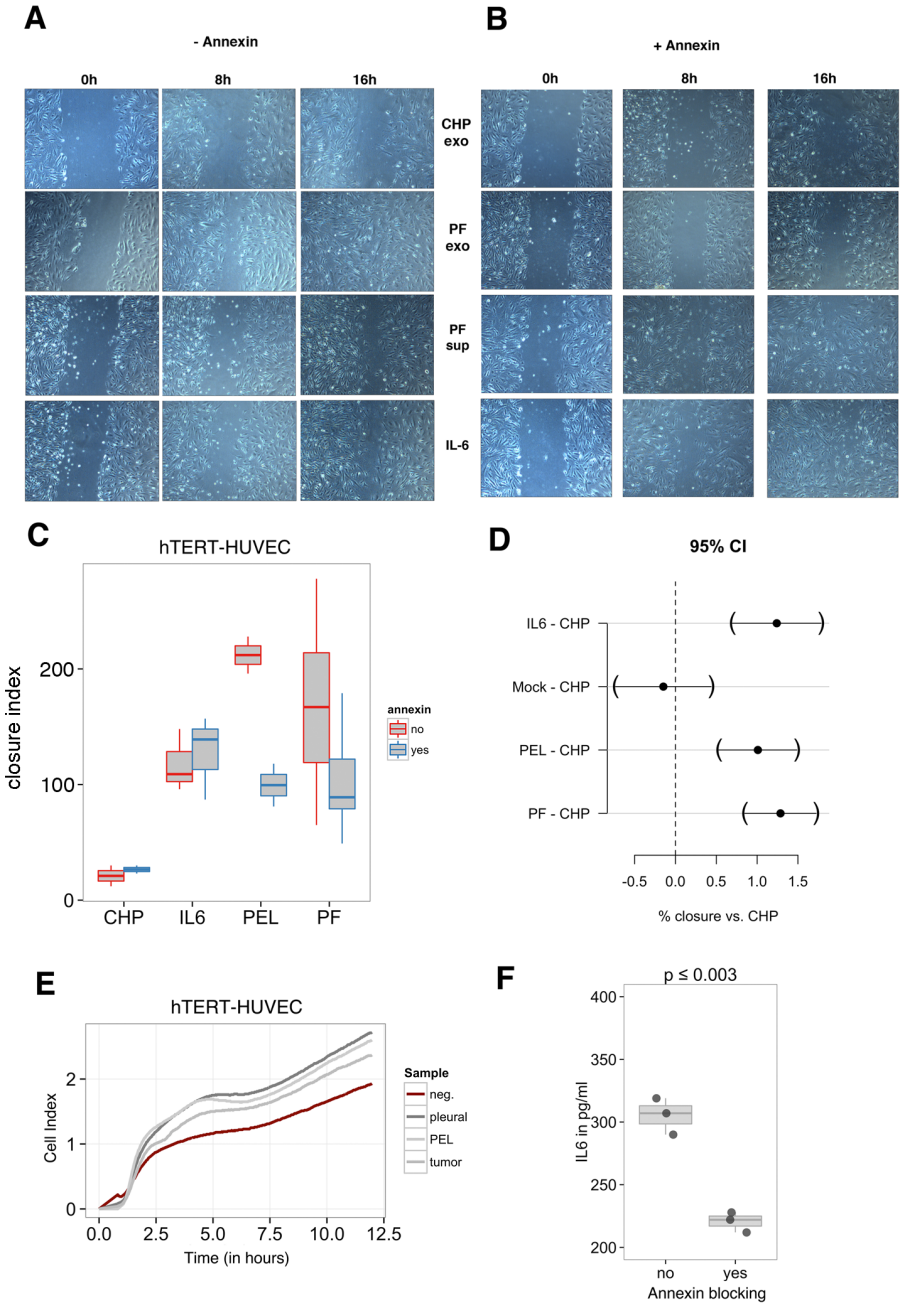
hTERT-HUVEC cell migration is enhanced upon treatment of cells with patient-derived exosomes. (A) hTERT-immortalized HUVECs were seeded at 80% confluence in a 24-well plate and allowed to equilibrate overnight. Cells were then treated with patient pleural fluid-derived exosomes for 24 hours prior to beginning the scratch assay. (B) Scratch assay performed with annexin blocking of exosomes. Scratch assay images are shown at 0 h, 8 h and 16 h post-scratch at 100× magnification. CHP – control human plasma; PF Exo – pleural fluid exosomes; PF Sup – exosome-depleted pleural fluid supernatant; IL-6 – interleukin 6. (C) Box plots of scratch assay data. Cells treated with exosomes only are shown in red with the horizontal bar representing the mean of experiments for each group. Data for annexin blocking of exosomes is shown in blue. The closure index represents the amount of closure detected at 8 hours post-scratch for samples as compared to mock. (D) Dunnett confidence interval (CI) comparing each treatment to control human exosomes. Black circles represent the 95% CI for each sample, with parentheses denoting the range observed. Dotted grey line represents CHP compared to CHP as a baseline comparison. (E) Migration assay using the xCelligence system. hTERT-HUVECs were treated with exosomes for 24 hours and serum-starved for 6 hours. 30,000 cells were plated per well of an xCelligence CIM-Plate 16 (upper chamber) and FBS was used as a chemoattractant (lower chamber). Reads were taken every 2 minutes continuously for 24 hours. Data is shown as Cell Index and increased cell index reflects increased migration to the lower chamber. (F) Supernatants from the scratch assay (A, B) were assessed for levels of IL-6 (pg/ml) by ELISA. Box plots show replicates for supernatants of hTERT-HUVECs treated with pleural fluid-derived exosomes before and after annexin blocking.

Since cell migration was a shared functional outcome of several of the gene ontology pathway hits, we sought to test the effect of KS and PEL-derived exosomes on the migration of endothelial cells. hTERT-immortalized HUVECs [92] were treated with exosomes isolated using the ExoQuick kit. Exosomes derived from patient PEL pleural fluid were added to cells for 24 hours and the wound healing scratch assay was performed to test the migration capability of these cells. Figure 6A demonstrates that hTERT-HUVECs treated with patient-derived exosomes displayed enhanced cell migration by 8 hours post-initiation of the scratch assay. Cells treated with exosomes derived from control human plasma (CHP) showed delayed migration compared with cells receiving the exosomes derived from pleural fluid (Figure 6A). This confirms that this effect was not due to ExoQuick itself, since control exosomes isolated using this protocol did not increase migration. Since we also detected KSHV DNA in the supernatant fraction of pleural fluid and the presence of virions can also affect migration, we analyzed the migration capability of cells treated with exosome-depleted supernatant (PF sup). hTERT-HUVECs exposed to PF supernatant also displayed enhanced migration compared to control exosomes but cells treated with pleural fluid-derived exosomes still migrated more rapidly. As a positive control, we also treated hTERT-HUVECs with IL-6, which resulted in increased migration similar to that observed with the pleural fluid supernatant fraction (IL-6 versus PF sup). Of note, exosomes are known to carry proteins as well as miRNAs [44]. At this point, we cannot assign this exosome phenotype to either moiety. The data also suggests that while cytokines and virus present in the supernatant can affect cell migration, patient-derived exosomes further accelerate this process.

Exosomes isolated from cell culture models and patients have been shown to express phosphatidylserine (PS) on their surface [39], [93]. Since Annexin V can bind PS on the surface, annexin blocking of exosomes has been previously used as a means of inhibiting exosome fusion and transfer of exosomal contents [44], [45], [93]. Therefore, we also performed the scratch assay in the presence of annexin blocking (Figure 6B). Exosomes and supernatants were incubated with Annexin prior to initiation of the scratch assay. Annexin blocking did not seem to affect the migration of hTERT-HUVECs treated with control (CHP) exosomes. However, the enhanced migration potential of cells treated with pleural fluid-derived exosomes was reversed with annexin blocking, demonstrating that this phenotype is due to exosomal transfer. Cells treated with exosome-depleted supernatants from pleural fluid were not affected by annexin blocking. Similarly, IL-6 enhanced cell migration regardless of annexin blocking. Therefore, any virus or cytokines present in this supernatant enhanced migration via a different mechanism independent of exosomes.

Figure 6C provides a boxplot representation of the scratch assay data. This confirms that cells treated with pleural fluid-derived exosomes exhibit increased migration, which is reversed by treatment with annexin. This is also observed following treatment of cells with exosomes derived from the PEL cell line BCBL1. We formally tested the individual contributions of each factor to the increased migration phenotype using a Dunnett confidence interval test which evaluates the significance of different treatments compared to a common control and adjusts for potential bias due to multiple comparisons being performed (Figure 6D). As represented by the black circles (with brackets representing the 95% confidence interval (CI)), treatment with IL-6 or exosomes from either pleural fluid or PEL cells independently led to significantly increased closure of the wound compared to exosomes isolated from KSHV-negative control human plasma (CHP). By contrast exosome-free, mock treated cells behaved similarly to cells treated with exosomes from KSHV-negative CHP.

All scratch assays were performed in triplicate for three independent biological replicates over a span of two weeks. In each biological replicate, we observed the same phenotype. Table 2 shows the linear, multivariate analysis of the data, which measures the difference between two experimental conditions after adjusting for all other factors. Exosomes derived from pleural fluid of a PEL patient (p≤10^−11^) or from the BCBL1 PEL cell line (p≤10^−7^) significantly enhanced migration of hTERT-HUVECs at 8 hours post-infection compared to CHP (Table 2b–d). When comparing the supernatant and exosome fractions of pleural fluid and PEL cell supernatants, exosomes were more potent (p≤0.031), but we still observed a significant effect on HUVEC migration for the supernatant (Table 2j). This is not entirely unexpected, since supernatants from PEL patients and PEL cells have large amounts of soluble IL-6, IL-10 and VEGF [49]. Still exosomes independently confer an enhanced migration phenotype to hTERT-HUVECs. Annexin blocking of exosome fusion supports this (p≤10^−7^) and resulted in reversal of the enhanced migration effect of PEL-derived exosomes (Table 2k, columns b–d). This demonstrates that our purified exosomes have biological activity, and second that the KS and PEL patient-derived exosomes confer a phenotype of enhanced migration to endothelial cells, which is likely to contribute to KS-associated angiogenesis.

**Table 2:** Linear, multivariate analysis of scratch assays^a^.

	Estimate^b^	SEM^c^	p-value^d^
**(Intercept)** e	0.31	0.23	n.s.^f^
**Experiments** g	0.03	0.022	n.s.
**CHP** h **vs. IL6** i	1.2	0.22	1.7×10^−07^
**vs. mock**	0.0	0.25	n.s.
**vs. PEL cell (BCBL1)**	1.1	0.20	4.0×10^−07^
**vs. PEL patient (PF)**	1.3	0.18	5.4×10^−11^
**SN** j **vs. Exosome**	−0.31	0.14	0.031
**Mock vs. AnnexinV** k	−0.75	0.14	3.3×10^−07^

a Total number of assays n = 94.

b Estimates relative effect of variable on fraction of closed area of the scratch after 8 hours relative to mock treatment. A negative coefficient indicates inhibition relative to control.

c SEM, standard error of the mean.

d Unadjusted p-value of F test for significance (p≤0.05 is considered significant.

e Intercept term of the linear model.

f n.s., not statistically significant.

g Total number of independent experiments n = 9.

h CHP, control human plasma.

i Human IL6.

j SN, supernatant fraction after exo quick kit.

k Presence of Annexin V, which prevents exosome fusion.

We next analyzed migration of hTERT-HUVECs treated with exosomes using the xCelligence system, which allows for highly accurate, quantitative measurements of cell migration in real-time. The xCelligence Cell Invasion and Migration (CIM) Plate 16 consists of an upper and lower chamber separated by a microporous membrane coated with gold microelectrode sensors on the bottom side. As cells migrate toward the chemoattractant in the bottom chamber, the impedance signal increases and results in a corresponding increase in Cell Index (proprietary readout, Roche application note). hTERT-HUVECs were treated with patient-, cell line- or mouse model-derived exosomes. Cells were then serum starved and plated into the upper chamber of the CIM Plate. Migration towards the chemoattractant FBS was continuously monitored every two minutes for a period of 24 hours. Figure 6E shows that hTERT-HUVECs treated with KSHV-associated exosomes exhibited increased migration compared with cells treated with exosomes from control human plasma (red). This assay independently demonstrates that exosomes from patient PEL fluid, the BCBL1 PEL cell line, and a xenograft mouse model of KS confer an enhanced migration phenotype to hTERT-HUVEC cells.

Since IL-6 plays a significant role in KSHV pathogenesis, we analyzed the levels of IL-6 present in the scratch assay supernatants by ELISA (Figure 6F). hTERT-HUVECs treated with patient-derived exosomes secreted high levels of IL-6. IL6 secretion in response to exosome treatment was decreased when the exosome fraction was incubated with annexin V (p≤0.003). These experiments suggest that efficient exosome transfer drives enhanced cell migration, possibly through the increased induction of cytokines such as IL-6. Note, though, that these experiments did not distinguish between miRNA and protein components of the exosomes. In sum, the exosomal signature associated with KSHV-related malignancies could not only be a reservoir of clinically important diagnostic biomarkers but may also be a novel mechanism of paracrine signaling that mediates KSHV-associated pathogenesis and tumorigenesis.

## Discussion

Circulating miRNAs, especially those within exosomes, have emerged as novel biomarkers [31], [32], [33], [94], [95]. Their main advantage is stability and ease of detection as all miRNAs can be profiled with a common platform. We previously established and validated such a miRNA profiling platform [54]. Bodily fluids such as plasma can be obtained using minimally invasive techniques and lend themselves to repeat sampling, for instance to follow therapy. In the case of PEL, periodic (in extreme cases every few days) draining of pleural cavities is medically indicated.

Although the exosomal miRNA profile of malignancies associated with EBV have been previously reported [44], [45], this is the first study to examine the circulating miRNA profile of KSHV-associated cancers. This is also one of a few studies to compare patient tumors to xenograft mouse models [96]. We extend previous findings on exosomal miRNAs, which were largely based on cell culture models. KSHV-encoded miRNAs were detectable in systemically circulating exosomes (Figure 2I and Figure 3), including in xenograft mouse models of KS. This suggests that viral miRNAs can have effects far from the site of the infected cell. Furthermore, viral microRNAs could potentially serve as highly specific biomarkers of KSHV-associated malignancies, particularly if the lesions are internal and comprised of mostly latently infected cells. We found similar levels of viral miRNAs in exosomes derived from latently infected PEL cells compared to PEL cells undergoing lytic reactivation (Figure 3A). Most KS tumor cells and most PEL are latently infected and even if lytic gene expression is observed in a subset of cells, virions are seldom produced [97], [98].

A significant complication of characterizing exosomal miRNAs in virally associated diseases is that miRNAs may be incorporated into virions. Previous studies have shown that viral RNAs can be detected within herpesvirus virions, including KSHV and EBV [99], [100]. Recently, Lin et al. demonstrated the presence of viral, as well as cellular miRNAs in purified KSHV virions [64]. Exosomes are difficult to physically separate from virions due to their similar sedimentation velocities, buoyant densities, biogenesis and heterogeneous nature of exosomes [44], [46]. Others have circumvented this issue using cell culture models that are incapable of virus production, such as HCV subgenomic replicon (SGR) cells [46]. Analogous to this model, we employed several latent models of KSHV infection, including the latently infected TIVE xenograft mice, the latency locus transgenic mice and the BCBL1 latent PEL cell line [48], [50]. We believe that the majority of miRNAs we detect here are exosomal, rather than virion-associated. To support this interpretation, we offer three lines of evidence.

First, we were able to detect all viral miRNAs in latent BCBL1 exosomes and filtering samples led to decreased viral load but did not significantly affect levels of KSHV miRNAs (Figure 3, Figure S7). We detected similar amounts of KSHV miRNAs in exosomes isolated from latent PEL supernatant as in exosomes from supernatant of induced PEL (Figure 3). In the same samples, we observed a greater than 10-fold increase in viral DNA. This suggests that KSHV miRNAs are released into exosomes from latently infected PEL, analogous to exosomal EBV miRNAs which are released from latently infected cells [44], [96]. Note, that we are able to detect KSHV miRNAs in exosomes from 250 µl of latently infected cell supernatant, whereas at least 500 mls were previously used to enrich for virion-associated miRNAs [64]. We could also detect KSHV miRNAs in the bloodstream of mice, which carry KSHV latently-infected TIVE-E1/L1 xenografts. These cells do not generate infectious virions [48] and (R. Renne, personal communication).

Second, we were able to isolate exosomes by CD63-mediated affinity purification (Figure 3). Herpesvirus virions and exosomes co-purify in almost all centrifugation schemas designed to enrich for exosomal fractions (i.e. differential ultracentrifugation, sucrose gradients, ExoQuick solution). By contrast, anti-CD63 Dynabeads positively select exosomes which carry CD63 as one of their surface markers [57], [101] while CD63(−) virions are eliminated. This resulted in an enrichment of KSHV miRNAs and concomitant depletion of viral DNA (Figure 3), demonstrating that indeed viral miRNAs are present in exosomes. We were also unable to detect any contaminating virions in our samples enriched for exosomes by electron microscopy and structural viral proteins were absent in our exosome-enriched samples (Figure 2A and Figure S8).

Thirdly, KSHV miRNAs could be detected in exosomes isolated from the serum of our xenograft mouse model. These xenograft mice harbor latently infected cells, which do not generate infectious virus. This suggests that viral miRNAs are constantly released and circulate systemically in exosomes in mice (and patients) who harbor KSHV latently infected cells. Taken together, these data suggest that KSHV latently infected cells can release viral miRNAs and further demonstrates that exosomes are the source of these circulating miRNAs.

Human oncogenic miRNAs were easily detected in tumor-derived exosomes isolated from patient plasma and pleural fluid (Figure 4). Further analysis confirmed increased levels of the well-studied miR-17-92 cluster miRNAs. Our data also show potentially important similarities and differences in the miRNA profile from AIDS patients with KS compared to patients with other non-viral AIDS-associated malignancies (Figure 4F). This subset of exosomal miRNAs could reflect differences between the varying progression of different malignancies in AIDS patients or similarities among AIDS-associated cancers and merits further study. Exosomal miRNAs are readily detected in pleural fluid samples, representing an alternate sample source with potentially higher correlation to disease state for patients with malignant effusions. Since pleural fluid is more proximal to the tumor site than plasma, which circulates throughout the body, we reason that the circulating miRNome from malignant effusions may be more reflective of the tumor itself. However, further studies comparing the miRNA signatures of pleural fluid-derived exosomes from PEL and other non-KSHV-associated malignancies such as lung cancer are necessary to reveal diagnostic biomarkers unique to PEL.

We also demonstrated that human and viral miRNAs are present in circulating exosomes in xenografted mice (Figures 2,4). We used the TIVE L1 [48] xenograft model, which has been shown to be predictive of anti-KS therapies [98], [102]. The KSHV miRNAs that we consistently detected in these mouse models could only stem from the human graft. Due to the high conservation of cellular miRNAs within the oncogenic clusters, the cross-species detection of miRNAs using the human assays makes it difficult to distinguish miRNAs of human versus mouse origin in these models (Table S3, ABI product information, miRBase). In some cases, the mature miRNAs share 100% sequence homology across the entire length, not just the seed region (miRBase, [103]) and in many cases the targets have co-evolved as well [69]. We observed greater levels of miRNAs in the mouse exosomes compared to human exosomes, which may be due to the fixed 250 µl sample size with respect to the overall amount of blood circulating within a human (approximately 5 L) or mouse (approximately 0.0015 L).

Specific host miRNA markers of tumorigenesis also emerged in our mouse models. We showed previously that host miRNAs are distinct for different stages of KS tumor progression [6]. Therefore, tumorigenic miRNAs combined with viral miRNAs would offer a very specific biomarker signature and may also identify biomarkers for other related cancers. Therefore, we analyzed expression of the oncogenic miR-17-92 and 106b/25 clusters and found that they were significantly enriched in exosomes from TIVE tumor-bearing mice compared with controls (Figure 5). Several of the oncogenic miRNAs expressed in exosomes were previously found at highly expressed levels in the TIVE cell line independently (R. Renne, personal communication). The mir-17-92 cluster was previously shown to be upregulated in KS tumor biopsies [3], [4]. This is the first demonstration that the miR-17-92 cluster miRNAs are incorporated into exosomes from KSHV-associated malignancies. These oncogenic miRNAs have also been detected in exosomes derived from leukemia cells and those derived from breast milk [56], [104], suggesting that their function is at least in part to mediate paracrine phenotypes. Viral and cellular miRNAs originating from the tumor enter the mouse circulatory system and are readily detected in serum. Since our mouse model exosome signatures recapitulate the clinical KS signatures, this supports the validity of xenograft mice as a reliable model system for KS.

We also observed a subset of miRNAs that were highly induced in exosomes and were virtually undetectable in the free, circulating miRNA fraction (Figure 5D). The miRNAs detected exclusively in the exosome fractions are either known to be oncogenic or shown to be upregulated by KSHV infection [105], [106]. This suggests that certain miRNAs are preferentially incorporated into exosomes and that many proliferative and tumor-associated miRNAs fall into this class. Recently, Palma et al [107] found that selectively exported miRNAs from malignantly transformed cells may be incorporated into customized exosomal particles distinct from the microvesicles that originate from untransformed cells. It is conceivable that these have different systemic stability and thus become enriched in a blood sample. This may be the case with KSHV-induced miRNAs as well since we found miRNAs originating from our transgene model also to be enriched in this fraction.

Exosomes serve as a means of intercellular communication with surrounding cells and the contents of exosomes can be shared between cells through the mechanism of exosomal transfer [44], [45]. Exosomes can deliver functional miRNAs to recipient cells and consequently downregulate expression of target genes [44], [45], [108]. Leukemia cell-derived exosomes have recently been shown to affect endothelial cell function through microRNA transfer [56]. Moreover, tumor-derived microRNAs were recently reported to play a functional role through binding to Toll-like receptors, thereby inducing an inflammatory response and influencing tumor growth and metastasis [109]. This *in vivo* relevance was further demonstrated by inhibiting tumor-secreted miRNAs, which altered tumor formation in mice [109]. Dendritic cell-derived exosomes can be used to prime the immune response as cancer immunotherapy to suppress tumor burden [110], [111], [112]. Exosomes have also been recently tested in clinical trials to reduce tumor size [110], [111], [112], [113]. Collectively, these studies further demonstrate the *in vivo* relevance of exosomes and their potential as mediators of disease phenotypes.

In this study, we find stable, systemic KSHV miRNAs and oncomiRs. GO pathway analysis of predicted targets of the oncogenic miRNAs expressed in exosomes revealed a variety of pathways targeted by KSHV during pathogenesis (Table 1). Since several of these pathways shared a role in cell migration, we further tested the effects of patient-derived exosomes on migration of hTERT-HUVECs. Treatment of cells with exosomes from pleural fluid led to earlier, enhanced migration of endothelial cells, giving these patient-derived exosomes a functional biological role (Figure 6). Therefore, it is possible that miRNAs specifically expressed within exosomes play a role in disease progression and mediate paracrine effects, which are a hallmark of KSHV tumorigenesis.

## Materials and Methods

### Sample preparation

De-identified human plasma samples were obtained from healthy controls, patients enrolled in the UNC AIDS Malignancy Trial IRB#09-1201 (diagnosed with KS or other, non-KS malignancies) and patients with Kaposi's sarcoma. Primary pleural effusion fluid from three patients was also obtained. For mouse controls, pooled plasma from C57/BL6 mice was obtained from Innovative Research (Novi, Michigan). Blood was collected from C57/BL6 control mice and serum was isolated using a serum-gel tube (Sarstedt). Blood sera were also purified from KSHV latency locus (801) transgenic mice [50] and Balb/c mice injected with TIVE, latently infected KSHV+ endothelial cells [48]. Purified serum was collected for each group. Samples from each group were pooled as shown in Table S1 to control for individual genetic variation among sample groups and to increase the material available for exosome isolation. Exosome isolations for each sample group were performed in duplicate.

### Ethics statement

The mice were held in UNC animal facilities. Veterinary care was provided by the University veterinarians and support animal care staff. The animal facility is an American Association of Accreditation of Laboratory Animal Care (AAALAC) accredited facility. The mice were maintained according to AAALAC guidelines and approved by institutional animal care and use committee (IACUC) under protocol #10-247/“KSHV latency mice”. The UNC Chapel Hill animal welfare assurance number is: A-3410-01.

### Isolation of exosomes and free, circulating miRNAs using Exoquick

Human plasma, pleural fluid, mouse plasma or serum was centrifuged at 300×g for 10 minutes to pellet any cells. 250 µl of supernatant was transferred to a fresh tube and incubated with 63 µl Exoquick precipitation solution as per the manufacturers' instructions (System Biosciences, Mountain View, California). After incubation for 16 hours at 4°C, contents of each tube were centrifuged for 30 minutes at 1,500×g to pellet exosomes. The supernatant containing free, circulating miRNAs was transferred to a fresh tube and the exosomal pellet was resuspended in 100 µl of nuclease-free, PCR-grade water (Life Technologies, Carlsbad, California). Other studies also have validated the Exoquick protocol and have not detected any significant differences in exosome populations compared with ultracentrifugation methods [56].

### Isolation of exosomes by ultracentrifugation

Patient pleural fluid and tissue culture supernatants (35 mls) were centrifuged for 30 minutes at 2,000×g to pellet cells. The supernatant was transferred to a fresh tube and centrifuged at 12,000×g for 30 minutes at 4°C. Filtering was performed after clearance of cellular debris and prior to ultracentrifugation where noted. Supernatants were transferred to ultracentrifuge tubes and spun in a SW32Ti swinging bucket rotor for 70 minutes at 110,000×g. The supernatant was discarded and the pellet was resuspended in 35 mls of sterile PBS and passed through a 0.2-micron filter. Exosomes were centrifuged at 110,000×g for an additional 70 minutes to wash. The supernatant was again discarded and the pellet was resuspended in 1 ml of sterile PBS. Samples were transferred to 1.5 ml ultracentrifuge tubes and concentrated by ultracentrifugation at 110,000×g for 70 minutes using a TLA-100.3 rotor. The resulting pellet was resuspended in a small volume and used for subsequent experiments.

### Exosome enrichment using CD63+ Dynabeads

Samples (35 ml starting material) were ultracentrifuged as previously described to obtain exosome-enriched samples (∼500 µl). These samples were further enriched for CD63+ exosomes using the CD63+ Dynabead exosome isolation kit according to manufacturer's instructions (Invitrogen, Life Technologies #10606D). Briefly, 500 µl of sample was incubated with 100 µl CD63+ Dynabeads overnight at 4°C. Exosomes were positively selected using a Dynabeads magnet and samples were washed to eliminate non-specific binding. Bead-bound exosomes were resuspended in 300 µl PCR-grade water and approximately 100 µl was used as input for further RNA, DNA and protein analysis by Western blot and qPCR (Figure 3). Prior to DNA isolation using the Magnapure automated system (Roche), beads were treated with Proteinase K (200 µg/ml) for 2 hours at 55°C to dissociate beads and exosomes.

### Filtering of samples for exosome enrichment

To obtain filtered samples, cell supernatants or patient fluids were first cleared of cellular debris. The resulting supernatant was passed through either a (1) Nalgene 250 ml Rapid-flow filter unit, 0.2 µm CN membrane, 50 mm diameter (Thermo Scientific, #126-0020) for ultracentrifugation and Dynabead methods or (2) Whatman Puradisc 25AS 0.2 µm polyethersulfone membrane filter (#6780-2502) for the ExoQuick methods. The flow-through was then used as input for downstream exosome enrichment protocols (ExoQuick, ultracentrifugation and CD63+ Dynabeads). Flow-through did not seem to affect exosome yield or loss of exosomal markers (Figures 2, Figure S6 and data not shown). Filtration of samples resulted in a decrease in KSHV load as determined by qPCR for LANA DNA (Figure 3).

### RNase treatment

Select samples were treated with RNase prior to exosome isolation. RNase treatment was performed as described previously [34]. Briefly, samples were incubated with RNase (Roche – product # 11119915001, includes both RNase A and T) at 37°C for 30 minutes to destroy any freely circulating RNAs (Figure S4). Exosomes were then isolated using the ExoQuick precipitation solution.

### Electron microscopy

Aliquots of purified exosome samples were absorbed directly onto glow-charged thin carbon foils on 400-mesh copper grids without fixation and stained with 2% (w/v) uranyl acetate in water. The grids were examined in an FEI Tecnai 12 (Hillsboro, OR) electron microscope at 80 kV. Images were captured on a Gatan Orius CCD Camera (Gatan, Pleasanton, CA) using Digital Micrograph software. Images for publication were arranged and contrast optimized using Adobe Photoshop CS4.

### RNA isolations and spike-in control

The supernatant and exosome fractions from each pooled group were used in full as input for RNA isolations. Total RNA was isolated using TRI reagent (Molecular Research Center, Cincinnati, Ohio) followed by a phenol/chloroform extraction and ethanol precipitation of RNA as previously described [3], [4], [6]. Prior to RNA isolation, 25 fmol of *C. elegans* cel-mir-39 RNA was added to each sample as a spike-in control [33], [35]. Total RNA was resuspended in nuclease-free, PCR-grade water and the RNA concentration was determined using the NanoDrop spectrophotometer (Thermo Scientific, Waltham, Massachusetts).

### Western blot analysis

Exosomes were isolated using the Exoquick kit as described above. Both exosomal fractions and supernatants were lysed in 100 µl NP40 lysis buffer (50 mM Tris, 150 mM NaCl, 1% NP-40 with 50 mM NaF, 1 mM sodium vanadate, 30 mM beta-glycerophosphate, 1 mM PMSF and protease inhibitor cocktail (Sigma, St. Louis, Missouri). Lysates (10 µl) were run on a 10% SDS-PAGE gel, transferred to a nitrocellulose membrane (Hybond, GE Healthcare, Pittsburgh, Pennsylvania) and blocked in 5% dry milk in Tris-buffered saline with 0.1% Tween 20 overnight at 4°C. CD9 was detected using the CD9 EXOAB antibody kit as per the manufacturer's instructions (System Biosciences, Mountain View, California). Anti-flotillin-2 (BD #610383) was used at 1∶5000 and anti-beta actin (Sigma #A2228), anti-Hsp90 alpha (Assay Designs #SPS-771) and anti-Hsp90 beta (Assay Designs #SPA-842) were used at 1∶2000. Secondary HRP antibodies (Vector Labs Cat# PI-1000 – rabbit, Cat#PI-2000 – mouse, Burlingame, California) were used at 1∶10,000 and blots were developed using Pierce ECL Western blotting substrate (Pierce, Rockford, Illinois).

### Taqman profiling of cellular and viral miRNAs

Approximately 1 µg of total RNA in 75 µl PCR-grade water was DNase-treated using the Turbo-DNase kit (Life Technologies, Carlsbad, California). The RNA was run on an Agilent RNA Nano 6000 chip to assess RNA quality and the presence of small RNA populations. Next, samples (200 ng) were used as input for cDNA synthesis using the Megaplex RT kit version 3.0, Human Pools A and B (Life Technologies, Carlsbad, California). Following cDNA synthesis, samples were further amplified using the Megaplex PreAmp kit version 3.0, Human Pools A and B (Life Technologies, Carlsbad, California). The PreAmp product was diluted 5-fold and the amplified cDNA samples were used as previously described [54] using a library of 754 Taqman cellular miRNA primers (Life Technologies, Carlsbad, California) and a robotic pipetting system for automated plate setup [54] (Tecan, Männedorf, Switzerland). qPCR reactions were run on a Lightcycler 480 (Roche, Indianapolis, Indiana). Automated plate setup and replicates correlated well, with little standard deviation between replicate CTs and no significant quadrant errors (Figure S17, average standard deviation among 4 replicates = 0.35 CT). Reactions were also performed to detect levels of the spike-in control *cel-mir-39* and the KSHV miRNAs using individual Taqman RT and qPCR miRNA assays (data not shown). PCR products of KSHV miR-K2 were run on an HTDNA 1K chip on the Caliper LabChip GX (Caliper Life Sciences, Hopkinton, Massachusetts) to confirm the results via gel electrophoresis.

### Analysis of qPCR-based miRNA profiling data

In-depth statistical analysis of technical replicates was performed in R and revealed little variation in CTs below 45. CT variation among the same sample in each of 4 quadrants was also assessed and no significant deviation was observed (Figure S17). Cycle threshold (CT) values for each sample were averaged across two technical replicates (one replicate from each exosome isolation) and those with a CT greater than 45 were excluded and recorded as negative. The remaining data were assigned expression scores based on a specific range of CT values. The CT range of expression was 20–45, with CT = 20 as the highest expression score (expression score = 3) and CT = 45+ yielding the lowest score of 0. Expression scores were assigned in increments of 0.5, with one expression class including a range of 4 CTs. Therefore, any significant difference reported was confirmed as a difference greater than 4 CTs or approximately 16-fold. The expression scores were then subjected to unsupervised classical clustering with Pearson coefficient using Array Miner™ (Optimal Design, Brussels, Belgium). PCA three-dimensional clustering figures and heatmaps of miRNA expression are shown.

### KSHV viral load assay

Exosomes were isolated using the Exoquick kit as described above (System Biosciences, Mountain View, California). Exosome pellets and supernatants containing free, circulating miRNAs and proteins were resuspended in 200 µl PCR-grade water. Exosome-enriched samples were then treated with DNase for 30 minutes at 37°C according to manufacturer's instructions for the Turbo DNA-free kit (Ambion, Life Technologies). DNase-treated samples were then adjusted to 500 µl volume and used as input for DNA extraction on the Magnapure (Roche, Indianapolis, Indiana) using the large volume kit and program settings for total nucleic acid from plasma samples. DNA was eluted in 100 µl total volume. Extracted DNA (5 µl) from each sample was used to determine the presence of KSHV using primers: F primer 5′-GGAAGAGCCCATAATCTTGC-3′; R primer 5′- GCCTCATACGAACTCGAGGT-3′. Ten-fold dilutions of a KSHV oligonucleotide target with the following sequence were used to generate a standard curve: 5′-GGAAGAGCCCATAATCTTGCACGACTCAGACCTGGAGTTCGTATGAGGC-3′. PCR products were then loaded on an HTDNA 1K chip on the Caliper LabChip GX (Caliper Life Sciences, Hopkinton, Massachusetts) to confirm the presence of KSHV DNA.

### GO pathway analysis

Oncomirs that were upregulated in the KSHV-associated sample groups were input into the microRNA target prediction database (MetA MicroRNA target Interference (MAMI), http://mami.med.harvard.edu/). The settings used for target prediction were highest stringency and included only 3′UTR target sites. The Entrez IDs of the predicted targets were used as input for the GO pathway database DAVID. The KEGG pathway terms of highest correlation were determined along with statistical significance (P value) and the number of predicted targets in each pathway. Specific pathways involved in migration were determined by searching peer-reviewed literature that included mechanistic data for migration and each specific pathway. Pathway analysis was performed for microRNAs induced in KSHV-associated malignancies from our exosome study and for microRNAs induced by WNV infection in hTERT-HUVEC cells.

### Scratch assay

hTERT-HUVEC cells [92] were seeded at 80% confluence in a 24-well plate and allowed to equilibrate overnight before treating with exosomes for a period of 24 hours. Annexin blocking was performed as previously described [44]. Briefly, exosomes or supernatant were incubated with Annexin V-FITC for 1 hour at room temperature prior to adding to cells. Cells were grown in EGM-2 media with all supplements (Lonza, EGM-2 Bulletkit). Each well was scratched using a standard 200 µl pipette tip and the location of the scratch was marked to locate the initial scratch at subsequent time points. Cells were washed with media to eliminate floating cells and replaced with fresh media immediately after the wound initiation. Images were captured at 0 h, 8 h and 16 h after the initial scratch. Images are shown at 100× magnification and were obtained on a Leica DMIL microscope using a HI Plan 10×/0.25 PHI objective and QImaging camera (Cooled color, RTV 10 bit) paired with QCapture imaging software 3.0.

### xCelligence migration assay

hTERT-HUVEC cells were treated with exosomes isolated using the ExoQuick method. After 24 hours of incubating with exosomes, cells were serum-starved for 6 hours and then lightly trypsinized for 3 minutes to detach cells. Trypsin was inactivated with media containing FBS and cells were centrifuged at 300 g for 5 minutes. Cells were washed with PBS and the remaining pellet was resuspended to a concentration of 300,000 cells/ml in serum-free EBM-2 media (Lonza). 30,000 cells were plated per well of the upper chamber of an xCelligence CIM Plate 16 (Acea Biosciences). Prior to CIM plate assembly, both sides of the membrane were coated with 20 µg/ml fibronectin. Media containing FBS was placed in the lower chamber as the chemoattractant. The upper and lower chambers of the CIM plate were assembled and reads were taken every 2 minutes for a period of 24 hours using the RTCA DP xCelligence instrument (Acea Biosciences).

### IL-6 ELISA

Supernatants from the scratch assay (hTERT-HUVECs treated with exosomes) were analyzed for levels of IL-6 using ELISA according to manufacturer's protocol (eBioscience, #88-7066-88). Briefly, supernatants were collected at 16 hours post-scratch and were diluted 1∶10 for ELISA. A standard curve of IL-6 positive control was generated and levels of IL-6 (pg/ml) were calculated. The average of three technical replicates of two independent experiments was calculated.

## Supporting Information

Figure S1 **A small number of miRNAs account for the majority of the miRNA reads in Herpesvirus-associated lymphoma.** (A) microRNA sequencing data of control tonsil, EBV-negative PEL and EBV-positive PEL. Cellular microRNAs are shown in blue, EBV microRNAs in red and KSHV microRNAs in green. Small RNAs were isolated and subjected to sequencing using Illumina methods and reagents. Raw read counts for each miRNA were transformed by taking the log of the square root of the counts. The majority of the microRNA reads could be attributed to expression of only a few microRNAs. Comparison of the EBVnegPEL and EBVposPEL to tonsil shows that a few viral microRNAs dominated the overall microRNA profile. (B) Quantile-Quantile (QQ) probability plot of the miRNA sequencing count data. Transformed counts (log of the square root of counts) were plotted against the standard deviation from the mean. The dotted line represents the expected data for a normal distribution. However, the solid gray line demonstrates that the sequencing count data does not follow normal distribution and instead several microRNAs (top right) are highly expressed and others are expressed at levels lower than expected with a normal distribution (bottom left).
(TIF)

Figure S2 **Immunohistochemistry of KS and xenograft mouse models.** Representative hematoxylin and eosin staining of the xenograft mouse model and KS tissue. The panels highlight the differences between primary KS and a xenograft mouse model of PEL, xPEL. The xL1 TIVE model is the most representative of KS, which is also reflective of our profiling results. Note the different morphological features of xPEL and xL1.
(TIF)

Figure S3 **Exosomal RNA consists of small RNAs but lacks rRNA.** Total RNA samples isolated from BCBL1 PEL cells or BCBL1-derived exosomes were run on the Agilent to assess RNA content. Samples were loaded onto an Agilent RNA Nano 6000 chip and shown are the resulting electropheregrams. In the cellular RNA sample, ribosomal RNA was detected, noted by the 18S and 28S subunit peaks. Cellular RNA also contained small RNAs, denoted by the small peak on the left. The lower marker also yielded a peak. The exosome samples only contained small RNAs and lacked both ribosomal RNA subunits as expected.
(TIF)

Figure S4 **RNase treatment efficiently eliminates freely circulating microRNA.** 25 fmol of *C. elegans cel-mir-39* was added to each pleural fluid sample. For use as a spike-in internal control, cel-mir-39 was added after a 5 minute incubation with Triazol, prior to addition of chloroform. This is denoted as “spike-in”. The intrinsic RNase activity of pleural fluid was also assessed by incubating cel-mir-39 with pleural fluid at 37°C for 30 minutes. Pleural fluid did exhibit some intrinsic RNase activity. To verify the activity of RNase treatment, cel-mir-39 was added to pleural fluid and a mix of RNase A/T (Roche) was added at either 10 or 100 U/ml. Samples were incubated at 37°C for 30 minutes followed by RNA isolation. As a negative control, levels of cel-mir-39 were assessed in pleural fluid samples without the cel-mir-39 spike-in. Samples were normalized using equal input and values are shown as dCT of the sample without cel-mir-39. ND, not detected or below the limit of detection.
(TIF)

Figure S5 **ExoQuick purification of exosomes yields EM images with high background that is not due to cellular debris.** Samples of primary pleural fluid were enriched for exosomes using the ExoQuick protocol according to manufacturer's recommendations. Negative staining of exosomes was performed and EM images were taken under the conditions described. Scalebars are shown for each image. (A) Exosomes enriched using the standard ExoQuick protocol. (B) Pleural fluid samples were spun at 10,000×g for 30 minutes to eliminate cellular debris prior to performing the ExoQuick protocol. (C) Exosomes were enriched using the ExoQuick protocol and then overlayed onto a 30% sucrose cushion. The sample was then centrifuged at 10,000×g for 1 hour and the bottom fraction containing exosomes was imaged. Note, the particulate matter has a diameter <30 nm, i.e. smaller than exosomes or virions.
(TIF)

Figure S6 **Exosome populations express exosomal markers regardless of isolation technique.** Exosomes isolated by ultracentrifugation (UC) or Exoquick (EQ) protocols were analyzed for purity. (A) The exosomal markers Hsp90 alpha (A, B), Hsp90 beta (C), flotillin-2 (D) and beta actin (E) were analyzed by Western blot in human samples isolated by UC and EQ techniques. All exosome markers were detected in pleural fluid exosomes isolated by either ultracentrifugation (UC) or Exoquick (EQ) solution, confirming that both of these protocols yield populations of purified exosomes. Since these markers are cellular proteins that are enriched in exosomes (ExoCarta, www.exocarta.org), these proteins were also expressed in cell lysates from BCBL1 PEL cells (denoted as “Cells”). As expected, the exosomal markers were absent in the supernatant fraction. Note, only 250 µl of pleural fluid was used as input for the Exoquick protocol and ∼35 mls pleural fluid was used as starting volume for the ultracentrifugation method. Sample were normalized by equal input volumes for each technique.
(TIF)

Figure S7 **KSHV miRNAs are not significantly affected by filtering of exosome-enriched samples.** Supernatants from latently infected BCBL1 cells were collected and exosomes were isolated using the ExoQuick method. Prior to ExoQuick enrichment, supernatants were passed through a 0.2 micron Whatman filter to decrease the amount of virus present in the sample. RNA from BCBL1 cells was used as a positive control for expression of KSHV miRNAs. PCR-grade water was used as input for the cDNA reaction for a no template control (NTC). Total RNA was isolated using Triazol and samples were normalized to equal amounts of total RNA input. Taqman microRNA assays (Life Technologies) for 14 KSHV miRNAs were used to determine levels of expression by qPCR, which are shown as fold above background for three technical replicates. qPCR products were diluted 1∶10 and run on a Caliper nanofluidics platform (similar to traditional gel electrophoresis). The KSHV miRNA products run at ∼60 bp with the primers and Taqman probe annealed. Cells, exosomes and filtered exosomes expressed KSHV miRNAs to similar levels. A shifted band consisting of the primer dimers can be seen in the NTC lanes. Abbreviations: Exo, exosomes; Exo-F, filtered exosomes; NTC, no template control.
(TIF)

Figure S8 **The structural protein K8.1 is not detected within exosome fractions.** The BCBL1 PEL cell line was cultured and cells were reactivated with either 1.25 mM sodium butyrate (NaB) and 20 ng/ml TPA or 1.25 mM vorinostat. After 48 hours, latent and reactivated BCBL1 cells were collected. Pleural fluid-derived exosomes (PF exo) were isolated using ExoQuick and all samples were lysed in NP40 buffer containing proteinase (Sigma) and phosphatase inhibitors. Samples were loaded onto a 10% polyacrylamide-SDS gel and then transferred to a nitrocellulose membrane (Hybond). The membrane was blocked with 5% dry milk in TBST overnight and then incubated with primary K8.1 anti-mouse at 1∶100 followed by anti-mouse secondary antibody at 1∶5000. Blots were developed using Pierce ECL substrate kit and Blue Devil autoradiography film and a representative blot of three independent experiments is shown. K8.1 was only expressed in reactivated BCBL1 cells and was absent in both latently infected BCBL1 cells and PF-derived exosomes.
(TIF)

Figure S9 **Latency locus transgenic mice express a distinct miRNA signature compared to control mice.** Comparative analysis of exosomal miRNA profiles from transgenic mice encoding the KSHV latency locus (Tg400, Tg401) and control mice (Ctrl 61, Ctrl 64). Mouse serum (250 µl) from individual control and transgenic mice were used as input for the ExoQuick exosome isolation method. Following RNA isolation, cDNA was amplified and Taqman miRNA profiles of each sample were performed as described in the methods section. (A) Density distribution (red) and histogram (gray) of median CT of n: 384 miRNAs across all samples. (B) Cumulative density plot for the same data. (C) Waterfall plot of the median CT for each of the miRNAs in the transgenic mice is shown in red. White lines indicate median CT for control mouse samples. Yellow lines indicate 1, 2 and 3 standard deviations of the median CT of the positive sample, representing the expression percentile under the assumption of a normal distribution of the data. (D) Heatmap of CT values for all miRNAs in all samples (blue indicating absence, white intermediate and brown, highest levels of a given miRNA). Clustering was based on Ward's criteria and Manhattan distance metric.
(TIF)

Figure S10 **Profiling of oncomirs in transgenic and xenograft mouse models.** Heatmaps reflective of unsupervised clustering analysis are shown for oncomirs in mouse samples. Oncomirs from mouse models are shown as a series of panels (i–iv). Panel i compares oncogenic miRNA expression between control mice and individual TIVE xenograft mice. Profiling data from the transgenic mouse model encoding the KSHV latency locus is shown in Panel ii with control mice and the average of the TIVE xenograft data. Panels iii and iv show two separate clusters of miRNA expression and compare control and transgenic mice to the primary human PEL pleural fluid cases. Abbreviations of mouse samples are as follows: CMP – control mouse plasma, CMS – control mouse serum, TIVE 1–3, individual TIVE xenograft mice, tg – 801 latency locus mouse model, PF – primary human PEL pleural fluid, exo – exosomes, sup – exosome-depleted supernatants. The names of each microRNA in the cluster are shown to the right of each heatmap. Red denotes high expression, green denotes low expression and black is basal or intermediate expression.
(TIF)

Figure S11 **Profiling of oncomirs in human patient samples.** Heatmaps reflective of unsupervised clustering analysis are shown for oncomirs in human samples. Oncomir expression in human samples is shown as two separate clusters of expression. For the human oncomiR heatmap, sample lanes from left to right are: CHP sup pre-exo - control human plasma supernatant pre-ExoQuick, CHP E – control human plasma exosomes, CHP S – control human plasma exosome-depleted supernatant, RNase CHP E – RNase-treated CHP exo, RNase CHP S – RNase-treated CHP sup, AMT E – AIDS Malignancy, non-KS exo, AMT S – AIDS Malignancy, non-KS sup, KS E – Kaposi's sarcoma exosomes, KS S – Kaposi's sarcoma exosome-depleted supernatant, PF E – pleural fluid exosomes, PF S – pleural fluid exosome-depleted supernatant. The names of each microRNA in the cluster are shown to the right of each heatmap. Red denotes high expression, green denotes low expression and black is basal or intermediate expression.
(TIF)

Figure S12 **PF- and CHP-derived exosomal miRNA profiles respond similarly to RNase treatment.** Exosomes were isolated using the ExoQuick solution and equal volumes (250 µl) of pleural fluid (PF) or control human plasma (CHP). Samples were either left alone (−) or treated with 25 U/ml RNase (Roche) for 30 minutes at 37°C (+). RNA was isolated from exosome-enriched samples and the miRNA expression profile was determined by qPCR using Taqman microRNA assays from human pool A v2.1 (Life Technologies). Heatmap of CT values for all miRNAs in all samples (blue indicating absence, white intermediate and brown, highest levels of a given miRNA). Clustering was based on Ward's criteria and Manhattan distance metric. The heatmap shows that the majority of miRNAs increased in PF-derived exosomes were not affected by RNase treatment. Similarly, the expression profile of control human plasma exosomes did not significantly change in response to RNase treatment. This demonstrates that different patient samples have similar responses to RNase treatment and that exosomal miRNAs are largely RNase-resistant.
(TIF)

Figure S13 **Profiling of individual PEL patients reveals insight into variability of miRNA profiles.** Comparative analysis of exosomal miRNA profiles from individual PEL patient fluids (pleural fluid patients 1–3) and KS-free control fluids (CHP, control human plasma). Pleural fluid from individual PEL patients and control human plasma were used as input for the ExoQuick exosome isolation method. Following RNA isolation, cDNA was amplified and Taqman miRNA profiles of each sample were performed as described in the methods section. (A) Density distribution (red) and histogram (gray) of median CT of n: 384 miRNAs across all samples. Indicated in orange is the CT = 50 cutoff. (B) Cumulative density plot for the same data. (C) Waterfall plot of the median CT for each of the miRNAs in the PEL patient fluids (PF1-3) shown in red. White lines indicate median CT for KSHV negative samples (CHP a and b). Yellow lines indicate 1, 2 and 3 standard deviations of the median CT of the positive sample, representing the expression percentile under the assumption of a normal distribution of the data. (D) Heatmap of CT values for all miRNAs in all samples (blue indicating absence, white intermediate and brown, highest levels of a given miRNA). Clustering was based on Ward's criteria and Manhattan distance metric. MiRNAs were highly expressed in PF-derived exosomes compared to control exosomes. A large number of these PEL signature miRNAs were expressed in all three patients and could thus be novel biomarkers of PEL. Despite the inherent genetic variability among patients, we could identify multiple miRNAs that were expressed at high levels in all three PEL patients compared to controls.
(TIF)

Figure S14 **MiRNA profiling of KS biopsies and PBMCs from control human plasma.** Comparative analysis of miRNA profiles from KS biopsies and PBMCs from control human plasma (CHP pellet). Four primary KS biopsies were pooled to obtain the KS biopsy pool sample. (A) Density distribution (red) and histogram (gray) of median CT of n: 384 miRNAs across all samples. Indicated in orange is the CT = 45 cutoff. (B) Waterfall plot of the median CT for each of the miRNAs in the KS biopsy samples shown in red. White lines indicate median CT for CHP pellet sample. Yellow lines indicate 1 and 2 standard deviations of the median CT of the positive sample, representing the expression percentile under the assumption of a normal distribution of the data. (D) Heatmap of CT values for all miRNAs in all samples (blue indicating absence, white intermediate and brown, highest levels of a given miRNA). Clustering was based on Ward's criteria and Manhattan distance metric. KS biopsies display a unique miRNA profile compared to CHP cells, with increased levels of several oncogenic miRNAs in all 4 KS biopsies (also seen in Figure 5).
(TIF)

Figure S15 **Caliper gel analysis of oncomirs induced in tumor mouse models.** Gel electrophoresis analysis of qPCR products of known oncomirs that were upregulated in tumor mouse models in our profiling study. qPCR products were diluted 1∶5 with molecular grade water and run on the Caliper GX Labchip using the HTDNA 1K chip (Caliper Life Sciences, Hopkinton, Massachusetts). This chip functions analogous to a traditional agarose gel and shown is an image of the band migration. Induction of these microRNAs in tumor mouse models was confirmed by increased band intensity of the PCR product compared with control mouse serum (CMS). This confirms the specificity of the assay and that key oncogenic miRNAs are enriched in exosomes from our mouse models of KS. Abbreviations: CMS, control mouse serum, Tg, 801 latency locus transgenic mouse model, Xeno, SLK xenograft mouse model, Neg., no template control (PCR-grade water), MW (molecular weight ladder). The band intensity was measured using Image J gel analysis (rsbweb.nih.gov/ij/) and is shown in arbitrary units of band intensity.
(TIF)

Figure S16 **Circulating microRNA profiles of TIVE xenograft model and KS case studies.** Comparative analysis of exosomal miRNA profiles from human (PF, DG1, DG2), murine xenograft models (TIVE1, TIVE2, TIVE3) and KS-free control fluids (CHS, human serum and CMS, mouse serum) and non-template control (NTC). (A) Density distribution (red) and histogram (gray) of median CT of n: 384 miRNAs across all samples. Indicated in orange is the CT = 45 cutoff. (B) Cumulative density plot for the same data. (C) Waterfall plot of the median CT for each of the miRNAs in the KSHV positive samples (TIVE1, TIVE2, TIVE3, PF, DG1, DG2) shown in red. White lines indicate median CT for KSHV negative samples (CMS, CHS, NTC). Yellow lines indicate 1, 2 and 3 standard deviations of the median CT of the positive sample, representing 68.3, 94.5 and 97.7 percentile under the assumption of a normal distribution of the data. (D) Heatmap of CT values for all miRNAs in all samples (blue indicating absence, white intermediate and brown, highest levels of a given miRNA). Clustering was based on Ward's criteria and Manhattan distance metric. (E–G) Plots comparing the first three dimensions of the principal component analysis (PCA) using n = 184 highly changed miRNAs. These were selected based on a median absolute deviation (m.a.d.) >4 across all samples. These demonstrate clustering of the control samples and KSHV positive samples. (H) Histogram of the Eigenvalues of the dimensions of the PCA analysis. Higher Eigenvalues indicate a greater contribution of a given distribution to the data variability. (I) Expected false positive rate on the vertical axis compared to the number of significant tests on the horizontal axis. This was obtained by calculating p values of an unpaired, two-sided t-test, allowing for unequal variance for comparison of the positive samples (TIVE1, TIVE2, TIVE3, PF) to negative samples (CHS, CMS, NTC) for each primer followed by adjustment for multiple comparisons using q-value method. Less than a single false positive miRNA is expected within the top 20 differentially expressed miRNAs, which therefore constitute a class signature. (J) Density distribution (red) and histogram (gray) of unadjusted log10 (p values) from t-test. The orange line indicates p≤0.01. (K) Cumulative density distribution (red) and histogram (gray) of q values from t-test. (L) Comparison of log10(q value) on the vertical axis to median CT for a given miRNA in the KS positive group.
(TIF)

Figure S17 **Technical replicates of microRNA data reveal little variation using an automated liquid-handling system.** Technical replicates of microRNA expression data were obtained using a Tecan Freedom Evo liquid-handling system. (A) Comparison of the median CTs shows linear correlation between technical replicates. (B) For each 384-well plate, 96 primers are run against 4 samples in each of 4 quadrants. In this test set, the same sample was run in each of 4 quadrants. Analysis of the microRNA data from each quadrant was conducted and revealed the absence of any quadrant bias. Furthermore, it shows tight correlation between technical replicates for CTs up to ∼45. (C) The distribution of the median CTs are plotted against the standard deviation of the CT. This recapitulates that there is little variation in technical replicates at lower CTs (up to ∼40). However, for CTs between 45 and 55, there can be highly variable deviation from the median. (D) Q-Q plot showing that the microRNA data of technical replicates follows a normal distribution, which is represented by the dotted line.
(TIF)

Table S1 **Clinical human and mouse model samples used in exosome study.** Sample groups used for miRNA profiling are shown along with the number of samples pooled for each group (N).
(DOCX)

Table S2 **List of microRNAs included in the oncomiR and tumor suppressor array.** The Taqman microRNA primers used in the oncomiR and tumor suppressor array are listed. These ∼150 microRNAs include known oncomiRs and tumor suppressor microRNAs according to published literature and references are shown for each microRNA. A subset of these microRNAs were cross-referenced to the Quantimir cancer array (System Biosciences) for confirmation of oncomir status. MicroRNAs previously found to be altered in KSHV-associated malignancies are also noted with an “x”.
(DOCX)

Table S3 **Species cross-reactivity of Taqman microRNA assays.** Product information from Life Technologies, Applied Biosystems. Recommended controls and 158 of the Taqman microRNA assays are shown with their mature microRNA sequence, Taqman assay name and species cross-reactivity for human, mouse and rat. Many of the assays exhibit substantial species cross-reactivity due to the conservation of microRNA sequences.
(PDF)

Table S4 **Ingenuity Pathway Analysis of predicted oncomir targets.** Oncomirs used in the microRNA profiling array and induced in KSHV-associated exosomes were used as input for Ingenuity Pathway Analysis (www.ingenuity.com/products/pathways_analysis.html). Predicted targets were obtained using Ingenuity and were narrowed down to include only experimentally validated targets. These targets were then overlayed with the functional pathway tool and the top represented pathways are shown. The number of target genes involved in each pathway is shown along with the univariate p value as determined by Ingenuity software. Several pathways are bolded to denote their importance in tumorigenesis and KSHV pathogenesis. As a control, we used miRNAs induced by WNV infection of hTERT-HUVEC cells.
(DOCX)

Table S5 **Pathway analysis of predicted targets using the Panther Database.** Predicted targets were obtained through the Ingenuity program and only included experimentally validated microRNA targets. The Entrez IDS of predicted targets were used as input for the Panther database (www.pantherdb.org) [114]. Pathway analysis was performed and the number of target genes involved in the top pathways are denoted. WNV-induced predicted targets were used as a control experiment and revealed different target pathways. The total number of predicted target genes for the KS-associated exosomal microRNAs and WNV-induced microRNAs were 188 and 106, respectively. Differences between KSHV oncomir targets and WNV-induced targets were significant as determined by paired T test (p = 1.56E-06).
(DOCX)
