# Supplementary figures and images for: Systemically Circulating Viral and Tumor-Derived MicroRNAs in KSHV-Associated Malignancies
Source: PLoS Pathog. 2013 Jul 18;9(7):e1003484. doi: 10.1371/journal.ppat.1003484 (PMC3715412; doi:10.1371/journal.ppat.1003484)

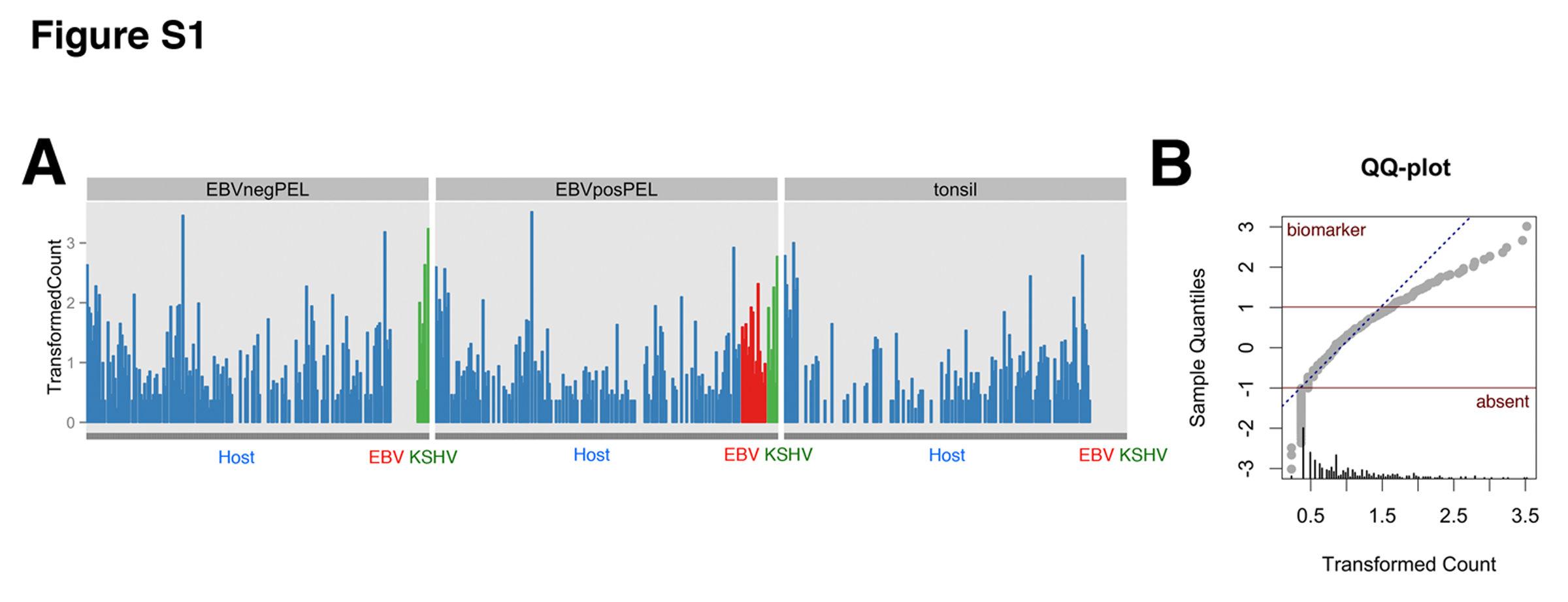

Supplement: Figure S1 — A small number of miRNAs account for the majority of the miRNA reads in Herpesvirus-associated lymphoma. (A) microRNA sequencing data of control tonsil, EBV-negative PEL and EBV-positive PEL. Cellular microRNAs are shown in blue, EBV microRNAs in red and KSHV microRNAs in green. Small RNAs were isolated and subjected to sequencing using Illumina methods and reagents. Raw read counts for each miRNA were transformed by taking the log of the square root of the counts. The majority of the microRNA reads could be attributed to expression of only a few microRNAs. Comparison of the EBVnegPEL and EBVposPEL to tonsil shows that a few viral microRNAs dominated the overall microRNA profile. (B) Quantile-Quantile (QQ) probability plot of the miRNA sequencing count data. Transformed counts (log of the square root of counts) were plotted against the standard deviation from the mean. The dotted line represents the expected data for a normal distribution. However, the solid gray line demonstrates that the sequencing count data does not follow normal distribution and instead several microRNAs (top right) are highly expressed and others are expressed at levels lower than expected with a normal distribution (bottom left). (TIF) [file ppat.1003484.s001.tif]

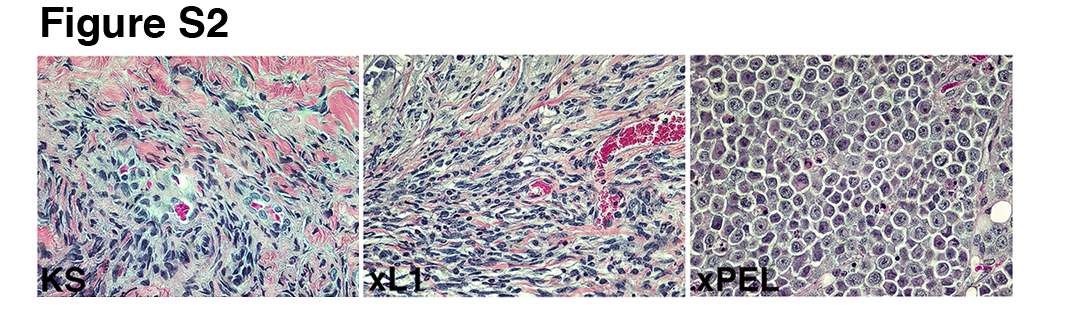

Supplement: Figure S2 — Immunohistochemistry of KS and xenograft mouse models. Representative hematoxylin and eosin staining of the xenograft mouse model and KS tissue. The panels highlight the differences between primary KS and a xenograft mouse model of PEL, xPEL. The xL1 TIVE model is the most representative of KS, which is also reflective of our profiling results. Note the different morphological features of xPEL and xL1. (TIF) [file ppat.1003484.s002.tif]

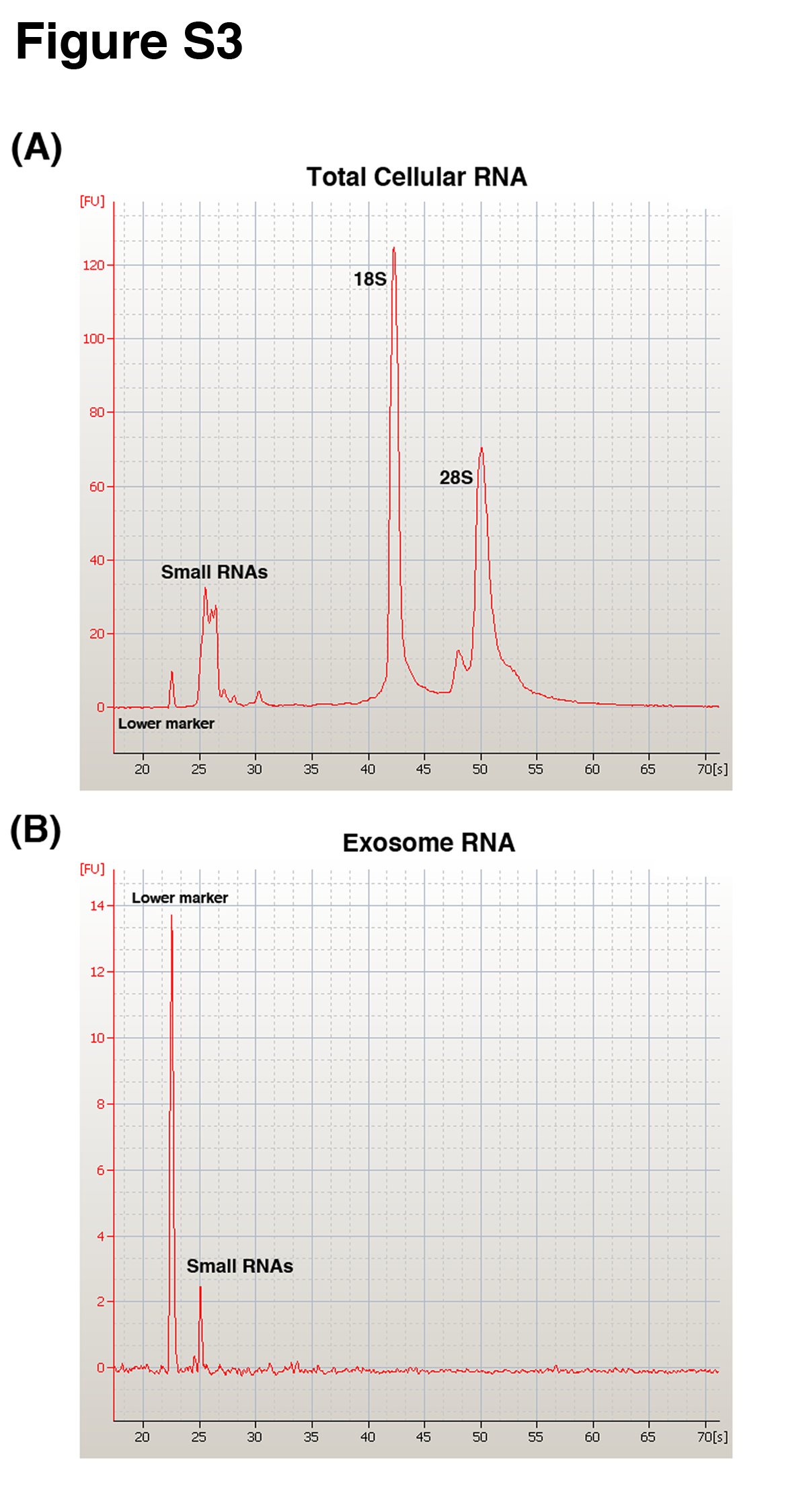

Supplement: Figure S3 — Exosomal RNA consists of small RNAs but lacks rRNA. Total RNA samples isolated from BCBL1 PEL cells or BCBL1-derived exosomes were run on the Agilent to assess RNA content. Samples were loaded onto an Agilent RNA Nano 6000 chip and shown are the resulting electropheregrams. In the cellular RNA sample, ribosomal RNA was detected, noted by the 18S and 28S subunit peaks. Cellular RNA also contained small RNAs, denoted by the small peak on the left. The lower marker also yielded a peak. The exosome samples only contained small RNAs and lacked both ribosomal RNA subunits as expected. (TIF) [file ppat.1003484.s003.tif]

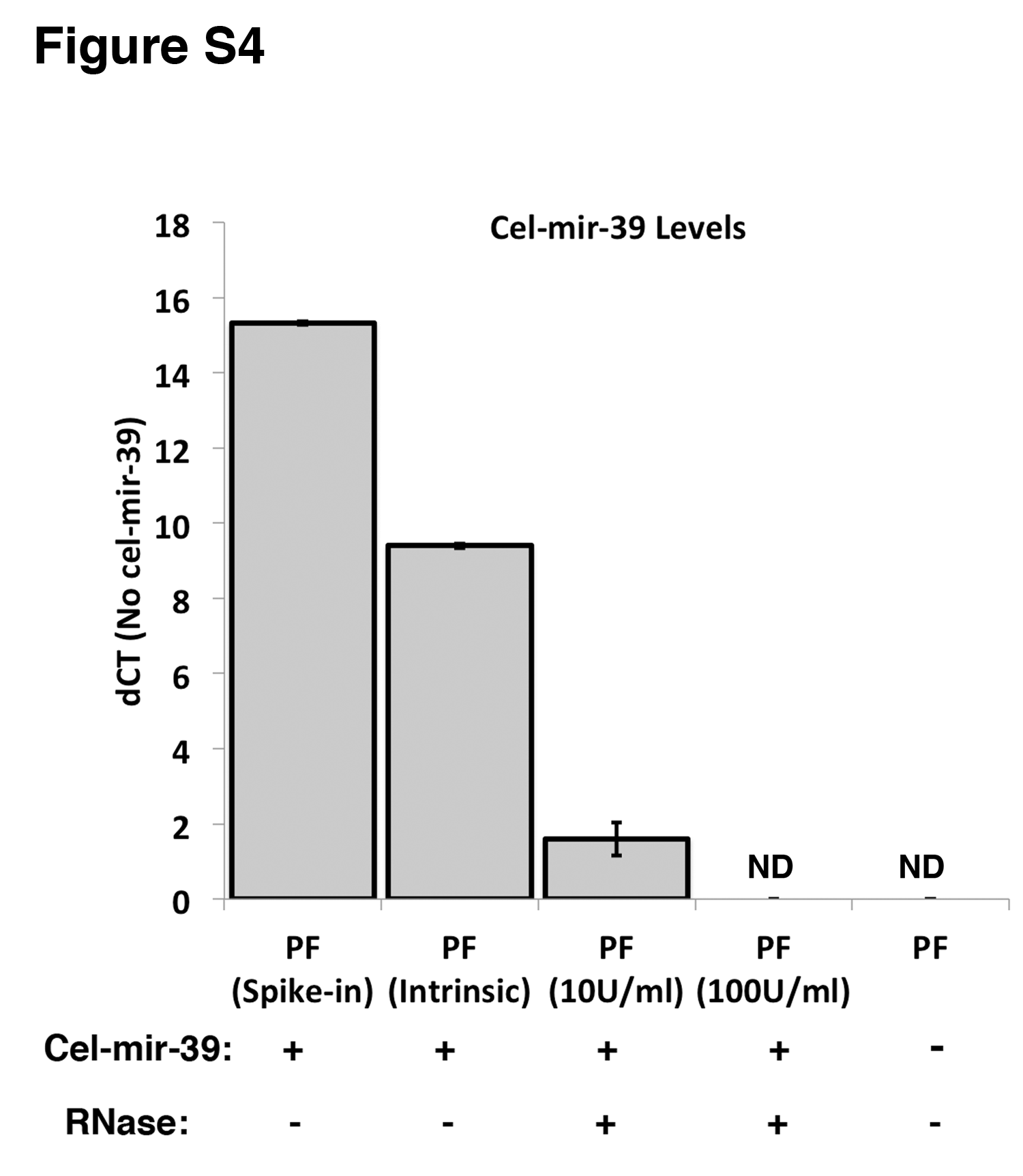

Supplement: Figure S4 — RNase treatment efficiently eliminates freely circulating microRNA. 25 fmol of C. elegans cel-mir-39 was added to each pleural fluid sample. For use as a spike-in internal control, cel-mir-39 was added after a 5 minute incubation with Triazol, prior to addition of chloroform. This is denoted as “spike-in”. The intrinsic RNase activity of pleural fluid was also assessed by incubating cel-mir-39 with pleural fluid at 37°C for 30 minutes. Pleural fluid did exhibit some intrinsic RNase activity. To verify the activity of RNase treatment, cel-mir-39 was added to pleural fluid and a mix of RNase A/T (Roche) was added at either 10 or 100 U/ml. Samples were incubated at 37°C for 30 minutes followed by RNA isolation. As a negative control, levels of cel-mir-39 were assessed in pleural fluid samples without the cel-mir-39 spike-in. Samples were normalized using equal input and values are shown as dCT of the sample without cel-mir-39. ND, not detected or below the limit of detection. (TIF) [file ppat.1003484.s004.tif]

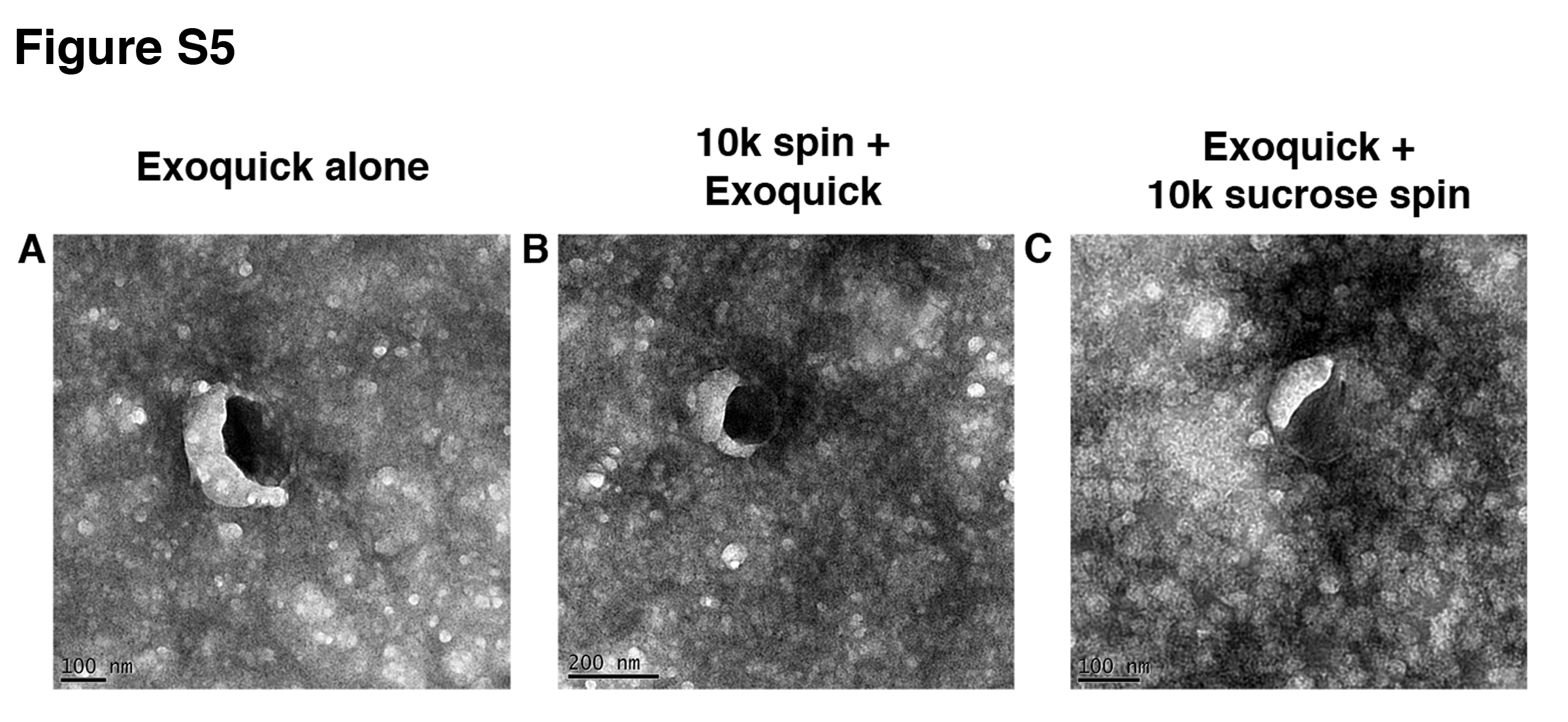

Supplement: Figure S5 — ExoQuick purification of exosomes yields EM images with high background that is not due to cellular debris. Samples of primary pleural fluid were enriched for exosomes using the ExoQuick protocol according to manufacturer's recommendations. Negative staining of exosomes was performed and EM images were taken under the conditions described. Scalebars are shown for each image. (A) Exosomes enriched using the standard ExoQuick protocol. (B) Pleural fluid samples were spun at 10,000×g for 30 minutes to eliminate cellular debris prior to performing the ExoQuick protocol. (C) Exosomes were enriched using the ExoQuick protocol and then overlayed onto a 30% sucrose cushion. The sample was then centrifuged at 10,000×g for 1 hour and the bottom fraction containing exosomes was imaged. Note, the particulate matter has a diameter <30 nm, i.e. smaller than exosomes or virions. (TIF) [file ppat.1003484.s005.tif]

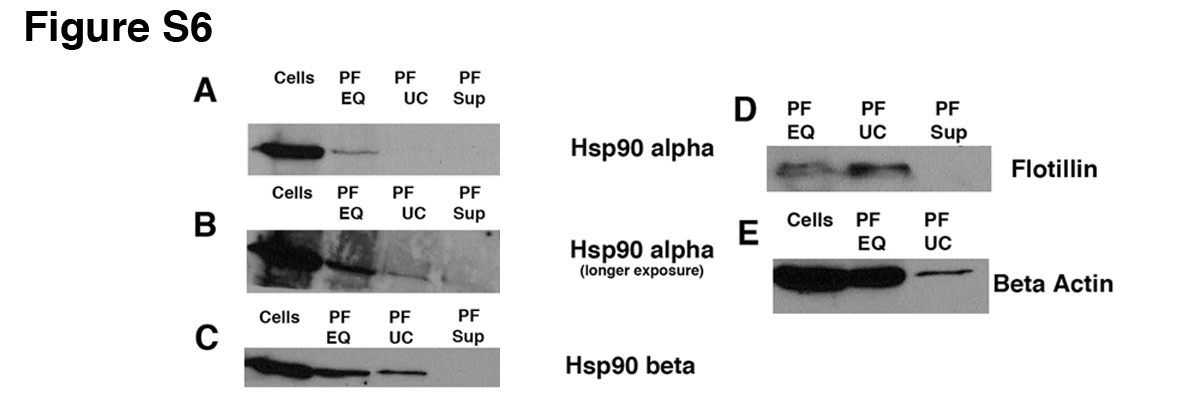

Supplement: Figure S6 — Exosome populations express exosomal markers regardless of isolation technique. Exosomes isolated by ultracentrifugation (UC) or Exoquick (EQ) protocols were analyzed for purity. (A) The exosomal markers Hsp90 alpha (A, B), Hsp90 beta (C), flotillin-2 (D) and beta actin (E) were analyzed by Western blot in human samples isolated by UC and EQ techniques. All exosome markers were detected in pleural fluid exosomes isolated by either ultracentrifugation (UC) or Exoquick (EQ) solution, confirming that both of these protocols yield populations of purified exosomes. Since these markers are cellular proteins that are enriched in exosomes (ExoCarta, www.exocarta.org), these proteins were also expressed in cell lysates from BCBL1 PEL cells (denoted as “Cells”). As expected, the exosomal markers were absent in the supernatant fraction. Note, only 250 µl of pleural fluid was used as input for the Exoquick protocol and ∼35 mls pleural fluid was used as starting volume for the ultracentrifugation method. Sample were normalized by equal input volumes for each technique. (TIF) [file ppat.1003484.s006.tif]

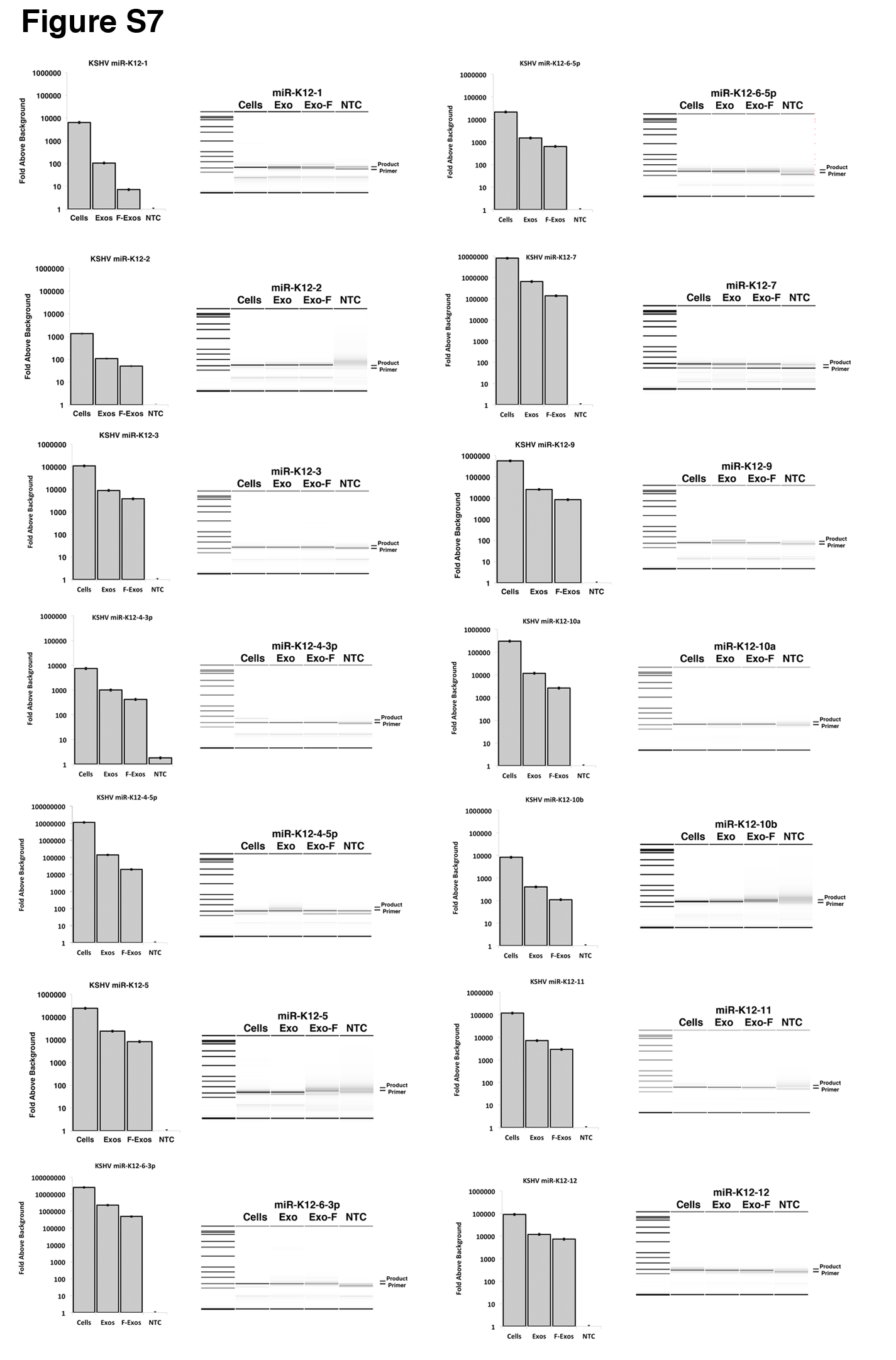

Supplement: Figure S7 — KSHV miRNAs are not significantly affected by filtering of exosome-enriched samples. Supernatants from latently infected BCBL1 cells were collected and exosomes were isolated using the ExoQuick method. Prior to ExoQuick enrichment, supernatants were passed through a 0.2 micron Whatman filter to decrease the amount of virus present in the sample. RNA from BCBL1 cells was used as a positive control for expression of KSHV miRNAs. PCR-grade water was used as input for the cDNA reaction for a no template control (NTC). Total RNA was isolated using Triazol and samples were normalized to equal amounts of total RNA input. Taqman microRNA assays (Life Technologies) for 14 KSHV miRNAs were used to determine levels of expression by qPCR, which are shown as fold above background for three technical replicates. qPCR products were diluted 1∶10 and run on a Caliper nanofluidics platform (similar to traditional gel electrophoresis). The KSHV miRNA products run at ∼60 bp with the primers and Taqman probe annealed. Cells, exosomes and filtered exosomes expressed KSHV miRNAs to similar levels. A shifted band consisting of the primer dimers can be seen in the NTC lanes. Abbreviations: Exo, exosomes; Exo-F, filtered exosomes; NTC, no template control. (TIF) [file ppat.1003484.s007.tif]

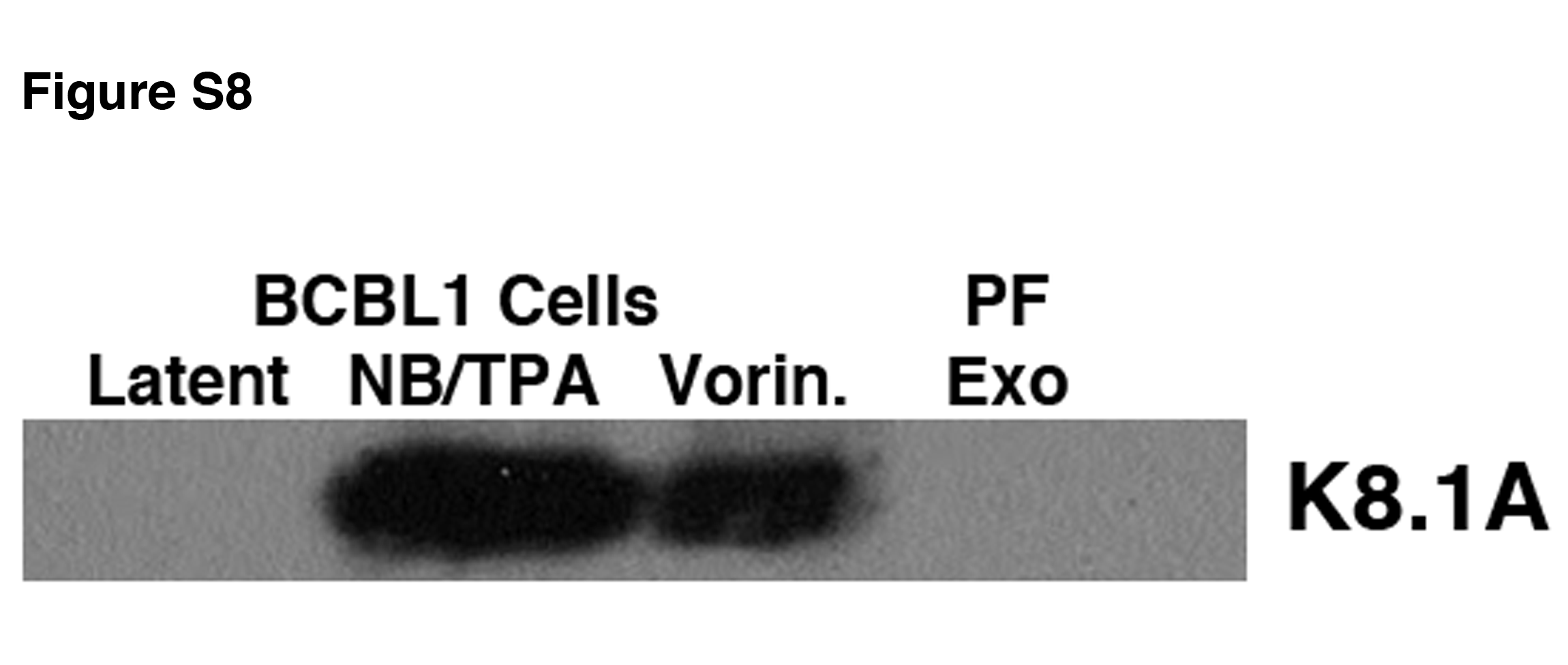

Supplement: Figure S8 — The structural protein K8.1 is not detected within exosome fractions. The BCBL1 PEL cell line was cultured and cells were reactivated with either 1.25 mM sodium butyrate (NaB) and 20 ng/ml TPA or 1.25 mM vorinostat. After 48 hours, latent and reactivated BCBL1 cells were collected. Pleural fluid-derived exosomes (PF exo) were isolated using ExoQuick and all samples were lysed in NP40 buffer containing proteinase (Sigma) and phosphatase inhibitors. Samples were loaded onto a 10% polyacrylamide-SDS gel and then transferred to a nitrocellulose membrane (Hybond). The membrane was blocked with 5% dry milk in TBST overnight and then incubated with primary K8.1 anti-mouse at 1∶100 followed by anti-mouse secondary antibody at 1∶5000. Blots were developed using Pierce ECL substrate kit and Blue Devil autoradiography film and a representative blot of three independent experiments is shown. K8.1 was only expressed in reactivated BCBL1 cells and was absent in both latently infected BCBL1 cells and PF-derived exosomes. (TIF) [file ppat.1003484.s008.tif]

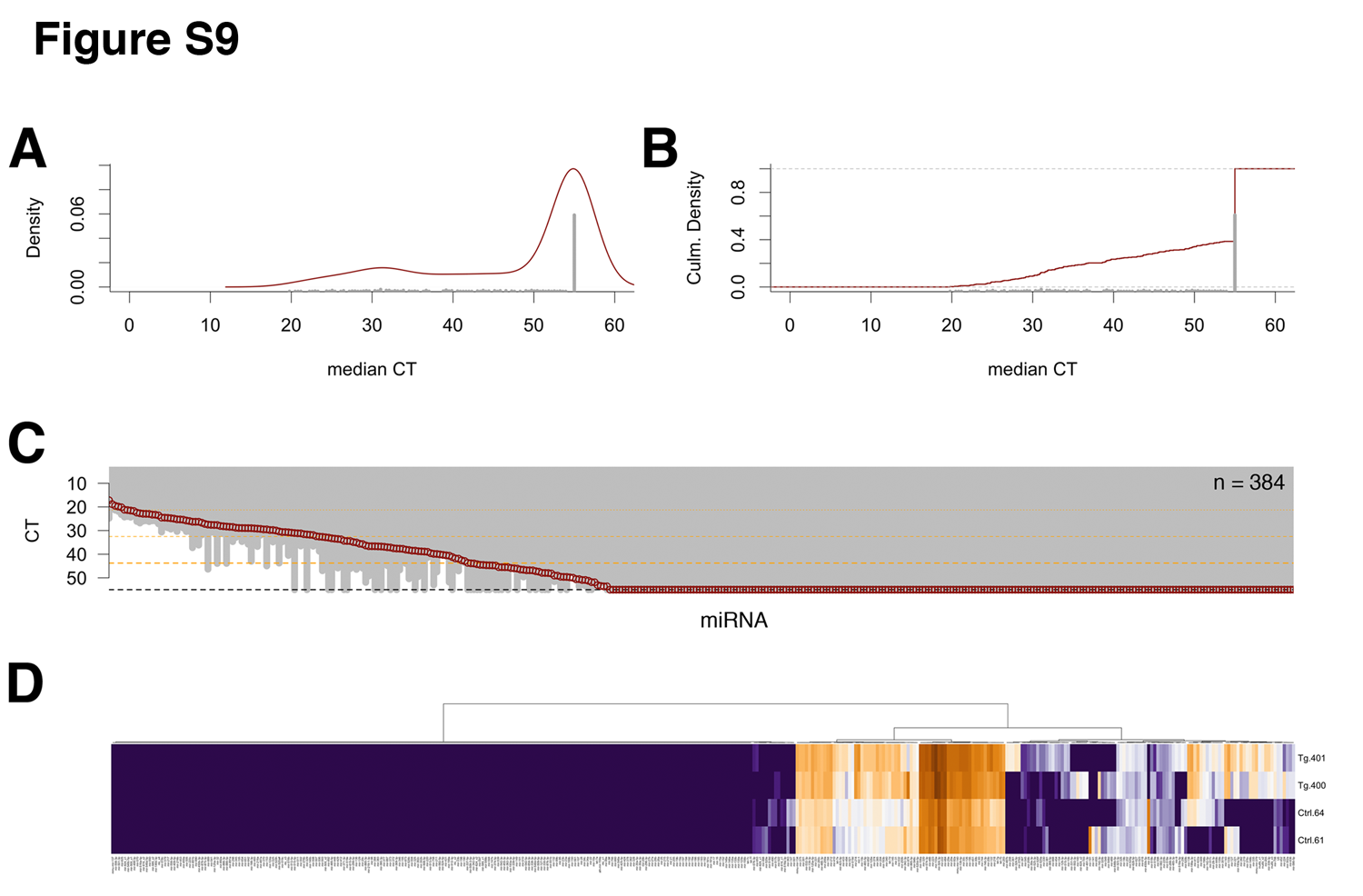

Supplement: Figure S9 — Latency locus transgenic mice express a distinct miRNA signature compared to control mice. Comparative analysis of exosomal miRNA profiles from transgenic mice encoding the KSHV latency locus (Tg400, Tg401) and control mice (Ctrl 61, Ctrl 64). Mouse serum (250 µl) from individual control and transgenic mice were used as input for the ExoQuick exosome isolation method. Following RNA isolation, cDNA was amplified and Taqman miRNA profiles of each sample were performed as described in the methods section. (A) Density distribution (red) and histogram (gray) of median CT of n: 384 miRNAs across all samples. (B) Cumulative density plot for the same data. (C) Waterfall plot of the median CT for each of the miRNAs in the transgenic mice is shown in red. White lines indicate median CT for control mouse samples. Yellow lines indicate 1, 2 and 3 standard deviations of the median CT of the positive sample, representing the expression percentile under the assumption of a normal distribution of the data. (D) Heatmap of CT values for all miRNAs in all samples (blue indicating absence, white intermediate and brown, highest levels of a given miRNA). Clustering was based on Ward's criteria and Manhattan distance metric. (TIF) [file ppat.1003484.s009.tif]

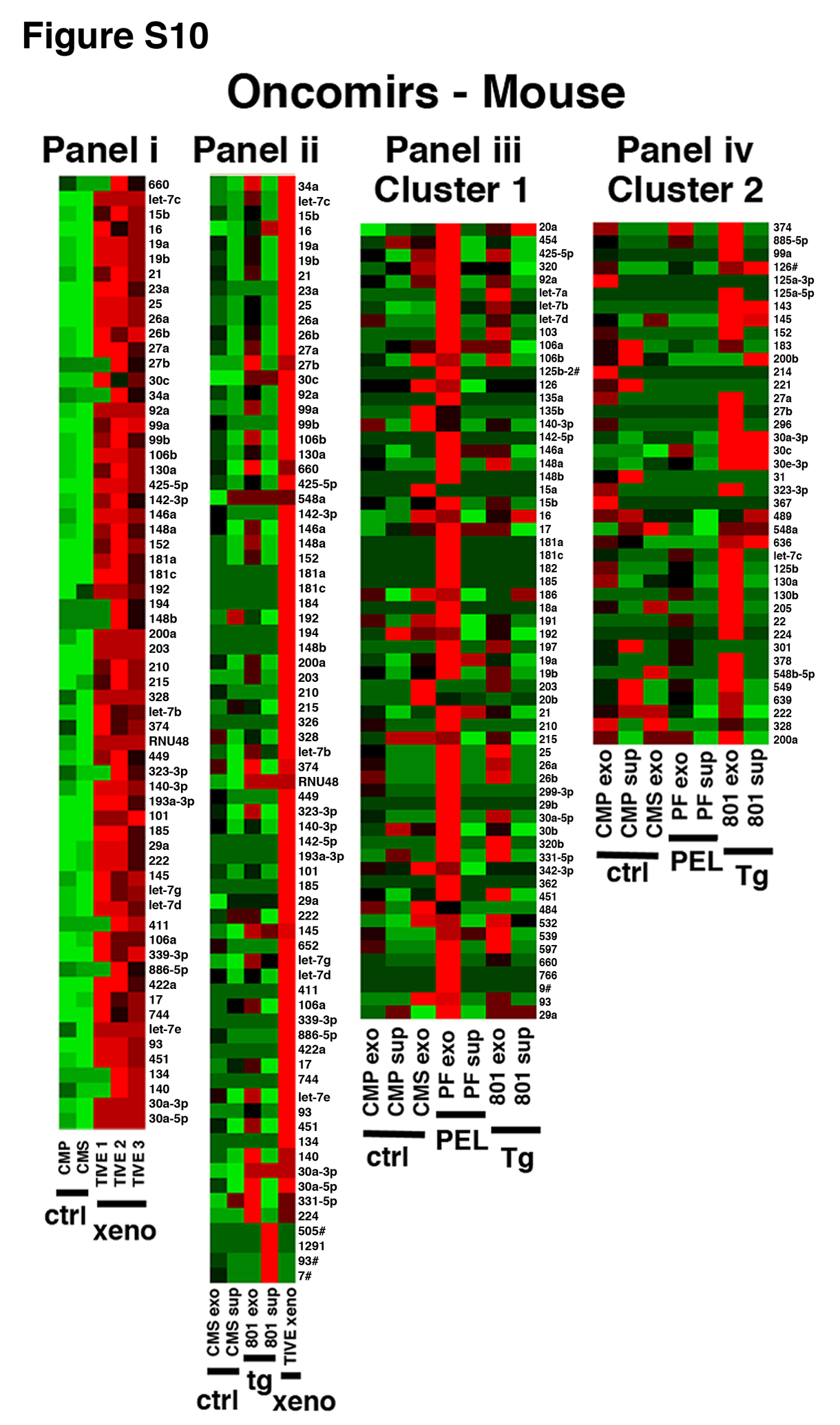

Supplement: Figure S10 — Profiling of oncomirs in transgenic and xenograft mouse models. Heatmaps reflective of unsupervised clustering analysis are shown for oncomirs in mouse samples. Oncomirs from mouse models are shown as a series of panels (i–iv). Panel i compares oncogenic miRNA expression between control mice and individual TIVE xenograft mice. Profiling data from the transgenic mouse model encoding the KSHV latency locus is shown in Panel ii with control mice and the average of the TIVE xenograft data. Panels iii and iv show two separate clusters of miRNA expression and compare control and transgenic mice to the primary human PEL pleural fluid cases. Abbreviations of mouse samples are as follows: CMP – control mouse plasma, CMS – control mouse serum, TIVE 1–3, individual TIVE xenograft mice, tg – 801 latency locus mouse model, PF – primary human PEL pleural fluid, exo – exosomes, sup – exosome-depleted supernatants. The names of each microRNA in the cluster are shown to the right of each heatmap. Red denotes high expression, green denotes low expression and black is basal or intermediate expression. (TIF) [file ppat.1003484.s010.tif]

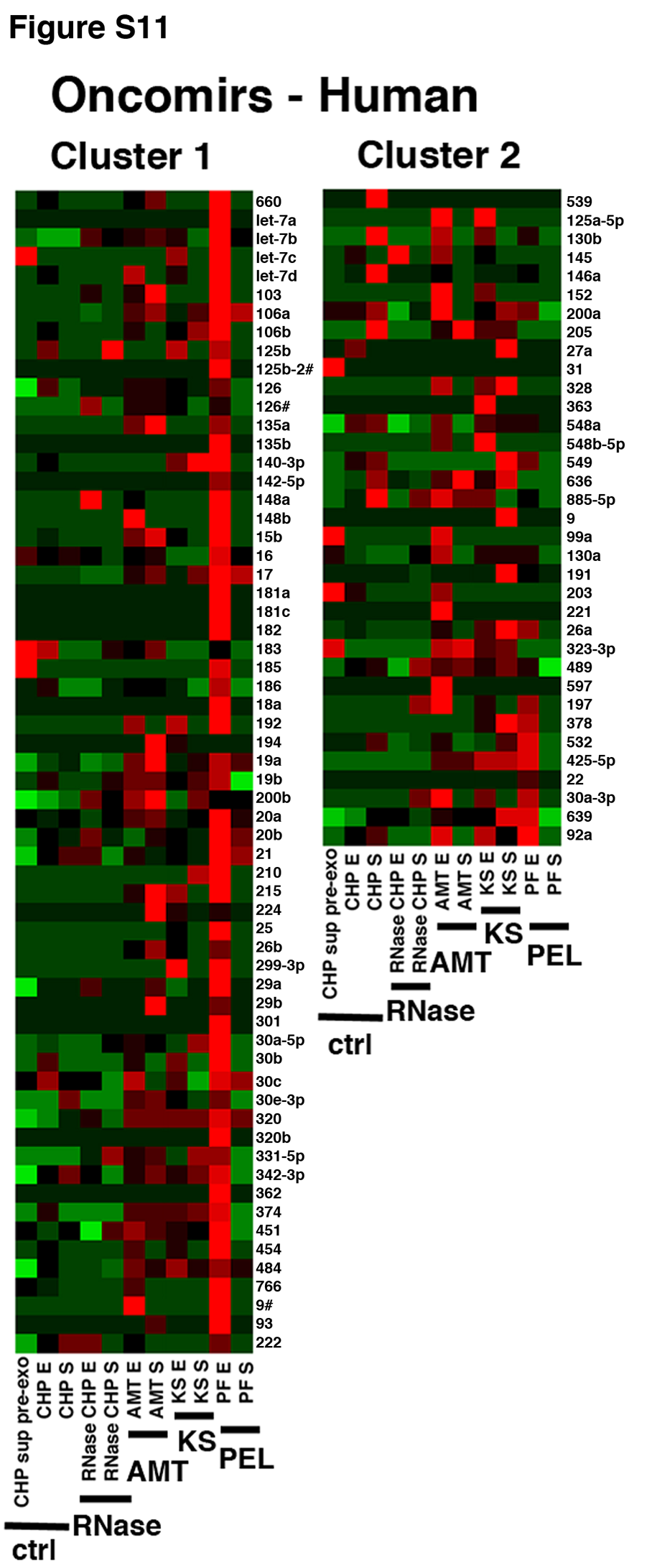

Supplement: Figure S11 — Profiling of oncomirs in human patient samples. Heatmaps reflective of unsupervised clustering analysis are shown for oncomirs in human samples. Oncomir expression in human samples is shown as two separate clusters of expression. For the human oncomiR heatmap, sample lanes from left to right are: CHP sup pre-exo - control human plasma supernatant pre-ExoQuick, CHP E – control human plasma exosomes, CHP S – control human plasma exosome-depleted supernatant, RNase CHP E – RNase-treated CHP exo, RNase CHP S – RNase-treated CHP sup, AMT E – AIDS Malignancy, non-KS exo, AMT S – AIDS Malignancy, non-KS sup, KS E – Kaposi's sarcoma exosomes, KS S – Kaposi's sarcoma exosome-depleted supernatant, PF E – pleural fluid exosomes, PF S – pleural fluid exosome-depleted supernatant. The names of each microRNA in the cluster are shown to the right of each heatmap. Red denotes high expression, green denotes low expression and black is basal or intermediate expression. (TIF) [file ppat.1003484.s011.tif]

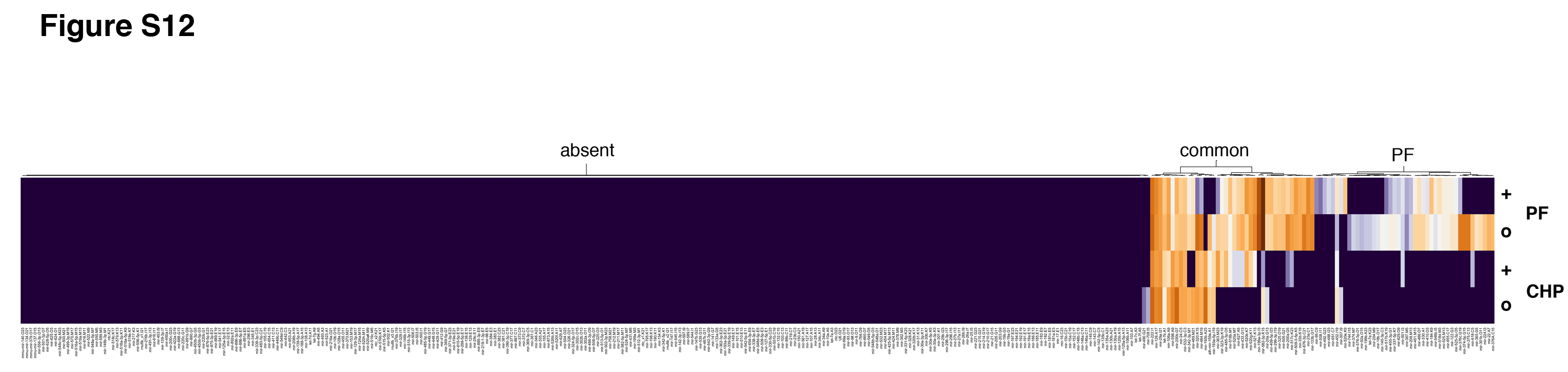

Supplement: Figure S12 — PF- and CHP-derived exosomal miRNA profiles respond similarly to RNase treatment. Exosomes were isolated using the ExoQuick solution and equal volumes (250 µl) of pleural fluid (PF) or control human plasma (CHP). Samples were either left alone (−) or treated with 25 U/ml RNase (Roche) for 30 minutes at 37°C (+). RNA was isolated from exosome-enriched samples and the miRNA expression profile was determined by qPCR using Taqman microRNA assays from human pool A v2.1 (Life Technologies). Heatmap of CT values for all miRNAs in all samples (blue indicating absence, white intermediate and brown, highest levels of a given miRNA). Clustering was based on Ward's criteria and Manhattan distance metric. The heatmap shows that the majority of miRNAs increased in PF-derived exosomes were not affected by RNase treatment. Similarly, the expression profile of control human plasma exosomes did not significantly change in response to RNase treatment. This demonstrates that different patient samples have similar responses to RNase treatment and that exosomal miRNAs are largely RNase-resistant. (TIF) [file ppat.1003484.s012.tif]

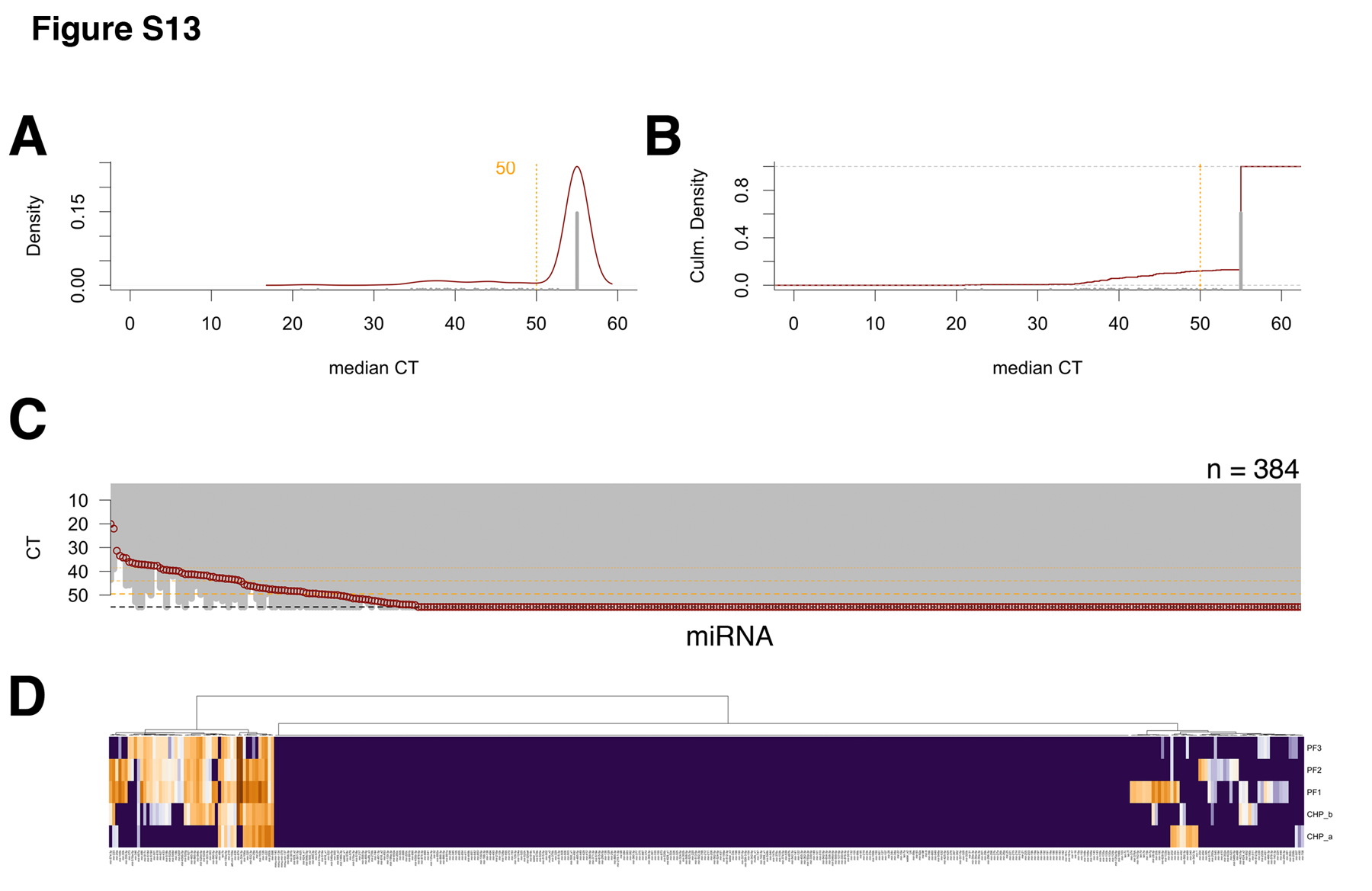

Supplement: Figure S13 — Profiling of individual PEL patients reveals insight into variability of miRNA profiles. Comparative analysis of exosomal miRNA profiles from individual PEL patient fluids (pleural fluid patients 1–3) and KS-free control fluids (CHP, control human plasma). Pleural fluid from individual PEL patients and control human plasma were used as input for the ExoQuick exosome isolation method. Following RNA isolation, cDNA was amplified and Taqman miRNA profiles of each sample were performed as described in the methods section. (A) Density distribution (red) and histogram (gray) of median CT of n: 384 miRNAs across all samples. Indicated in orange is the CT = 50 cutoff. (B) Cumulative density plot for the same data. (C) Waterfall plot of the median CT for each of the miRNAs in the PEL patient fluids (PF1-3) shown in red. White lines indicate median CT for KSHV negative samples (CHP a and b). Yellow lines indicate 1, 2 and 3 standard deviations of the median CT of the positive sample, representing the expression percentile under the assumption of a normal distribution of the data. (D) Heatmap of CT values for all miRNAs in all samples (blue indicating absence, white intermediate and brown, highest levels of a given miRNA). Clustering was based on Ward's criteria and Manhattan distance metric. MiRNAs were highly expressed in PF-derived exosomes compared to control exosomes. A large number of these PEL signature miRNAs were expressed in all three patients and could thus be novel biomarkers of PEL. Despite the inherent genetic variability among patients, we could identify multiple miRNAs that were expressed at high levels in all three PEL patients compared to controls. (TIF) [file ppat.1003484.s013.tif]

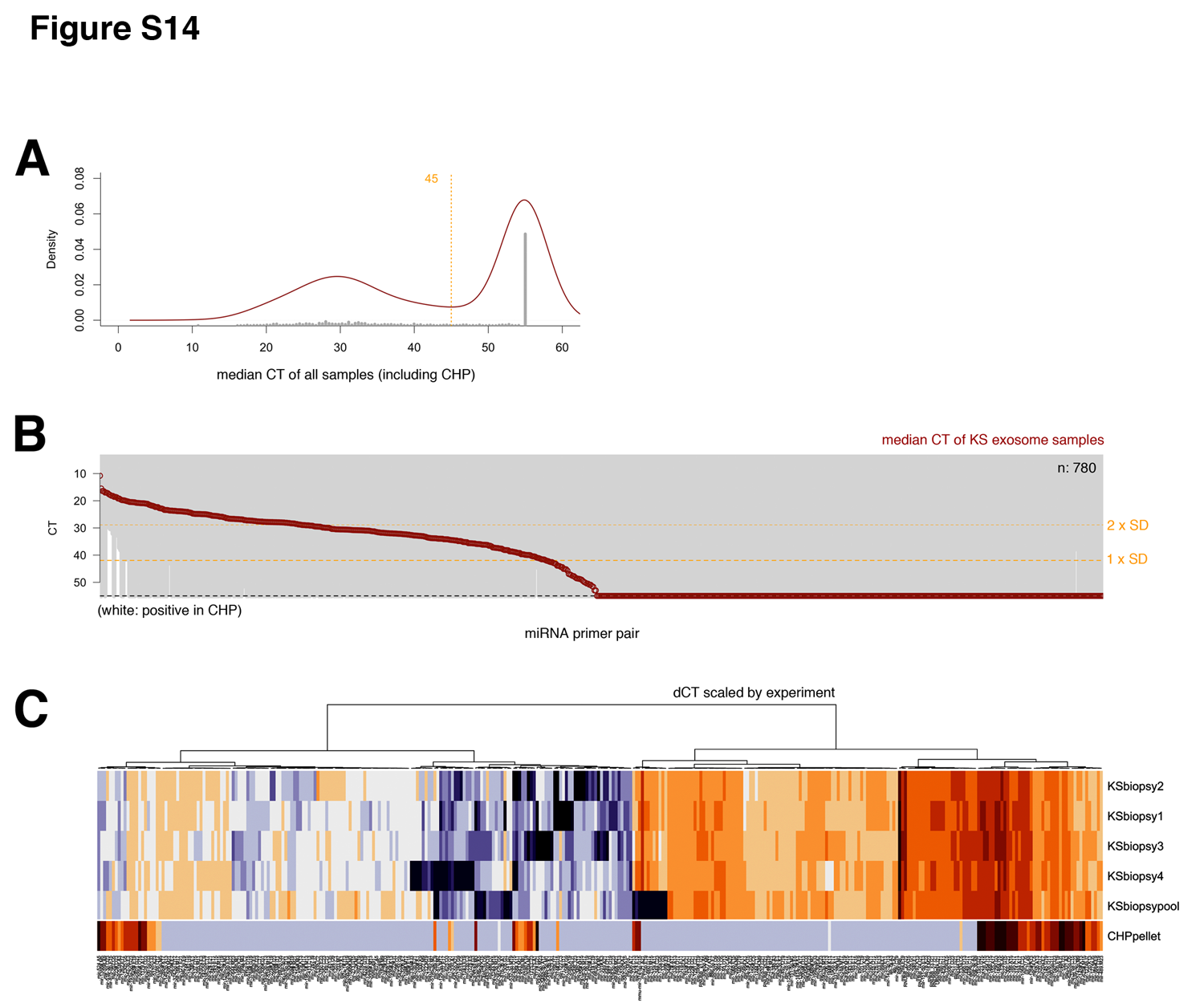

Supplement: Figure S14 — MiRNA profiling of KS biopsies and PBMCs from control human plasma. Comparative analysis of miRNA profiles from KS biopsies and PBMCs from control human plasma (CHP pellet). Four primary KS biopsies were pooled to obtain the KS biopsy pool sample. (A) Density distribution (red) and histogram (gray) of median CT of n: 384 miRNAs across all samples. Indicated in orange is the CT = 45 cutoff. (B) Waterfall plot of the median CT for each of the miRNAs in the KS biopsy samples shown in red. White lines indicate median CT for CHP pellet sample. Yellow lines indicate 1 and 2 standard deviations of the median CT of the positive sample, representing the expression percentile under the assumption of a normal distribution of the data. (D) Heatmap of CT values for all miRNAs in all samples (blue indicating absence, white intermediate and brown, highest levels of a given miRNA). Clustering was based on Ward's criteria and Manhattan distance metric. KS biopsies display a unique miRNA profile compared to CHP cells, with increased levels of several oncogenic miRNAs in all 4 KS biopsies (also seen in Figure 5). (TIF) [file ppat.1003484.s014.tif]

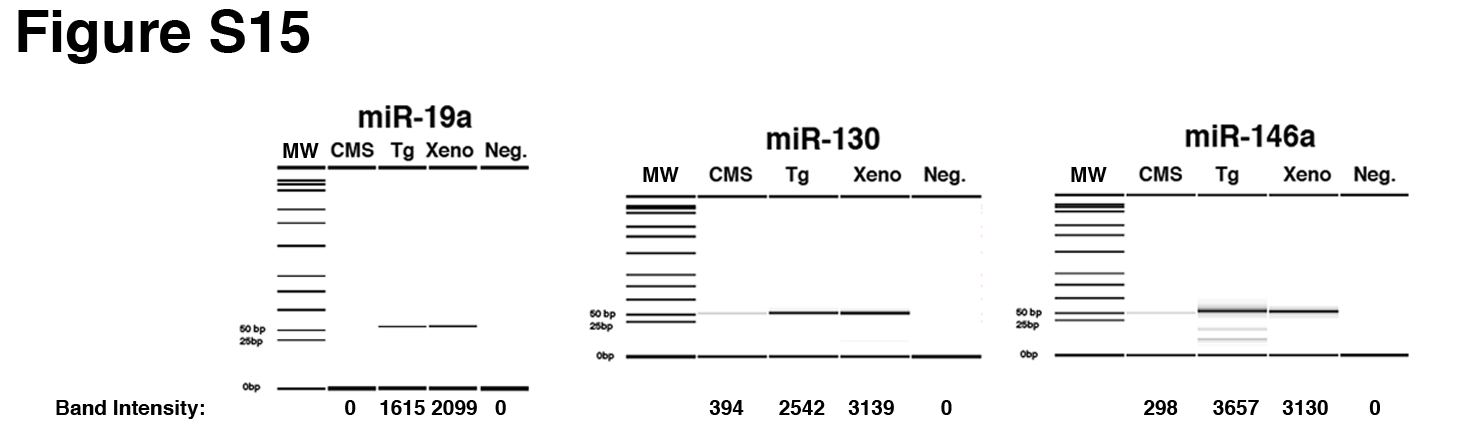

Supplement: Figure S15 — Caliper gel analysis of oncomirs induced in tumor mouse models. Gel electrophoresis analysis of qPCR products of known oncomirs that were upregulated in tumor mouse models in our profiling study. qPCR products were diluted 1∶5 with molecular grade water and run on the Caliper GX Labchip using the HTDNA 1K chip (Caliper Life Sciences, Hopkinton, Massachusetts). This chip functions analogous to a traditional agarose gel and shown is an image of the band migration. Induction of these microRNAs in tumor mouse models was confirmed by increased band intensity of the PCR product compared with control mouse serum (CMS). This confirms the specificity of the assay and that key oncogenic miRNAs are enriched in exosomes from our mouse models of KS. Abbreviations: CMS, control mouse serum, Tg, 801 latency locus transgenic mouse model, Xeno, SLK xenograft mouse model, Neg., no template control (PCR-grade water), MW (molecular weight ladder). The band intensity was measured using Image J gel analysis (rsbweb.nih.gov/ij/) and is shown in arbitrary units of band intensity. (TIF) [file ppat.1003484.s015.tif]

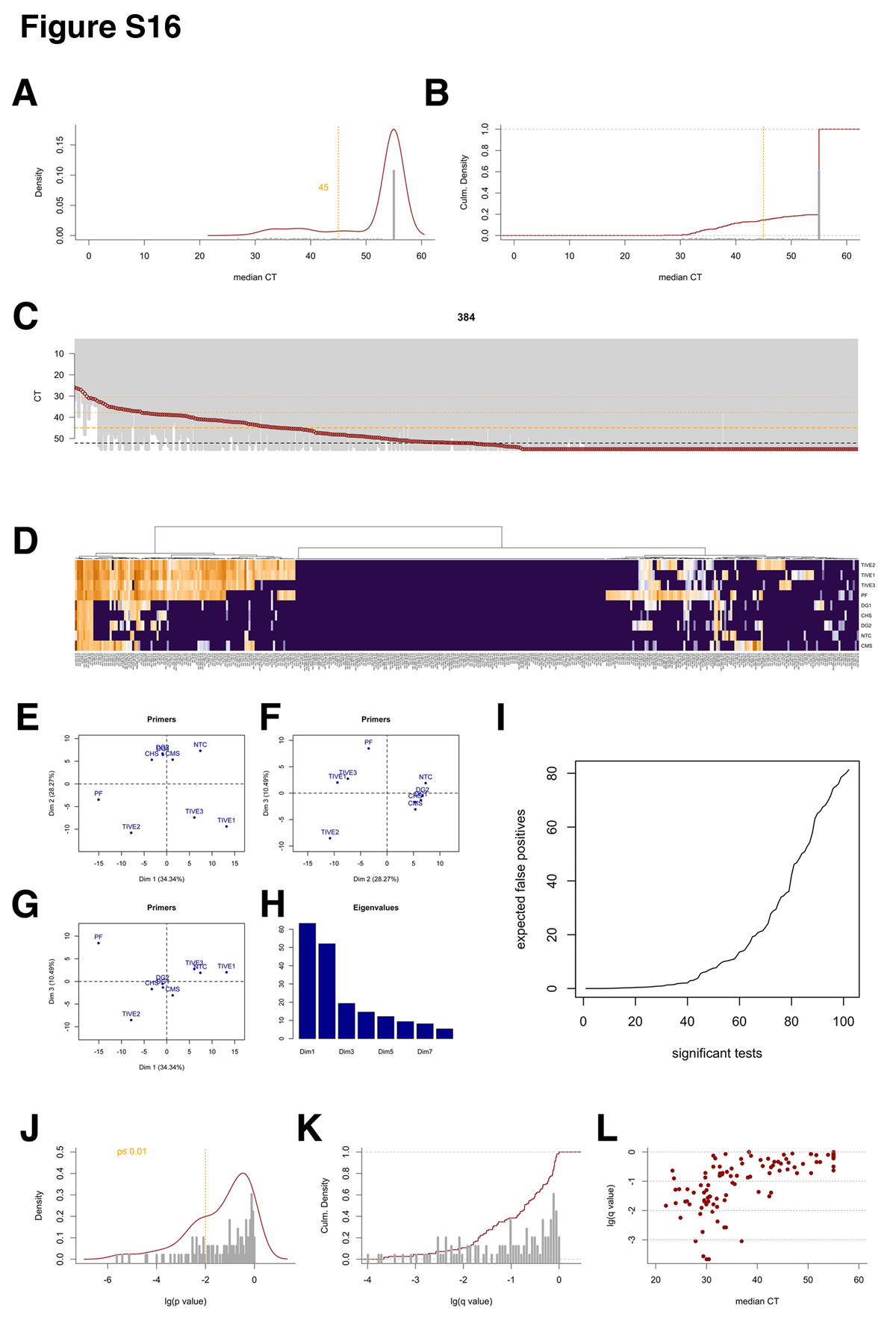

Supplement: Figure S16 — Circulating microRNA profiles of TIVE xenograft model and KS case studies. Comparative analysis of exosomal miRNA profiles from human (PF, DG1, DG2), murine xenograft models (TIVE1, TIVE2, TIVE3) and KS-free control fluids (CHS, human serum and CMS, mouse serum) and non-template control (NTC). (A) Density distribution (red) and histogram (gray) of median CT of n: 384 miRNAs across all samples. Indicated in orange is the CT = 45 cutoff. (B) Cumulative density plot for the same data. (C) Waterfall plot of the median CT for each of the miRNAs in the KSHV positive samples (TIVE1, TIVE2, TIVE3, PF, DG1, DG2) shown in red. White lines indicate median CT for KSHV negative samples (CMS, CHS, NTC). Yellow lines indicate 1, 2 and 3 standard deviations of the median CT of the positive sample, representing 68.3, 94.5 and 97.7 percentile under the assumption of a normal distribution of the data. (D) Heatmap of CT values for all miRNAs in all samples (blue indicating absence, white intermediate and brown, highest levels of a given miRNA). Clustering was based on Ward's criteria and Manhattan distance metric. (E–G) Plots comparing the first three dimensions of the principal component analysis (PCA) using n = 184 highly changed miRNAs. These were selected based on a median absolute deviation (m.a.d.) >4 across all samples. These demonstrate clustering of the control samples and KSHV positive samples. (H) Histogram of the Eigenvalues of the dimensions of the PCA analysis. Higher Eigenvalues indicate a greater contribution of a given distribution to the data variability. (I) Expected false positive rate on the vertical axis compared to the number of significant tests on the horizontal axis. This was obtained by calculating p values of an unpaired, two-sided t-test, allowing for unequal variance for comparison of the positive samples (TIVE1, TIVE2, TIVE3, PF) to negative samples (CHS, CMS, NTC) for each primer followed by adjustment for multiple comparisons using q-value method. Less [file ppat.1003484.s016.tif]

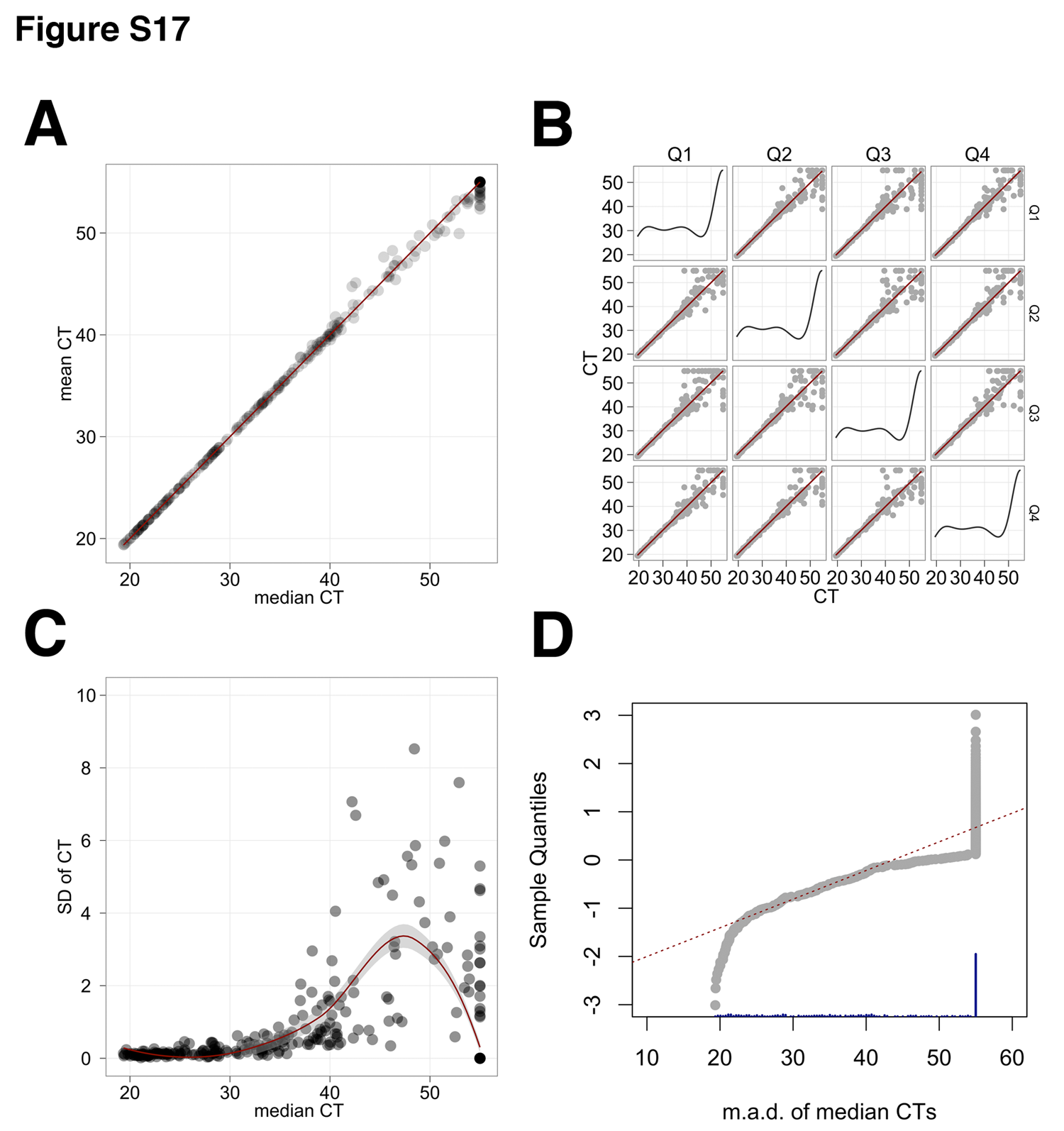

Supplement: Figure S17 — Technical replicates of microRNA data reveal little variation using an automated liquid-handling system. Technical replicates of microRNA expression data were obtained using a Tecan Freedom Evo liquid-handling system. (A) Comparison of the median CTs shows linear correlation between technical replicates. (B) For each 384-well plate, 96 primers are run against 4 samples in each of 4 quadrants. In this test set, the same sample was run in each of 4 quadrants. Analysis of the microRNA data from each quadrant was conducted and revealed the absence of any quadrant bias. Furthermore, it shows tight correlation between technical replicates for CTs up to ∼45. (C) The distribution of the median CTs are plotted against the standard deviation of the CT. This recapitulates that there is little variation in technical replicates at lower CTs (up to ∼40). However, for CTs between 45 and 55, there can be highly variable deviation from the median. (D) Q-Q plot showing that the microRNA data of technical replicates follows a normal distribution, which is represented by the dotted line. (TIF) [file ppat.1003484.s017.tif]
