# Supplementary material for: Systemically Circulating Viral and Tumor-Derived MicroRNAs in KSHV-Associated Malignancies
Source: PLoS Pathog. 2013 Jul 18;9(7):e1003484. doi: 10.1371/journal.ppat.1003484 (PMC3715412; doi:10.1371/journal.ppat.1003484)
Supplement: Table S1 — Clinical human and mouse model samples used in exosome study. Sample groups used for miRNA profiling are shown along with the number of samples pooled for each group (N). (DOCX) [file ppat.1003484.s018.docx]

**Table S1 – Clinical human and mouse model samples used in exosome study.**

| **Samples** | ***N (Samples per group)*** |
| --- | --- |
| Control human plasma | 4 |
| AMT | 4 |
| AMT KS | 4 |
| KS Malawi | 4 |
| Pleural Fluid | 2 |
| KS biopsy A | 1 |
| KS biopsy B | 1 |
| KS biopsy C | 1 |
| KS biopsy D | 1 |
| KS biopsy E | 1 |
| KS biopsy F | 1 |
| KS biopsy pool | 4 |
| KS case study pt. 1 | 1 |
| KS case study pt. 2 | 1 |
| Control plasma | 10 |
| Control serum | 5 |
| 801 Tg serum | 5 |
| TIVE xenograft serum | 5 |

Sample groups used for microRNA profiling are shown along with the number of samples pooled for each group (N).
