# Supplementary material for: Systemically Circulating Viral and Tumor-Derived MicroRNAs in KSHV-Associated Malignancies
Source: PLoS Pathog. 2013 Jul 18;9(7):e1003484. doi: 10.1371/journal.ppat.1003484 (PMC3715412; doi:10.1371/journal.ppat.1003484)
Supplement: Table S2 — List of microRNAs included in the oncomiR and tumor suppressor array. The Taqman microRNA primers used in the oncomiR and tumor suppressor array are listed. These ∼150 microRNAs include known oncomiRs and tumor suppressor microRNAs according to published literature and references are shown for each microRNA. A subset of these microRNAs were cross-referenced to the Quantimir cancer array (System Biosciences) for confirmation of oncomir status. MicroRNAs previously found to be altered in KSHV-associated malignancies are also noted with an “x”. (DOCX) [file ppat.1003484.s019.docx]

**Table S2**

**List of microRNAs Profiled in the Oncogenic**

**and Tumor Suppressor Array**

| **Primer Name** | **References** | **Altered by KSHV** |
| --- | --- | --- |
| let-7a | Akao et al 2006, Sampson et al 2007, Long et al 2009, Liu et al 2012 | x |
| let-7b | Xu et al 2012, Chen et al 2012, Schultz et al 2008 | x |
| let-7c | Pelosi et al 2012, Nadiminty et al 2012, Han et al 2012 | x |
| let-7d | Childs et al 2009, Li et al 2009, Chang et al 2011 | x |
| let-7e | Wang et al 2012, Mitra et al 2011, Buechner et al 2011 | x |
| let-7f | Liang et al 2011, Shibahara et al 2012 | x |
| let-7g | Lan et al 2011, Ji et al 2010, Hu et al 2013, Qian et al 2011, Arora et al 2011, Park et al 2010 | x |
| let-7i | Yang et al 2008, Liu et al 2012, Zhang et al 2012 | x |
| mir-101 | Sachdeva et al 2011, Wang et al 2010, Buechner et al 2011, Strillacci et al 2013, Cho et al 2011, He et al 2012, Wang et al 2012 |  |
| mir-106a | Zhi et al 2010, Yang et al 2011, Wang et al 2012, Feng et al 2012 | x |
| mir-106b | Kan et al 2009, Poliseno et al 2010, Cai et al 2011, Zhao et al 2012, Shen et al 2013 |  |
| mir-1226 | Jin et al 2010 |  |
| mir-1233 | Wulfken et al 2011 |  |
| mir-1244 | White et al 2010 |  |
| mir-1254 | Foss et al 2011 |  |
| mir-1255b | Hidaka et al 2012 |  |
| mir-125b | Chaudhuri et al 2012, Kim et al 2012, Tang et al 2012, Xu et al 2012, Kappelmann et al 2012, Jia et al 2012, Kim et al 2012, Cui et al 2012 | x |
| mir-1271 | Nurul-Syakima et al 2011, Maurel et al 2013 |  |
| mir-1290 | Endo et al 2012, Wu et al 2013, Price et al 2012 |  |
| mir-130a | Chen et al 2008, Xu et al 2012, Boll et al 2013 |  |
| mir-130b | Yeung et al 2008, Lai et al 2010, Ma et al 2010, Suresh et al 2011, Yang et al 2012, Dong et al 2012 |  |
| mir-134 | Boominathan 2010, Li et al 2012 |  |
| mir-135a | Nagel et al 2008, Navarro et al 2009, Wu et al 2012, Chen et al 2012, Yamada et al 2012 |  |
| mir-135b | Matsuyama et al 2011, Xu et al 2012, Zhang et al 2013 |  |
| mir-140 | O'Hara et al 2009, Song et al 2009, Zhang et al 2012 | x |
| mir-140-3p | Lionetti et al 2009, Piepoli et al 2012 |  |
| mir-142-3p | Wu et al 2011, Wang et al 2012, Lv et al 2012, Wang et al 2012, Lei et al 2012 | x |
| mir-142-5p | Sempere et al 2009, Zhang et al 2011 |  |
| mir-144 | Kalimutho et al 1022, Liu et al 2012, Iwaya et al 2012, Zhang et al 2012 |  |
| mir-145 | Sachdeva et al 2009, Sachdeva et al 2010, Chiyomaru et al 2010, Zhang et al 2011, Kano et al 2010, Xu et al 2012, Shi et al 2012, Sachdeva et al 2012, Zou et al 2012, Speranza et al 2012 | x |
| mir-146a | Li et al 2012, Labbaye et al 2012, Paik et al 2011, Hou et al 2012, Boominathan 2010, Hurst et al 2009 | x |
| mir-148a | Lujambio et al 2008, Fujita et al 2010, Zheng et al 2011 |  |
| mir-148b | Song et al 2011, Song et al 2012, Zhao et al 2013 |  |
| mir-151-5p | Niemoeller et al 2011, Krell et al 2012, Presneau et al 2012 |  |
| mir-152 | Chen et al 2010, Tsuruta et al 2011, Zhou et al 2012, Woo et al 2012 | x |
| mir-15a | Calin et al 2008, Ofir et al 2011, Gao et al 2011, Sun et al 2012 | x |
| mir-15b | Satzger et al 2010, Ofir et al 2011 |  |
| mir-16 | Calin et al 2008, Bonci et al 2008, Gatt et al 2010, Ofir et al 2011, Gao et al 2011, Rivas et al 2012, Sun et al 2012 | x |
| mir-17 | He et al 2005, Dews et al 2006, Italiano et al 2012, Concepcion et al 2012, Wei et al 2012, Kandalam et al 2012 |  |
| mir-181a | Shi et al 2008, Shin et al 2011, Zhang et al 2012, Taylor et al 2013 |  |
| mir-181c | Xue et al 2011, Lakomy et al 2011, Jones et al 2012 |  |
| mir-183 | Wang et al 2008, Li et al 2010, Lowery et al 2010, Sarver et al 2010, Mihelich et al 2011, Weeraratne et al 2012, Li et al 2012, Zhu et al 2012, Tang et al 2012 |  |
| mir-184 | Wong et al 2008, Yu et al 2008, Wong et al 2009, Foley et al 2010, Tivnan et al 2010 |  |
| mir-185 | Imam et al 2010, Liu et al 2011, Akcakaya et al 2011, Liao et al 2011, Zhang et al 2011, Tang et al 2012, Xiang et al 2013 |  |
| mir-18a | Tsang et al 2009, Doebele et al 2010, Tao et al 2012, Li et al 2012, Luo et al 2012 | x |
| mir-192 | Braun et al 2008, Pichiorri et al 2010, Feng et al 2011, Chiang et al 2012 |  |
| mir-193a | Gao et al 2011, Iliopoulos et al 2011, Heller et al 2012 | x |
| mir-193b | Li et al 2009, Unno et al 2009, Rauhala et al 2010, Chen et al 2010, Xu et al 2010, Gao et al 2011, Chen et al 2011, Hu et al 2012, Xie et al 2012, Lenarduzzi et al 2013 |  |
| mir-194 | Song et al 2012, Dong et al 2011, Sundaram et al 2011, Senanayake et al 2012, Le et al 2012, Chiang et al 2012 |  |
| mir-197 | Du et al 2009, Hamada et al 2012 |  |
| mir-19a | Pezzolesi et al 2008, Liu et al 2011, Li et al 2012, Xu et al 2012, Zhang et al 2012 | x |
| mir-19b | Li et al 2012, Kurokawa et al 2012, Xu et al 2012 | x |
| mir-200a | Saydam et al 2009, Xia et al 2010, Elson-Schwab et al 2010, Eades et al 2011, Snowdon et al 2011, Mateescu et al 2011, Su et al 2012, Yu et al 2013 |  |
| mir-203 | Bueno et al 2008, Furuta et al 2010, Viticchie et al 2011, Bian et al 2012, Boll et al 2013, Zhang et al 2011, Moes et al 2012, Takeshita et al 2012, Jin et al 2013, Qu et al 2013 |  |
| mir-21 | Gabriely et al 2008, Zhang et al 2008, Talotta et al 2009, Selcuklu et al 2009, Ziyan et al 2011, Medina et al 2010, Lou et al 2010, Xiong et al 2012, Ng et al 2012, Reis et al 2012, Zhang et al 2012, Stik et al 2013 | x |
| mir-210 | Camps et al 2008, Zhang et al 2009, Huang et al 2009, Gee et al 2010, Tsuchiya et al 2011, Nakada et al 2011, Rothe et al 2011, Yang et al 2012, Noman et al 2012, Xiong et al 2012, He et al 2013 |  |
| mir-215 | Braun et al 2008, Georges et al 2008, Karaayvaz et al 2011, White et al 2011, Senanayake et al 2012 |  |
| mir-22 | Bar et al 2010, Xiong et al 2010, Xu et al 2011, Tsuchiya et al 2011, Alvarez-Diaz et al 2012, Li et al 2012, Ling et al 2012 |  |
| mir-221 | Galardi et al 2007, le Sage et al 2007, Zhang et al 2009, Pineau et al 2010, Chun-Zhi et al 2010, Wu et al 2011, Garofalo et al 2012 | x |
| mir-222 | Galardi et al 2007, le Sage et al 2007, O'Hara et al 2009, Zhang et al 2009, Sredni et al 2010, Dentelli et al 2010, Chun-Zhi et al 2010,Garofalo et al 2012 | x |
| mir-224 | Ladeiro et al 2008, Huang et al 2012, Li et al 2010, Zhang et al 2012 |  |
| mir-23a | Kong et al 2010, Zhu et al 2010, Jahid et al 2012, Wang et al 2012, Cao et al 2012, Tan et al 2012 |  |
| mir-23b | Gao et al 2009, Au Yeung et al 2011, Chen et al 2012, Majid et al 2012, Zaman et al 2012, Jin et al 2013 |  |
| mir-25 | Kan et al 2009, Li et al 2009, Poliseno et al 2010, Razumilava et al 2012, Zhang et al 2012, Xu et al 2012 |  |
| mir-26a | Sander et al 2008, Huse et al 2009, Lu et al 2011, Chen et al 2011, Zhu et al 2012, Liu et al 2012, Reuland et al 2012, Zhu et al 2013 |  |
| mir-26b | Palumbo et al 2012, Zhu et al 2012, Liu et al 2012 |  |
| mir-27a | Mertens-Talcott et al 2007, Liu et al 2009, Ma et al 2010, Wang et al 2011, Zhao et al 2011, Lerner et al 2011, Spruck 2011, Feng et al 2012 |  |
| mir-27b | Kuehbacher et al 2008, Jin et al 2013, Lee et al 2012, Wang et al 2009 |  |
| mir-29a | Muniyappa et al 2009, Mott et al 2010, Eyholzer et al 2010, Kong et al 2011, Teichler et al 2011, Cui et al 2011, Weissmann-Brenner et al 2012, Fabbri et al 2012, Schmitt et al 2012, Wang et al 2013 |  |
| mir-29b | Mott et al 2007, Mott et al 2010, Wang et al 2011, Fang et al 2011, Zhang et al 2011, Ru et al 2012, Schmitt et al 2012, Chou et al 2013, Wang et al 2013 |  |
| mir-29c | Sengupta et al 2008, Wang et al 2011, Schmitt et al 2012, Liu et al 2013, Presnau et al 2012, Wang et al 2013 |  |
| mir-302b | Lee et al 2008, Borgdorff et al 2010, Subramanyam et al 2011, Yadav et al 2011 |  |
| mir-302c | Lowery et al 2009, Yoshimoto et al 2011, Dolezalove et al 2012 |  |
| mir-30a-3p | Zhu et al 2009, Kumarswamy et al 2012, Yu et al 2012, Cheng et al 2012, Tang et al 2012, Bridge et al 2012 |  |
| mir-30a-5p | Zhu et al 2009, Kumarswamy et al 2012, Baraniskin et al 2012, Yu et al 2012, Cheng et al 2012, Tang et al 2012, Bridge et al 2012 |  |
| mir-30c | Zhou et al 2012, Tanic et al 2012, Bridge et al 2012, Bockhorn et al 2013, Kim et al 2013 | x |
| mir-31 | Valastyan et al 2009, Liu et al 2010, Valastyan et al 2010, Cottonham et al 2010, Aprelikova et al 2010, Augoff et al 2011, Hua et al 2012, Laurila et al 2012, Li et al 2012, Yang et al 2012, Fuse et al 2012, Cekaite et al 2012, Lin et al 2013, Xu et al 2013, Sun et al 2013, Koerner et al 2013 | x |
| mir-320b | Wulfken et al 2011 |  |
| mir-323-3p | Qiu et al 2013 |  |
| mir-326 | Tang et al 2009, Liang et al 2010, Kefas et al 2009, Kefas et al 2010, Valencia et al 2013, Qiu et al 2013 |  |
| mir-328 | Pan et al 2009, Eiring et al 2010, Li et al 2010, Li et al 2011, Arora et al 2011, Xu et al 2012, Rutnam et al 2012 |  |
| mir-329 | Qiu et al 2013 |  |
| mir-331 | Feng et al 2011, Guo et al 2010 |  |
| mir-335 | Scarola et al 2010, Png et al 2011, Shu et al 2011, Heyn et al 2011, Xu et al 2012, Zhang et al 201, Shu et al 2012, Lynch et al 2012, Yan et al 2012, Dohi et al 2013 |  |
| mir-339 | Ueda et al 2009, Okada et al 2010 |  |
| mir-346 | Weber et al 2006, Tsai et al 2009 |  |
| mir-34a | Welch et al 2007, Raver-Shapira et al 2007, Chang et al 2007, Tazawa et al 2007, Li et al 2012, Kumar et al 2012, Genovese et al 2012, Rizzo et al 2012, Ahn et al 2012, Mandke et al 2012, Kasinski et al 2012, Yang et al 2012, Li et al 2012, Zhao et al 2013, Pang et al 2013 | x |
| mir-34c | Corney et al 2007, He et al 2007, Kumamoto et al 2008, Cai et al 2010, Cannell et al 2010, Lopez et al 2011, Chim et al 2011, Heinemann et al 2012, Yang et al 2012 |  |
| mir-361 | Roth et al 2012, Kanitz et al 2012, Dal Monte et al 2013 |  |
| mir-362 | Chan et al 2011 |  |
| mir-363 | Wald et al 2011, Beltran et al 2011, Sun et al 2012 |  |
| mir-367 | Li et al 2009, Kuo et al 2012, Campayo et al 2012 |  |
| mir-369 | Miyoshi et al 2011 |  |
| mir-373 | Voorhoeve et al 2006, Huang et al 2008, Yang et al 2009, Lee et al 2009, Wu et al 2011, Tanaka et al 2011, Liu et al 2012, Zhou et al 2012, Keklikoglou et al 2012, Golestaneh et al 2012, Chen et al 2012, Chen et al 2013 |  |
| mir-374 | Miko et al 2009 |  |
| mir-376a | Liu et al 2010, Zheng et al 2012, Zehavi et al 2012, Choudhury et al 2012 | x |
| mir-376c | Ye et al 2011, Duan et al 2011, Song et al 2012, Zehavi et al 2012 |  |
| mir-377 | Lowery et al 2009, Melkamu et al 2010 |  |
| mir-378 | Lee et al 2007, Eichner et al 2010, Feng et al 2011, Chen et al 2012, Hauser et al 2012, Deng et al 2013 |  |
| mir-380-5p | Swarbrick et al 2010 |  |
| mir-411 | Skalsky et al 2011, van Schooneveld et al 2012, Schultz et al 2012 |  |
| mir-412 | Gao et al 2011 |  |
| mir-422a | Faltejskova et al 2012 |  |
| mir-425 | Hummel et al 2011 |  |
| mir-432 | D'Angelo et al 2012, Huang et al 2012 |  |
| mir-449 | Lize et al 2010, Bou Kheir et al 2011, Lize et al 2011, Buurman et al 2012 |  |
| mir-451 | Zhu et al 2008, Gal et al 2008, Bandres et al 2009, Godlewski et al 2010, Nan et al 2010, Wang et al 2011, Li et al 2011, Lopotova et al 2011, Bergamaschi et al 2012, Bitarte et al 2011, Tian et al 2012, Kim et al 2011, Redova et al 2012 |  |
| mir-485 | Kim et al 2010, Costa et al 2011, Yang et al 2013 |  |
| mir-487a | Wang et al 2010 |  |
| mir-487b | Gattolliat et al 2011 |  |
| mir-497 | Zhu et al 2012, Li et al 2011, Poell et al 2012, Shen et al 2012, Guo et al 2012 |  |
| mir-500 | Yamamoto et al 2009, Zhu et al 2012 |  |
| mir-505 | Yamamoto et al 2011, Zhu et al 2011 |  |
| mir-518a-5p | Cheung et al 2012 |  |
| mir-518c | Wang et al 2010, Zhao et al 2009 |  |
| mir-518e | Wang et al 2012 |  |
| mir-519c | To et al 2009, Cha et al 2010, Li et al 2011, Hummel et al 2011 |  |
| mir-539 | Lee et al 2011, Thorns et al 2012, Hu et al 2012 |  |
| mir-548a-h | Liang et al 2012 |  |
| mir-549 | Hamfjord et al 2012 |  |
| mir-582-5p | Guled et al 2009, Uchino et al 2013 |  |
| mir-596 | Costa et al 2011, Endo et al 2013 |  |
| mir-601 | Yao et al 2009, Ohdaira et al 2009, Wang et al 2012, Yang et al 2013, Scheffer et al 2012 |  |
| mir-623 | Belian et al 2010, Xiao et al 2012 |  |
| mir-625 | Wang et al 2012, Roth et al 2012 |  |
| mir-633 | Xu et al 2011, Bae et al 2012 |  |
| mir-639 | Scheffer et al 2012 |  |
| mir-645 | Shih et al 2011 |  |
| mir-650 | Zhang et al 2010, Mraz et al 2012, Zeng et al 2013 |  |
| mir-652 | Roderburg et al 2012, Gaedcke et al 2012 |  |
| mir-657 | Patnaik et al 2010, Zhang et al 2012 |  |
| mir-660 | Zhu et al 2012, Ferrer et al 2013 |  |
| mir-663b | Ralfkiaer et al 2011, Ragusa et al 2012 |  |
| mir-675 | Tsang et al 2010, Dudek et al 2010, Schmitz et al 2011, Keniry et al 2012, Gao et al 2012 |  |
| mir-7 | Kefas et al 2008, Reddy et al 2008, Jiang et al 2010, Chou et al 2010, Xiong et al 2011, Wu et al 2011, Fang et al 2012, Kong et al 2012, Xu et al 2013, Giles et al 2013, Zhang et al 2012, Masuda et al 2012, Wang et al 2013 |  |
| mir-708 | Saini et al 2011, Saini et al 2012, Jang et al 2012, Robin et al 2012, Ryu et al 2013 |  |
| mir-720 | Ragusa et al 2012, Shinozuka et al 2013 |  |
| mir-744 | Nurul-Syakima et al 2011, Martin et al 2011, Huang et al 2012, Song et al 2012 |  |
| mir-766 | Hummel et al 2011 |  |
| mir-874 | Nohata et al 2011 |  |
| mir-886 | Lee et al 2011, Kunkeaw et al 2012, Jeon et al 2012 |  |
| mir-9 | Nass et al 2009, Ma et al 2010, Zhu et al 2012, Schraivogel et al 2011, Leucci et al 2012, Krell et al 2012, Zhang et al 2012, Selcuklu et al 2012, Zhuang et al 2012, Liu et al 2012, Cekaite et al 2012, Lu et al 2012, Gao et al 2013, Liu et al 2013 |  |
| mir-92a | Doebele et al 2010, Shigoka et al 2010, Haug et al 2011, Li et al 2012, Nilsson et al 2012, Attar et al 2012, Zhou et al 2013, Anand et al 2011 |  |
| mir-93 | Yeung et al 2008, Li et al 2009, Du et al 2009, Fang et al 2011, Yu et al 2011, Smith et al 2012, Fu et al 2012, Chuang et al 2012, Liu et al 2012 | x |
| mir-938 | Butz et al 2011, Arisawa et al 2012 |  |
| mir-99a | Sun et al 2011, Oneyama et al 2011, Li et al 2011, Turcatel et al 2012, Chen et al 2012, Yan et al 2012, Cui et al 2012, Sun et al 2013, Parker et al 2013, Babic et al 2013 |  |
| mir-99b | Kang et al 2012, Turcatel et al 2012, Tanic et al 2012, Lionetti et al 2009 |  |
