# Supplementary material for: Systemically Circulating Viral and Tumor-Derived MicroRNAs in KSHV-Associated Malignancies
Source: PLoS Pathog. 2013 Jul 18;9(7):e1003484. doi: 10.1371/journal.ppat.1003484 (PMC3715412; doi:10.1371/journal.ppat.1003484)
Supplement: Table S3 — Species cross-reactivity of Taqman microRNA assays. Product information from Life Technologies, Applied Biosystems. Recommended controls and 158 of the Taqman microRNA assays are shown with their mature microRNA sequence, Taqman assay name and species cross-reactivity for human, mouse and rat. Many of the assays exhibit substantial species cross-reactivity due to the conservation of microRNA sequences. (PDF) [file ppat.1003484.s020.pdf]

Table S3

TaqMan® MicroRNA Assays Human Panel - Early Access Kit		Species Targeted by Assay (Sanger miRNA Registry 9/04, Version 5.0)		
AB Assay Name	Mature miRNA Sequence (Assay Target)	Human	Mouse	Rat
hsa-let-7a	ugagguaguagguuguauaguu	Yes	Yes	Yes
hsa-let-7b	ugagguaguagguugugugguu	Yes	Yes	Yes
hsa-let-7d	agagguaguagguugcauagu	Yes	Yes	Yes
hsa-let-7e	ugagguaggagguuguauagu	Yes	Yes	Yes
hsa-let-7g	ugagguaguaguuuguacagu	Yes	Yes	
hsa-let-7i	ugagguaguaguuugugcu	Yes	Yes	Yes
hsa-miR-9	ucuuugguuaucuagcuguauga	Yes	Yes	Yes
hsa-miR-9*	uaaagcuagauaaccgaaagu	Yes	Yes	
hsa-miR-10a	uacccuguagauccgaauuugug	Yes	Yes	Yes
hsa-miR-15a	uagcagcacauaaugguuugug	Yes	Yes	
hsa-miR-15b	uagcagcacaucaugguuuaca	Yes	Yes	Yes
hsa-miR-16	uagcagcacguaaauauuggcg	Yes	Yes	Yes
hsa-miR-17-3p	acugcagugaaggcacuugu	Yes		
hsa-miR-17-5p	caaagugcuuacagugcagguagu	Yes	Yes	Yes
hsa-miR-19a	ugugcaaaucuaugcaaaacuga	Yes	Yes	Yes
hsa-miR-20	uaaagugcuuauagugcaggua	Yes		
hsa-miR-21	uagcuuaucagacugauguuga	Yes	Yes	Yes
hsa-miR-23a	aucacauugccagggauuucc	Yes	Yes	Yes
hsa-miR-23b	aucacauugccagggauuaccac	Yes	Yes	Yes
hsa-miR-25	cauugcacuugucucggucuga	Yes	Yes	Yes
hsa-miR-26a	uucaaguaauccaggauaggcu	Yes	Yes	Yes
hsa-miR-26b	uucaaguaauucaggauaggu	Yes		
hsa-miR-27a	uucacaguggcuaaguuccgcc	Yes		
hsa-miR-27b	uucacaguggcuaaguucug	Yes	Yes	Yes
hsa-miR-28	aaggagcucacagucuauugag	Yes	Yes	Yes
hsa-miR-29a	cuagcaccaucugaaaucgguu	Yes	Yes	Yes
hsa-miR-29b	uagcaccauuugaaaucagu	Yes		
hsa-miR-29c	uagcaccauuugaaaucgguua	Yes	Yes	Yes
hsa-miR-30a-3p	cuuucagucggauguuugcagc	Yes	Yes	Yes
hsa-miR-30b	uguaaacauccuacacucagc	Yes	Yes	Yes
hsa-miR-30c	uguaaacauccuacacucucagc	Yes	Yes	Yes
hsa-miR-30d	uguaaacauccccgacuggaag	Yes	Yes	Yes
hsa-miR-30e	uguaaacauccuugacugga	Yes	Yes	Yes
hsa-miR-31	ggcaagaugcuggcauagcug	Yes		
hsa-miR-34a	uggcagugucuuagcugguugu	Yes		
hsa-miR-34b	aggcagugucauuagcugauug	Yes		
hsa-miR-34c	aggcaguguaguuagcugauug	Yes		
hsa-miR-92	uauugcacuugucccggccugu	Yes		
hsa-miR-95	uucaacggguauuuauugagca	Yes		
hsa-miR-96	uuuggcacuagcacauuuuugc	Yes		
hsa-miR-98	ugagguaguaaguuguauuguu	Yes	Yes	Yes
hsa-miR-99a	aacccguagauccgaucuugug	Yes		Yes
hsa-miR-100	aacccguagauccgaacuugug	Yes	Yes	Yes
hsa-miR-103	agcagcauuguacagggcuauga	Yes	Yes	Yes
hsa-miR-104	ucaacaucagucugauaagcua	Yes		

hsa-miR-105	ucaaaugcucagacuccugu	Yes		
hsa-miR-106a	aaaagugcuuacagugcagguagc	Yes		
hsa-miR-107	agcagcauuguacagggcuauca	Yes	Yes	Yes
hsa-miR-122a	uggagugugacaaugguguuugu	Yes	Yes	Yes
hsa-miR-124a	uuaaggcacgcggugaaugcca	Yes	Yes	Yes
hsa-miR-124b	uuaaggcacgcggugaaugc	Yes		
hsa-miR-125a	ucccugagacccuuuaaccugug	Yes	Yes	Yes
hsa-miR-125b	ucccugagacccuaacuuguga	Yes	Yes	Yes
hsa-miR-126	ucguaccgugaguaauaaugc	Yes	Yes	Yes
hsa-miR-127	ucggauccgucugagcuuggcu	Yes	Yes	Yes
hsa-miR-128a	ucacagugaaccggucucuuuu	Yes	Yes	Yes
hsa-miR-128b	ucacagugaaccggucucuuuc	Yes	Yes	Yes
hsa-miR-129	cuuuuugcggucugggcuugc	Yes		
hsa-miR-130a	cagugcaauguuaaaagggc	Yes	Yes	Yes
hsa-miR-130b	cagugcaaugaugaaagggcau	Yes	Yes	Yes
hsa-miR-132	uaacagucuacagccauggucg	Yes	Yes	Yes
hsa-miR-133a	uuggucccuucaaccagcugu	Yes	Yes	Yes
hsa-miR-133b	uuggucccuucaaccagcua	Yes	Yes	
hsa-miR-134	ugugacugguugaccagaggg	Yes	Yes	Yes
hsa-miR-135a	uauggcuuuuuauuccuauguga	Yes	Yes	Yes
hsa-miR-135b	uauggcuuuucauuccuaugug	Yes	Yes	Yes
hsa-miR-137	uauugcuuaagaauacgcguag	Yes	Yes	Yes
hsa-miR-138	agcugguguugugaauc	Yes	Yes	Yes
hsa-miR-139	ucuacagugcacgugucu	Yes	Yes	Yes
hsa-miR-140	agugguuuuacccuaugguag	Yes	Yes	Yes
hsa-miR-141	aacacugucugguaaagaugg	Yes	Yes	Yes
hsa-miR-142-3p	uguaguguuuccuacuuuaugga	Yes		Yes
hsa-miR-142-5p	cauaaaguagaaagcacuac	Yes	Yes	Yes
hsa-miR-144	uacaguauagaugauguacuag	Yes	Yes	Yes
hsa-miR-145	guccaguuuucccaggaaucccuu	Yes	Yes	Yes
hsa-miR-146	ugagaacugaauuccauggguu	Yes	Yes	Yes
hsa-miR-147	guguguggaaaugcuucugc	Yes		
hsa-miR-148a	ucagugcacuacagaacuuugu	Yes	Yes	
hsa-miR-149	ucuggcuccgugucuucacucc	Yes	Yes	
hsa-miR-150	ucucccaacccuuguaccagug	Yes	Yes	Yes
hsa-miR-151	acuagacugaagcuccuugagg	Yes		
hsa-miR-152	ucagugcaugacagaacuugg	Yes	Yes	Yes
hsa-miR-154	uagguuauccguguugccuucg	Yes	Yes	Yes
hsa-miR-154*	aaucauacacgguugaccuauu	Yes		
hsa-miR-155	uuaaugcuaaucgugauagggg	Yes		
hsa-miR-181a	aacauucaacgcugucggugagu	Yes	Yes	Yes
hsa-miR-181b	aacauucauugcugucgguggguu	Yes	Yes	Yes
hsa-miR-181c	aacauucaaccugucggugagu	Yes	Yes	Yes
hsa-miR-182	uuuggcaaugguagaacucaca	Yes	Yes	
hsa-miR-182*	ugguucuagacuugccaacua	Yes		
hsa-miR-183	uauggcacugguagaauucacug	Yes	Yes	Yes
hsa-miR-184	uggacggagaacugauaagggu	Yes	Yes	Yes
hsa-miR-185	uggagagaaaggcaguuc	Yes	Yes	Yes
hsa-miR-186	caaagaauucuccuuuugggcuu	Yes	Yes	Yes
hsa-miR-187	ucgugucuuguguugcagccg	Yes		Yes
hsa-miR-189	gugccuacugagcugauaucagu	Yes	Yes	

hsa-miR-190	ugauauguuugauauauuaggu	Yes	Yes	Yes
hsa-miR-191	caacggaaucccaaaagcagcu	Yes	Yes	Yes
hsa-miR-193	aacuggccuacaaagucccag	Yes	Yes	Yes
hsa-miR-194	uguaacagcaacuccaugugga	Yes	Yes	Yes
hsa-miR-195	uagcagcacagaaauauuggc	Yes	Yes	Yes
hsa-miR-197	uucaccaccuucuccacccagc	Yes		
hsa-miR-198	gguccagaggggagauagg	Yes		
hsa-miR-199a	cccaguguucagacuaccuguuc	Yes	Yes	Yes
hsa-miR-199a*	uacaguagucugcacauugguu	Yes	Yes	
hsa-miR-199b	cccaguguuuagacuaucuguuc	Yes		
hsa-miR-199-s	cccaguguucagacuaccuguu	Yes		
hsa-miR-200a	uaacacugucugguaacgaugu	Yes	Yes	Yes
hsa-miR-200b	cucuaauacugccugguaaugaug	Yes		Yes
hsa-miR-200c	aauacugccggguaaugaugga	Yes	Yes	Yes
hsa-miR-203	gugaaauguuuaggaccacuag	Yes		Yes
hsa-miR-204	uucccuuugucauccuaugccu	Yes		Yes
hsa-miR-205	uccuucauuccaccggagucug	Yes	Yes	Yes
hsa-miR-210	cugugcgugugacagcggcug	Yes	Yes	Yes
hsa-miR-211	uucccuuugucauccuucgccu	Yes		
hsa-miR-213	accaucgaccguugauuguacc	Yes	Yes	Yes
hsa-miR-214	acagcaggcacagacaggcag	Yes	Yes	Yes
hsa-miR-215	augaccuaugaauugacagac	Yes		
hsa-miR-216	uaaucucagcuggcaacugug	Yes	Yes	Yes
hsa-miR-218	uugugcuugaucuaaccaugu	Yes	Yes	Yes
hsa-miR-219	ugauuguccaaacgcaauucu	Yes	Yes	Yes
hsa-miR-220	ccacaccguaucugacacuuu	Yes		
hsa-miR-221	agcuacauugucugcuggguuuc	Yes		Yes
hsa-miR-222	agcuacaucuggcuacugggucuc	Yes	Yes	Yes
hsa-miR-223	ugucaguuugucaaauacccc	Yes	Yes	Yes
hsa-miR-224	caagucacuagugguuccguuua	Yes		
hsa-miR-296	agggcccccccucaauccugu	Yes	Yes	Yes
hsa-miR-299	ugguuuaccgucccacauacau	Yes	Yes	Yes
hsa-miR-301	cagugcaauaguauugucaaagc	Yes	Yes	
hsa-miR-302a	uaagugcuuccauguuuugguga	Yes	Yes	
hsa-miR-302b	uaagugcuuccauguuuuaguag	Yes		
hsa-miR-302b*	acuuuaacauggaagugcuuucu	Yes		
hsa-miR-302c	uaagugcuuccauguuucagugg	Yes		
hsa-miR-302c*	uuuaacauggggguaccugcug	Yes		
hsa-miR-302d	uaagugcuuccauguuugagugu	Yes		
hsa-miR-320	aaaagcuggguugagagggcgaa	Yes	Yes	Yes
hsa-miR-323	gcacauuacacggucgaccucu	Yes	Yes	Yes
hsa-miR-324-5p	cgcauccccuagggcauuggugu	Yes	Yes	Yes
hsa-miR-325	ccuaguagguguccaguaagu	Yes		
hsa-miR-326	ccucugggcccuuccuccag	Yes		
hsa-miR-328	cuggcccucucugcccuuccgu	Yes	Yes	Yes
hsa-miR-330	gcaaagcacacggccugcagaga	Yes		
hsa-miR-331	gccccugggccuauccuagaa	Yes	Yes	Yes
hsa-miR-335	ucaagagcaauaacgaaaaaugu	Yes	Yes	Yes
hsa-miR-337	uccagcuccuauaugaugccuuu	Yes		
hsa-miR-338	uccagcaucagugauuuuguuga	Yes	Yes	Yes
hsa-miR-339	ucccuguccuccaggagcuca	Yes	Yes	Yes

hsa-miR-340	uccgucucaguuacuuuauagcc	Yes	Yes	Yes
hsa-miR-342	ucucacacagaaaucgcacccguc	Yes	Yes	Yes
hsa-miR-367	aauugcacuuuagcaauggugа	Yes		
hsa-miR-368	acauagaggaaauuccacguuu	Yes		
hsa-miR-370	gccugcuggggguggaaccugg	Yes		
hsa-miR-371	gugccgccaucuuuugagugu	Yes		
hsa-miR-372	aaagugcugcgacauuugagcgu	Yes		
hsa-miR-373	gaagugcuucgauuuuggggugu	Yes		
hsa-miR-373*	acucaaaaugggggcgcuuucc	Yes		
hsa-miR-374	uuauaauacaaccugauaagug	Yes		
ath-miR159a	uuuggauugaagggagcucua	*Arabidopsis* negative control		
cel-lin-4	ucccugagaccucaaguguga	*C. elegans* negative control		
cel-miR-2	uaucacagccagcuuugaugugc	*C. elegans* negative control		
