# Supplementary material for: Systemically Circulating Viral and Tumor-Derived MicroRNAs in KSHV-Associated Malignancies
Source: PLoS Pathog. 2013 Jul 18;9(7):e1003484. doi: 10.1371/journal.ppat.1003484 (PMC3715412; doi:10.1371/journal.ppat.1003484)
Supplement: Table S4 — Ingenuity Pathway Analysis of predicted oncomir targets. Oncomirs used in the microRNA profiling array and induced in KSHV-associated exosomes were used as input for Ingenuity Pathway Analysis (www.ingenuity.com/products/pathways_analysis.html). Predicted targets were obtained using Ingenuity and were narrowed down to include only experimentally validated targets. These targets were then overlayed with the functional pathway tool and the top represented pathways are shown. The number of target genes involved in each pathway is shown along with the univariate p value as determined by Ingenuity software. Several pathways are bolded to denote their importance in tumorigenesis and KSHV pathogenesis. As a control, we used miRNAs induced by WNV infection of hTERT-HUVEC cells. (DOCX) [file ppat.1003484.s021.docx]

**Table S4. Ingenuity Pathway Analysis of Predicted Exosome Oncomir Targets**

| **Functional Annotation** | **Exosome**  **Oncomirs# of genes** | **P value** | **WNV Induced mirs**  **# of genes** | **P value** |
| --- | --- | --- | --- | --- |
| **Cell cycle progression** | 57 | E-55 | 1 | E-04 |
| Cell death of immune cells | 50 | E-50 | - | 1 |
| Differentiation of cells | 61 | E-49 | - | 1 |
| **Cell transformation** | 42 | E-48 | 1 | E-04 |
| Cell survival | 56 | E-46 | 1 | E-03 |
| **Proliferation of tumor cell lines** | 57 | E-46 | - | 1 |
| Differentiation of blood cells | 46 | E-46 | - | 1 |
| Cell viability | 54 | E-44 | 2 | E-04 |
| Cell death of tumor cell lines | 57 | E-43 | - | 1 |
| **Apoptosis** | 64 | E-41 | 2 | E-05 |
| Apoptosis of leukocytes | 39 | E-39 | - | 1 |
| Proliferation of immune cells | 44 | E-39 | - | 1 |
| **Development of tumor** | 32 | E-37 | - | 1 |
| Development of lymphocytes | 36 | E-36 | - | 1 |
| Proliferation of lymphocytes | 40 | E-35 | - | 1 |
| Cell death of tumor cells | 34 | E-34 | - | 1 |
| **Colony formation of cells** | 33 | E-33 | 1 | E-04 |
| Cell death of leukocyte cell lines | 28 | E-33 | - | 1 |
| Differentiation of tumor cell lines | 31 | E-32 | - | 1 |
| Cell cycle progression of tumor cell lines | 27 | E-31 | - | 1 |
| **Cell death of cancer cells** | 30 | E-31 | - | 1 |
| **Invasion of cells** | 36 | E-30 | 3 | E-04 |
| **Contact growth inhibition** | 23 | E-30 | 1 | E-04 |
| **Cancer** | 61 | E-29 | 2 | E-04 |
| Senescence of cells | 24 | E-29 | 1 | E-04 |
| **Tumorigenesis of cells** | 23 | E-28 | 1 | E-04 |
| **Cell movement** | 49 | E-28 | 1 | E-04 |
| **Migration of cells** | 47 | E-28 | 1 | E-04 |
| **Progression of tumor** | 20 | E-27 | - | 1 |
| **Growth of tumor** | 28 | E-26 | - | 1 |
| **Colony formation of tumor cell lines** | 22 | E-24 | - | 1 |
