# Supplementary material for: Systemically Circulating Viral and Tumor-Derived MicroRNAs in KSHV-Associated Malignancies
Source: PLoS Pathog. 2013 Jul 18;9(7):e1003484. doi: 10.1371/journal.ppat.1003484 (PMC3715412; doi:10.1371/journal.ppat.1003484)
Supplement: Table S5 — Pathway analysis of predicted targets using the Panther Database. Predicted targets were obtained through the Ingenuity program and only included experimentally validated microRNA targets. The Entrez IDS of predicted targets were used as input for the Panther database (www.pantherdb.org) [114]. Pathway analysis was performed and the number of target genes involved in the top pathways are denoted. WNV-induced predicted targets were used as a control experiment and revealed different target pathways. The total number of predicted target genes for the KS-associated exosomal microRNAs and WNV-induced microRNAs were 188 and 106, respectively. Differences between KSHV oncomir targets and WNV-induced targets were significant as determined by paired T test (p = 1.56E-06). (DOCX) [file ppat.1003484.s022.docx]

**Table S5. Pathway Analysis of Predicted Targets using the Panther Database**

*Differences between KSHV oncomir targets and WNV-induced targets were significant (p=1.56E-06).­­

| **Signaling Pathway** | **Number of Genes** | **WNV Targeted Genes** |
| --- | --- | --- |
| Angiogenesis | 18 | 2 |
| p53 Pathway | 13 | 1 |
| Apoptosis | 12 | 2 |
| Interleukin Signaling Pathway | 12 | 3 |
| EGF Receptor Signaling Pathway | 12 | 2 |
| TGF-beta Signaling Pathway | 11 | 6 |
| PDGF Signaling Pathway | 11 | 3 |
| Chemokine/Cytokine Signaling | 10 | 3 |
| Ras Pathway | 10 | 1 |
| FGF Signaling Pathway | 10 | 1 |
| PI3 Kinase Pathway | 10 | 2 |
| Integrin Signaling Pathway | 7 | 2 |
| Wnt Signaling Pathway | 7 | 12 |
| Hypoxia Response (HIF) | 6 | 1 |
| VEGF Signaling Pathway | 6 | 1 |
| FAS Signaling Pathway | 6 | 0 |
| JAK/STAT Signaling Pathway | 5 | 2 |
| Notch Signaling Pathway | 5 | 0 |
| Interferon-gamma Signaling Pathway | 4 | 1 |
| p38 MAPK Pathway | 4 | 1 |
| Oxidative Stress Response | 4 | 2 |
| Cadherin Signaling Pathway | 4 | 7 |
| B Cell Activation | 4 | 1 |
| Toll Receptor Signaling Pathway | 3 | 0 |
| Cell Cycle | 3 | 0 |
